# Deciphering Size and Shape Effects on the Structure Sensitivity of the CO_2_ Methanation Reaction on Nickel

**DOI:** 10.1021/acscatal.4c08084

**Published:** 2025-05-02

**Authors:** Gabriele Spanò, Matteo Ferri, Raffaele Cheula, Matteo Monai, Bert M. Weckhuysen, Matteo Maestri

**Affiliations:** † Laboratory of Catalysis and Catalytic Processes, Dipartimento di Energia, Politecnico di Milano, Via La Masa, 34, 20156 Milano, Italy; ‡ Inorganic Chemistry and Catalysis Group, Institute for Circular and Sustainable Chemistry, Utrecht University, Universiteitsweg 99, 3584 CG Utrecht, The Netherlands

**Keywords:** Sabatier reaction, nickel, structure sensitivity, density functional theory, nanoparticle ensemble

## Abstract

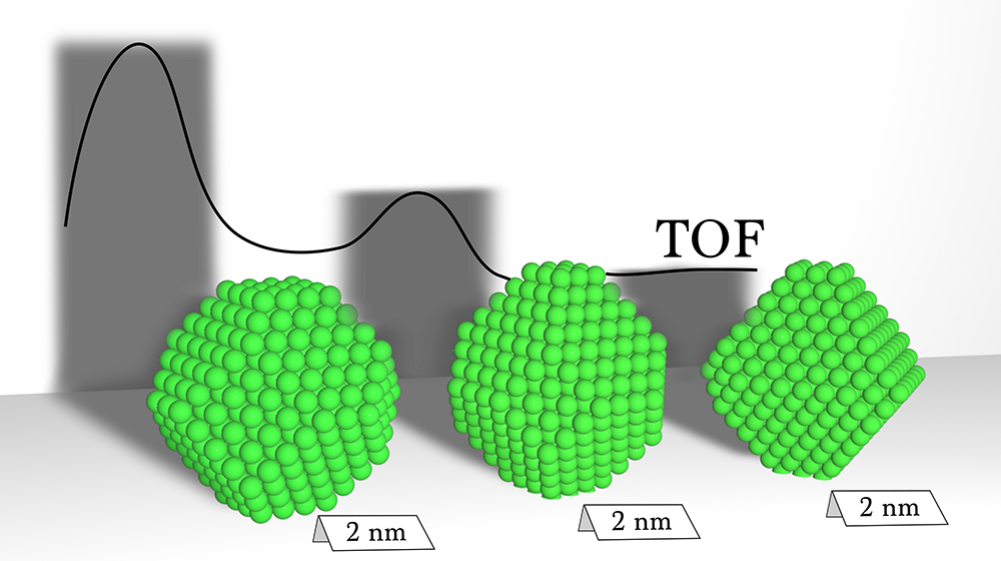

This study advances the understanding of structure sensitivity in CO_2_ methanation over nickel-based catalysts by highlighting the combined influence of the metal nanoparticle (NP) size and shape on catalytic performance. Density functional theory (DFT) calculations of the metal nanoparticle structure and activity provide the theoretical underpinnings of the experimentally observed structure sensitivity of CO_2_ methanation over nickel-based catalysts. This is achieved by taking into account the diversity of shapes of metal nanoparticles (NPs) under the reaction conditions and the corresponding distribution of active sites at different metal NP sizes. We built a large ensemble of Ni metal NPs with different shapes and sizes in the range of 0.5–10 nm and quantified the distribution of the potential active sites for each NP. We then computed the reaction rate over each of these active sites on the metal surface to evaluate the activity as a function of the metal NP diameter. Our calculations reveal that the activity at the active sites located at the edge between the Ni(100) and Ni(111) facets largely dominates the overall observed activity. Furthermore, metal NPs can be categorized into families based on their shape, specifically the fraction of exposed Ni(100) facets. The observed maximum in turnover frequency (TOF) for 2–3 nm metal NPs is linked to the dominance of NP families with high Ni(100) fractions. Conversely, experimental conditions favoring NP families with higher Ni(111) fractions result in a hockey stick trend in the TOF. These findings resolve key debates on structure sensitivity in CO_2_ methanation and offer broader applicability to other structure-sensitive reactions, such as ammonia synthesis, decomposition, and Fischer–Tropsch synthesis, where similar sensitivities have been widely debated.

## 1 Introduction

Metal nanoparticles (NPs) supported on various support materials, including silica, alumina, titania, and zeolites, are vital as heterogeneous catalysts in numerous chemical reactions. The dependence of turnover frequency (TOF) on the structure of these metal NPs is a defining characteristic of the concept commonly referred to as structure sensitivity.^1,2^ Specifically, structure sensitivity is the result of the interplay between morphological and electronic factors: while the abundance of the different active sites is determined by the NP morphology (i.e., shape and dimension), the activity of each active site depends on the electronic interaction energy with the kinetically relevant reaction intermediates.^3,4^ The modern view is that structure sensitivity is caused by the abundance of important active sites, as a function of the metal NP size.

The CO_2_ methanation on Ni catalysts, also known as the Sabatier reaction in which CO_2_ and H_2_ react to form CH_4_ and H_2_O, is a promising solution for long-term and large-scale renewable energy storage.^5^ This reaction has been reported to exhibit structure sensitivity by several investigators.^1,6−8^ Conflicting reports, however, are present regarding the trend of the TOF at varying metal NP diameters.^7−10^ For instance, Vogt et al. synthesized a set of SiO_2_-supported Ni metal nanoparticles with different sizes, ranging from 1 to 7 nm, and observed a maximum in the catalytic activity around 2.5 nm.^8^ Similarly, Simons et al. identified a comparable maximum in CH_4_ formation rates on Ni/SiO_2_, attributing the variation in methanation activity to changes in the number of reactive surface sites as a function of particle size.^9^ In contrast, Visser et al. reported a continuous increase in catalytic activity with particle size for CO_2_ methanation over Ni/OxC, without observing a maximum in TOF relative to particle dimension.^10^ Based on these premises, it is clear that there is not yet a consensus in the literature regarding the nature and origin of the variations in catalytic activity as a function of metal particle size in CO_2_ methanation over Ni-based catalysts.

A first structure-dependent microkinetic model of the CO_2_ methanation reaction over Ni was developed by Sterk et al. in an effort to shed light on the nature and origin of the structure sensitivity of the reaction.^11^ Specifically, some of us adopted the Wulff construction method to describe the structure of the Ni metal nanoparticles. The reaction mechanism of the CO_2_ methanation process has been studied over extended Ni surfaces^11−17^ and the overall catalytic activity of Ni metal nanoparticles has been computed as a linear combination of the catalytic activity of the individual facets.^18^ To replicate the experimental trend in structure sensitivity, an arbitrarily high kinetic response was assigned to the undercoordinated atoms, which could not be specifically attributed to any particular facet of the Ni metal nanoparticle.^11^ These findings suggest the importance of undercoordinated sites in determining the overall catalytic performance.^2,16,19−25^

While, on one hand, this work explains the maximum trend of TOF with particle size, on the other hand, the Wulff-construction method employed provides only the ground-state shape of the metal NPs, irrespective of their size.^13^ As a result, the use of this size-independent technique fails to account for the possible impact of varying the NP shapes on catalytic activity across different NP diameters. In general, multiple factors may influence the shape of metal NPs at the same particle diameter, including the choice of metal-oxide support^26^ or preparation method, both of which can affect the dispersion of the active phase or even alter the reaction mechanism.^27,28^ Therefore, in analyzing the structure sensitivity of the reaction, it is crucial to explicitly account for changes in NP shape at varying particle diameters, considering the diverse shapes that metal NPs can adopt under reaction conditions.^27,29^ This requires moving beyond the equilibrium shape of the metal NPs provided by the Wulff construction.

To this aim, in this work, we employ the methodology of Cheula et al.,^13^ which involves an automatic procedure to obtain atomistic models of metal NPs. By a combination of density functional theory (DFT) with Boltzmann statistics, this methodology identifies the distribution of the active sites under reaction conditions across different metal NP sizes. Differently from the Wulff construction method, which provides only the equilibrium shape, here we explore, for each size, all of the possible shapes that may better accommodate the active sites. This flexibility overcomes the limitations of Wulff-constructed metal NPs, where the shape is fixed for a given size. Moreover, for any size, the rigid equilibrium shape constrains the atoms to fit precisely within it. Consequently, an ensemble composed solely of Wulff-constructed particles lacks continuity with respect to the number of atoms and thus with the NP size. This may lead to wrong estimations of the active site distribution across different metal NP diameters.

Therefore, we build a large ensemble of Ni NPs with different shapes and sizes in the range of 0.5–10 nm and quantify the distribution of surface-active sites for each of these NPs. The contribution of the abundance of the active sites of the statistically relevant metal NPs is then combined with the reaction rate over each site on the metal surface. We obtain a global turnover frequency (TOF) for CO_2_ methanation over Ni with a maximum of around 2.5 nm, in excellent agreement with experimental observations at the corresponding operating conditions.^8^ To elucidate the origin of this maximum, we classify the metal NPs in different families according to their shape, specifically, the fraction of exposed Ni(100) facets. Notably, NPs with different shapes can exist for the same diameter with a high probability of occurrence. Moreover, the different NP families exhibit differing trends for the TOF. NPs with relatively high Ni(100) fractions display higher TOFs and a pronounced maximum in activity for NPs in the 2–3 nm size range. In contrast, families with a lower fraction of Ni(100) 4-fold sites exhibit lower TOFs, following a “hockey stick” behavior where the TOF reaches a plateau at increasing NP size. As a result, the presence of a maximum in the TOF at increasing NP size is primarily attributed to the TOF trend of the dominant family of NP particles, i.e., the metal NPs with a high fraction of 4-fold sites. Therefore, while previous investigations assigned an arbitrary kinetic response to undercoordinated sites, here we provide a consistent framework based on site-specific activities which offers an explanation for the diverse trends in TOF for CO_2_ methanation reaction observed across different NP sizes.^11^

More broadly, this work provides theoretical insights into the nature and origin of the structure sensitivity in heterogeneous catalysis. Structure sensitivity is influenced not only by the size of the NPs, but also by slight changes in their shape. The shape, in turn, can be affected by the catalyst preparation method or the choice of support material used to disperse the active phase.^27^ Furthermore, these methods and concepts can be directly applied to other classes of structure-sensitive reactions such as ammonia synthesis and decomposition.

## 2 Methods

### 2.1 Density Functional Theory Calculations

DFT calculations are performed with the Quantum Espresso (QE) suite of codes^30−32^ using the Perdew–Burke–Ernzerhof (PBE)^33^ formulation of the electron exchange-correlation functional. Due to the ferromagnetic properties of Ni, all calculations are performed including collinear spin polarization. We use ultrasoft pseudopotentials from the Standard Solid-State Pseudopotential (SSSP) library^34,35^ and we set a kinetic energy cutoff of 50 Ry (∼680 eV) and 300 Ry (∼4080 eV) for the planewaves and the charge density, respectively, to converge the total energy per atom within 0.01 eV. Periodic slabs are employed to model the local environment and the different active sites of the metal NPs, as shown in Figures S3–S13. The slabs are composed of 5 layers, which are periodically repeated in the *xy* plane with a vacuum region of 12 Å separating the replicas along the *z* direction. The two lowest layers are kept fixed, while the three topmost layers and the adsorbates therein are free to relax. The geometry of slabs and adsorbates is optimized using the Broyden–Fletcher–Goldfarb–Shanno (BFGS) algorithm, with a convergence threshold of 10^–3^ Ry Bohr^–1^ on the forces and 10^–4^ Ry on the energies. We sample the Brillouin zone (BZ) of the face-centered cubic (fcc) bulk Ni with a uniform Monkhorst–Pack grid^36^ of 8 × 8 × 8 k-points, while the BZs of the slabs are sampled in such a way to keep the same kspacing as in the bulk. The climbing-image nudged elastic band (CI-NEB)^37^ calculations are adopted to identify the transition states, setting the number of intermediate images in such a way as to have an average interimage distance around 1.2 Bohr, and a force convergence threshold of 0.05 Ry Å^–1^. Vibrational analysis of the stationary points and the transition states are then performed within the harmonic approximation using the finite differences method as implemented in the Atomic Simulation Environment (ASE) library,^38^ applying displacements of 0.01 Å to each atom of the adsorbed species. We checked the presence of a single imaginary frequency in the vibrational spectrum of transition states.

### 2.2 Ensemble Model of the Nickel–Metal Nanoparticles

We identify the statistically relevant metal NPs within an ensemble of Ni metal NPs of different shapes and sizes. The ensemble is generated following the procedure of Cheula et al.,^13^ namely cleaving a bulk Ni lattice with combinations of low- and high-Miller indices lattice planes, whose distance from the center of the construction is set to integer multiples of the interplanar distance of the corresponding facet. Iterating these numbers to cover all the possible combinations below a certain threshold, it is possible to generate metal NPs exposing all of the combinations of the surfaces under consideration. In small NPs, twinning and strain effects may give rise to pseudomorphous structures, thus necessitating the inclusion of strain-dependent parameters for accurate evaluation of their formation energies.^39−41^ In this respect, prior findings on Rh NPs demonstrated the dominance of fcc structures, with icosahedral and decahedral forms remaining stable only in specific atomic arrangements,^13^ e.g., Rh_55_ and Rh_147_. To ensure a rigorous assessment before assuming a similar trend for Ni NPs, we performed DFT relaxation calculations for the pseudomorphous Ni_55_ and Ni_147_ configurations. Their formation energies result in being significantly higher than those of fcc NPs with the same atomic count (Figure S27). This confirms that such structures are unlikely to contribute meaningfully to the overall ensemble. Consequently, we exclude these configurations from our model but maintain physical relevance in our sampling strategy. The resulting ensemble consists of more than 60,000 metal NPs containing from 80 to 16,000 Ni atoms and spanning a range of sizes between 0.8 and 6.5 nm, where the structure sensitivity is expected to play a role. The catalytic activity of an ensemble of nanoclusters can be directly simulated by first-principles methods for particles with less than 100 atoms.^42−44^ Since for larger NPs the calculation of the DFT energy of the metal NPs is extremely computationally demanding, we adopt a surrogate atomistic model to compute the formation energy of the metal NPs from their geometric configuration.^13^ The model is summarized in Sect. 5 of the SI and the predicted formation energies are benchmarked against the DFT energies of selected Ni slabs and small metal NPs, showing good agreement within 0.05 eV/atom as reported in the parity plot in Figure S22. The formation energies are used to evaluate the probability of occurrence *p*_*i*_ of the *i*th metal nanoparticle^18,44^ in the ensemble using a weighted Boltzmann distribution:
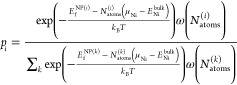
1where (μ_Ni_–*E*_Ni_^bulk^) represents a shift in the reference energy from the energy of bulk Ni to the chemical potential of the Ni atoms reservoir μ_Ni_,^13^ while ω(*N*_atoms_^(*i*)^) a Gaussian weight function, centered on a specific *N*_target_:
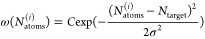
2

In eq 2, σ represents the standard deviation of the Gaussian function, and *C* is a normalization factor that cancels out when ω(*N*_atoms_^(*i*)^) is inserted into eq 1. A weighted distribution gives a larger weight to the metal NPs having a number of atoms close to *N*_target_. The physical meaning of eq 2 is to take a subset of metal NPs distributed around a given size. Values of σ between 5 and 10 provide distributions of diameters within 0.1 nm. For a fixed *N*_target_, the probability of occurrence of each metal NP is computed self-consistently using eq 1, varying μ in order to minimize the difference between the average of the number of atoms of the ensemble, defined as ⟨*N*_atoms_⟩ = ∑_*i*_*p*_*i*_*N*_atoms_^(*i*)^, and *N*_target_. Iterating the process for each *N*_target_ compatible with the range of the number of atoms of the metal NP in the ensemble, we identify the metal NPs with the highest probability of occurrence at any particle size. As shown in Figure S23b, certain metal NPs may have a high probability of occurrence for several values of *N*_target_, but with different probabilities. The generation of the ensemble of metal NPs and the statistical analysis of the relative stability is performed using a homemade Python code.

## 3 Results and Discussion

With the aim of elucidating the nature of structure sensitivity of the CO_2_ methanation reaction on nickel metal NPs, we need to separately evaluate the reaction rate over each site  and how the site fractions *f*_*i*_ change with the metal NP size at relevant experimental conditions.^8^ The catalytic activity of a metal NP per unit surface atom depends on the abundance of the active sites at the surface and the reaction rate over each active site.^45^ The reaction occurs in parallel over each site; thus, the total reaction rate over a specific metal nanoparticle is computed as
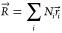
3where *i* runs over the different kinds of active site, *N*_*i*_ is the corresponding number of sites within the metal NP and  is the reaction rate over the active site. The overall rate is normalized by the number of surface atoms to get an expression for the experimentally observed TOF:
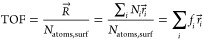
4where *f*_*i*_ = *N*_*i*_/*N*_atoms, surf_ represents the site fraction of the *i*th site over the metal NP. The activity A of a metal NP is defined as the overall rate reaction rate  in eq 3, normalized by the mass of the metal NP:
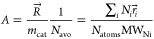
5where MW_Ni_ = 58.69 g/mol is the molecular weight of Ni and *N*_avo_ is the Avogadro constant.

**Figure 1:**
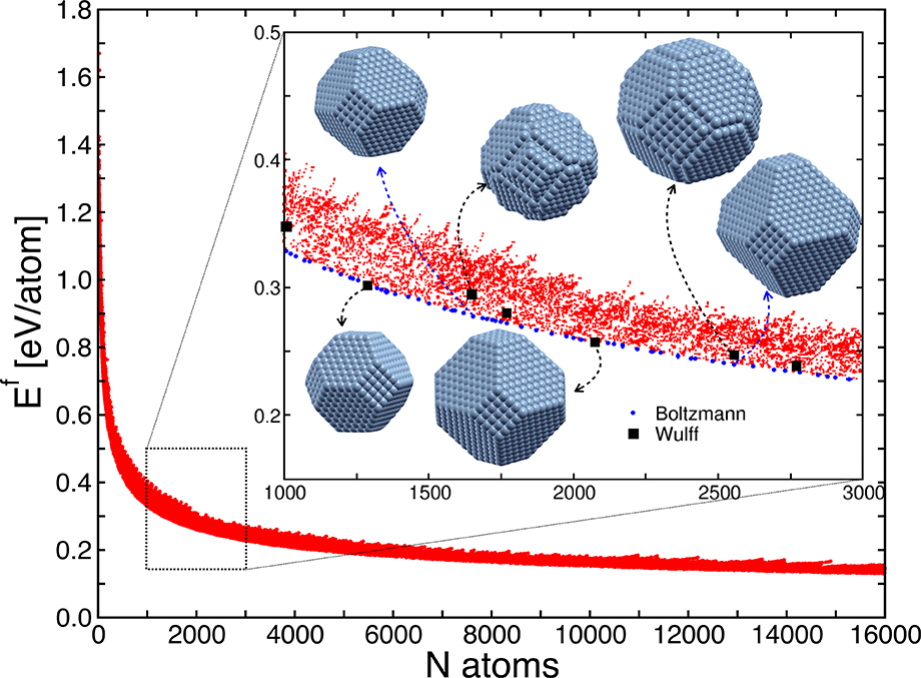
Formation energy of nickel metal nanoparticles (NPs) normalized per atom at 0 K, as a function of the number of atoms of the metal NP. In the inset we highlight with blue points the metal NPs, which show a probability of occurrence higher than 10% for at least one value of *N*_target_ in the range of *N*_atoms_ between 1000 and 3000, corresponding approximately to a particle size between 2.6 and 3.8 nm, in comparison with selected Wulff-constructed metal nanoparticles (black squares). The operating conditions in the Boltzmann distribution are taken as the experimental ones of Vogt et al.^8^ In particular, the temperature is set to 673 K, while for a total pressure of 1 bar, the inlet CO_2_:H_2_:inert ratio is 1:4:5, corresponding to partial pressures P_CO_2__^in^= 0.1 bar and P_H_2__^in^ 0.4 bar.

To compute the site fraction *f*_*i*_, we generate an ensemble of Ni metal NPs and we assess their relative stability by a combination of an atomistic energy model and Boltzmann statistics.^13^ The formation energies of the metal NPs in the ensemble normalized by the number of atoms within the metal NP are reported in Figure 1, where the *x*-axis indicates the average size of the metal NP with a certain number of Ni atoms, estimated in Sect. 5.1.1 of the SI. In the inset of Figure 1, we highlight with blue points the metal NPs, which show a high probability of occurrence (i.e., above 10%) for at least one value of *N*_target_ in the range of *N*_atoms_ between 1000 and 3000, corresponding approximately to a NP size between 2.6 and 3.8 nm, and with black squares the metal NPs constructed filling a Wulff plot at given size with Ni atoms,^12^ as implemented in ASE.^38^

As shown in Figure 1, there are several NPs with a high probability of occurrence ranging in size between two Wulff-constructed metal NPs. On one hand, this ensures a greater continuity with respect to the number of atoms of the statistically relevant NPs in the ensemble. On the other hand, this high granularity of NPs makes it possible to account for various shapes across different diameters, which deviate from the equilibrium one provided by the Wulff construction method. In particular, most of the NPs with the highest probability of occurrence are cuboctahedra showing Ni(111) and Ni(100) facets, as shown in the inset of Figure 1. We report in the upper part of the inset the crystal structure of two Wulff metal NPs with stepped edges which have a negligible probability of occurrence and the high-probability metal NPs having a similar number of atoms.^11^

The energy model is extended to account for the presence of adsorbates on the metal NP surface,^13^ as explained in Section 6 of the SI, since metal particle facets may undergo distinct structural changes in the presence of surface reaction intermediates.^46^ To ensure the accuracy of the model in predicting the energetic trends in the presence of CO*, the predicted formation energies are benchmarked against the DFT energies of selected Ni slabs and small metal NPs in conditions of low and high CO* coverage, showing a good agreement within 0.05 eV/atom as reported in the parity plot in Figure S22.

**Figure 2:**
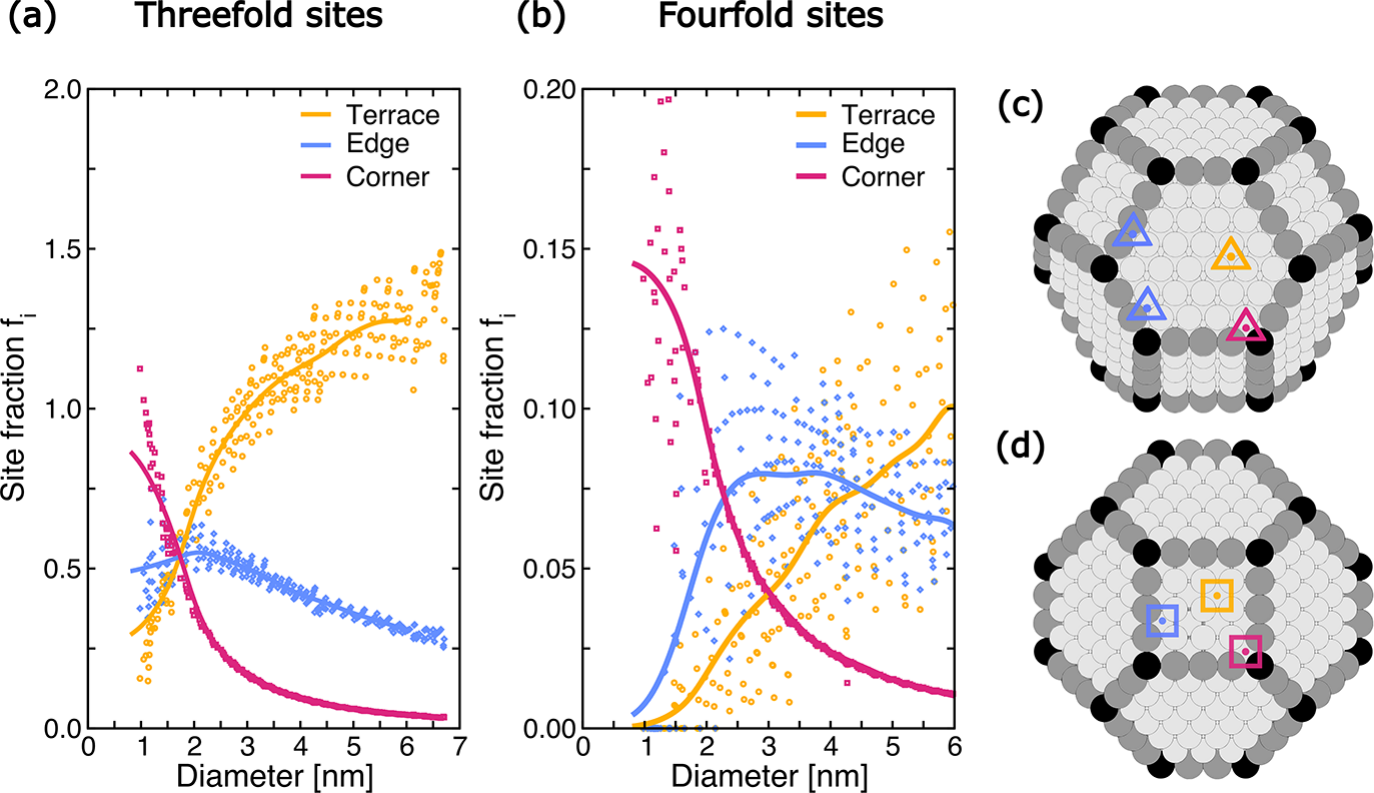
Distribution of 3-fold and 4-fold nickel surface sites as a function of the metal nanoparticle diameter on Ni(111) (a) and Ni(100) (b) metal nanoparticle facets. The different kinds of surface sites are represented in panel (c) and (d) for Ni(111) and the Ni(100) facet, respectively. Edge Ni atoms, with coordination number 7, and corner Ni atoms, with coordination number 6, are represented with gray and black spheres, respectively. Terrace, edge and corner Ni sites are depicted with yellow, blue and red symbols, respectively. A Gaussian smoothing is adopted to interpolate the points.

In particular, CO* is known to be responsible for the metal NP restructuring under reaction conditions.^12^ However, as shown in Figures S27 and S32, the shape of the high-probability metal NPs, hence the active site distribution, is not significantly affected by the presence of CO*. This is due to the fact that in Ni metal NPs, the adsorption of CO* over 3-fold and 4-fold coordinated active sites is energetically favored and contributes to stabilizing the facets, unlike for different metals, such as Rh, in which it adsorbs mainly on top of the atoms,^13^ facilitating the formation of stepped edges between adjacent facets. Ni metal NPs with stepped edges and corners show a high probability of occurrence above 3 nm, while the number of 3-fold and 4-fold active sites is not significantly affected by the inclusion of CO*, as shown in Figures S33–S35. We distinguish two main kinds of active sites on the surface of cuboctahedra according to the number of atoms that form the site. As shown in Figure 2c,d, Ni(111) facets are characterized by 3-fold coordinated fcc/hcp sites, while Ni(100) facets by 4-fold coordinated hollow sites. We further classify the active sites on the basis of the coordination number of the corresponding atoms that form the active site. Atoms in the center of Ni(111) facets have a coordination number of 9, while those in the center of Ni(100) have a coordination number of 8. Atoms at the corner and at the edge between two facets have a coordination number of 6 and 7, respectively, and are depicted with black and gray spheres in Figure 2c,d.

Three families of active sites can then be distinguished: (i) terrace sites, in which all the constituent atoms lie only in the center of the facet, (ii) edge sites, in which at least one atom lies at the edge, with coordination number 7 and (iii) corner sites, formed by at least one atom at the corner, with coordination number 6. Representative active sites of the three families are indicated with yellow, blue, and red triangles and squares in Figure 2c,d for 3-fold and 4-fold sites, respectively. The site fractions *f*_*i*_ of these active sites in the high-probability metal NPs are reported in Figure 2a,b as functions of the metal NP size. To discern a trend in the site fraction *f*_*i*_ we apply a Gaussian smoothing interpolation across the data points. Hence, the trend of *f*_*i*_ versus NP size differs among the active sites. The fraction of corner sites decreases monotonically with the NP size, showing a large fraction in the smallest metal NPs, due to the limited size of the exposed facets. Conversely, the fraction of 4-fold terrace sites increases monotonically with the NP size starting from 1 nm. The fractions of 3-fold edge sites and 4-fold edge sites exhibit a maximum at ∼1.1 and ∼2.4, respectively. Remarkably, the percentage of the 3-fold sites in the high-probability metal NPs falls between 83 and 97%, with the remaining fraction attributed to 4-fold hollow sites (as reported in Figure S24). Therefore, the value of *f*_*i*_ for 3-fold sites is about 1 order of magnitude larger than for 4-fold sites.

**Figure 3:**
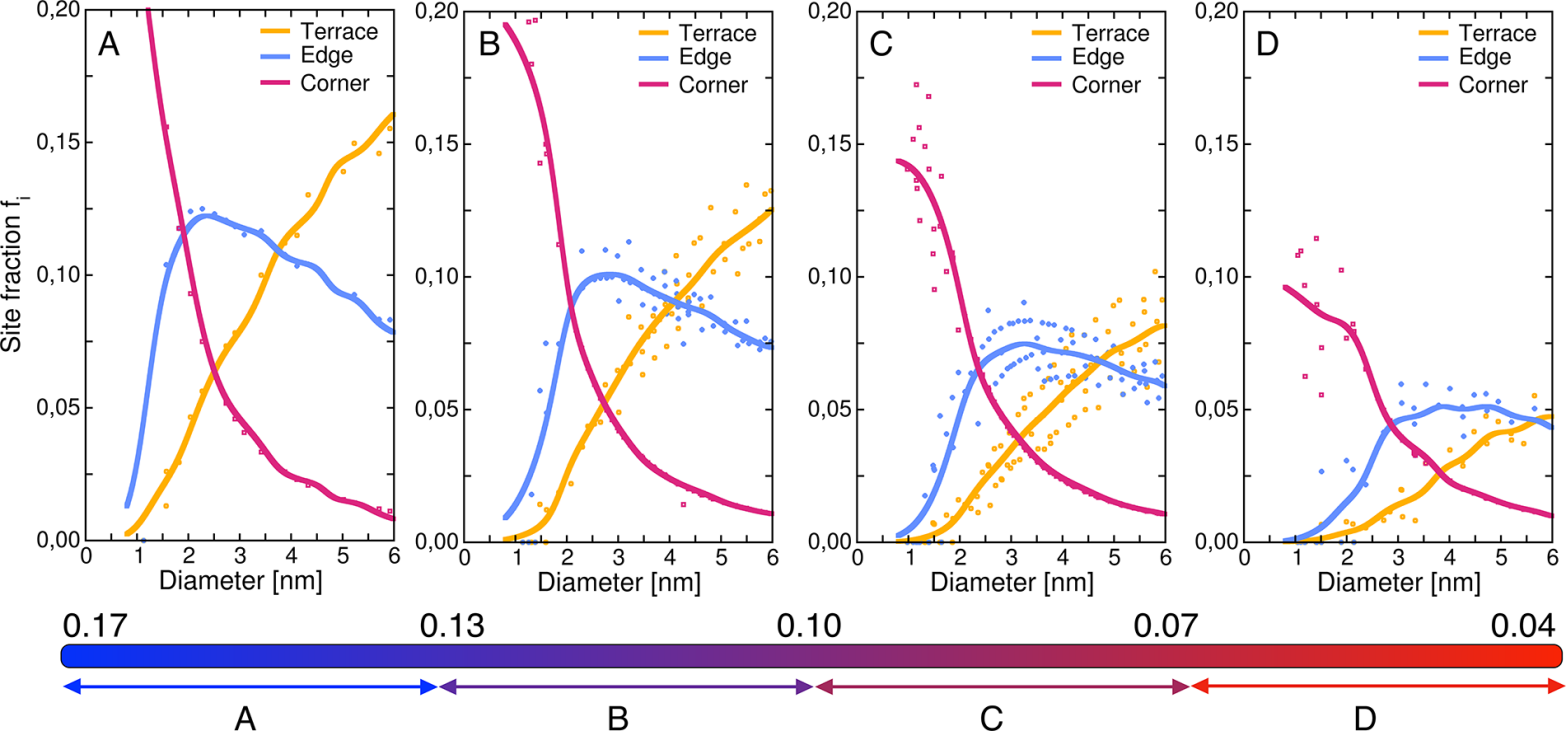
Site fraction of 4-fold sites for each nanoparticle class. Four families of nanoparticles are classified basing on their 4-fold site fraction (i.e., 4-fold sites with respect to total number of active sites): particles belonging to “A” family present a 4-fold site fraction between 13 and 17%, “B” for the range 10–13%, “C” for 7–10%, “D” for fractions of 7% or lower. The site fraction refers to the number of terrace (yellow), edge (blue), corner (red) 4-fold sites with respect to the total number of 4-fold sites of the particle. A Gaussian smoothing is adopted to interpolate the points.

As shown in Figure 2b, in the region around 2.4 nm, where the edge 4-fold sites exhibit a maximum, the active sites show significant deviation from the interpolated Gaussian smoothing. This broad distribution of sites may result from the influence of different shapes of particles coexisting at the same size. Therefore, to elucidate the role of the shapes of the nanoparticles on the site distributions, we classify the Ni NPs into four different families according to their shape. The shape is determined by the relative proportion between 3-fold and 4-fold sites. Thus, we select the fraction of 4-fold sites as a geometric descriptor for each single isolated particle. Specifically, four families are identified within a 4-fold site fraction spanning between 4 and 17% and reported in Figure 3 with a color gradient. Notably, the site distribution trend changes according to the specific family of Ni nanoparticles. Particles with a fraction of 4-fold sites between 13 and 17% exhibit a peak in the fraction of edge active sites at ∼2.2 nm. In contrast, for families with a lower fraction of 4-fold sites, the edge site distribution flattens, ultimately exhibiting a “hockey stick” behavior in families of particles with 7% or fewer 4-fold sites. Conversely, corner and terrace sites maintain a monotonic trend regardless of their respective families. As a result, the overall distribution of active sites is obtained by combining the individual site distributions of the different families.

To assess the consequences of the active site distribution at varying NP sizes, we considered the reactivity of each active site. To this aim, we adopt the microkinetic model proposed by Sterk et al.,^11^ and we consider HCO* dissociation into CH* and O* and CH_3_* formation from CH_2_* and H* as rate-determining steps over Ni(111) and Ni(100), respectively. CO* and H* are the MARI on the Ni(111) surface, while the Ni(100) surface is largely covered by the carbide C*.^20^ According to this mechanism, we derive the expression of the reaction rate over each active site, which is reported in detail in Section 1 of the SI. A site-dependent correction is applied to prevent the CO* overbinding, while H* is destabilized with 0.207 eV (20 kJ mol^–1^) over all the active sites, in agreement with previous studies.^11,47^ Given the strong binding energy between the carbide species at the 4-fold sites and the corresponding high coverage, we also account for the lateral interaction energy between C*-C* and between C* and the transition state. The latter term results in an energy shift of 0.99 eV to be added to the Gibbs free energy of the transition state, as shown in Section 2.1.2 of the SI. By means of DFT calculations, we evaluate the rate constants and the binding Gibbs free energies over 3-fold and 4-fold terrace sites using periodic slabs exposing the Ni(111) and the Ni(100) surfaces, respectively. In addition, we explicitly consider dissociation reactions that occur at the interface between two Ni(111) facets or at the interface between one Ni(111) and one Ni(100) facet. To this purpose, we remove rows of atoms from Ni(111) and Ni(100) slabs in such a way as to create atoms with coordination numbers 6 and 7, reproducing the local environment of edge and corner sites, as shown in Section 2.3 of the SI.

**Figure 4:**
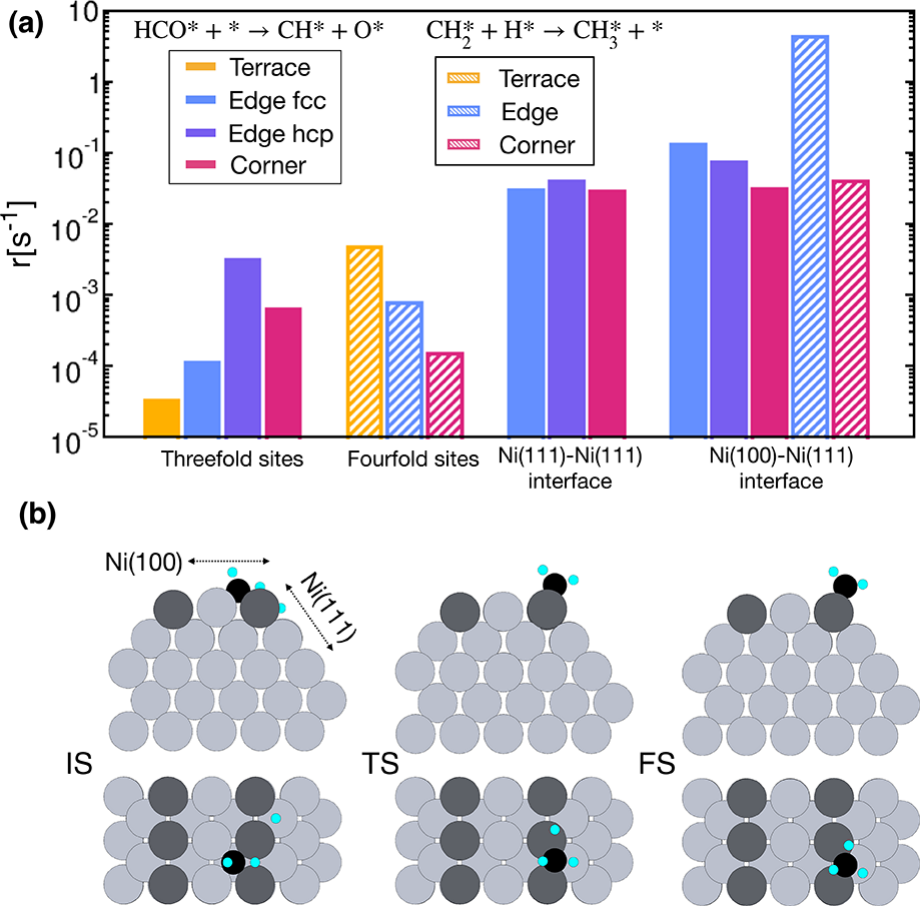
(a) Reaction rates over different Ni active sites. Uniformly filled and line-filled bars represent the rate of the HCO* dissociation and the CH_3_* formation reaction, which are taken as representative of the kinetics over 3-fold and 4-fold sites, respectively. From left to right: intraface reactions over 3-fold sites, intraface reactions over 4-fold sites, interface reactions between two (111) facets, interface reactions between one (100) and one (111) facet. (b) Side view (top) and upper view (bottom) of initial states (IS), transition states (TS), and final states (FS) for CH_3_* formation at the Ni (100)–Ni (111) interface. Gray and dark gray colors are used for Ni and edge-Ni atoms. Carbon and hydrogen are reported with black and light blue colors.

The Gibbs free energies are evaluated at a temperature of 673 K and pressure of 1 bar to reproduce the experimental conditions of Vogt et al.^8^ The geometry of the transition states is found using CI-NEB calculations, and the geometries are shown in Figures S3–S13. The binding Gibbs free energy of the MARI over each site is reported in Tables S1 and S2. The reaction rates over each site are reported in Figure 4. The CH_3_* formation at the edge of the Ni(100)–Ni(111) has by far the highest reaction rate due both to a large rate constant and to a higher availability of H* on the Ni(111) side of the Ni(111)–Ni(100) interface compared to the Ni(100), where the presence of H* is hampered by the high C* coverage. The interfacet reactions along the NP edges are thus expected to play a significant role in determining the overall activity of the NP since their reaction rate is four orders of magnitude larger than the intraface counterpart. The Ni(100)–Ni(111) interface is particularly active also for the HCO* dissociation, which here shows the highest reaction rate. At the Ni(111)–Ni(111) interface, the reaction rate for the HCO* dissociation is slightly lower than the one at the Ni(100)–Ni(111) interface, but still a couple of orders of magnitude larger than the intraface dissociation over 3-fold sites. In particular, terrace 3-fold sites have the lowest reaction rate, indicating that they are not active for the CO_2_ methanation reaction, in agreement with the findings of Lozano-Reis et al.^48^ Therefore, this analysis shows that the reaction proceeds faster at the interface between two NP facets, and therefore, to calculate the overall reaction rate over an NP, one must account for the nonlinear combination of the specific activity of two facets when put into contact. The number of active sites is explicitly determined by the atomistic procedure employed to model the metal NPs. Differently from the Wulff methodology applied by Sterk et al.,^11^ where the specific activity of undercoordinated atoms was parametrized, with the current approach, we can model the specific activity of all surface sites, without the need for parametric assignment of reaction rates.

**Figure 5:**
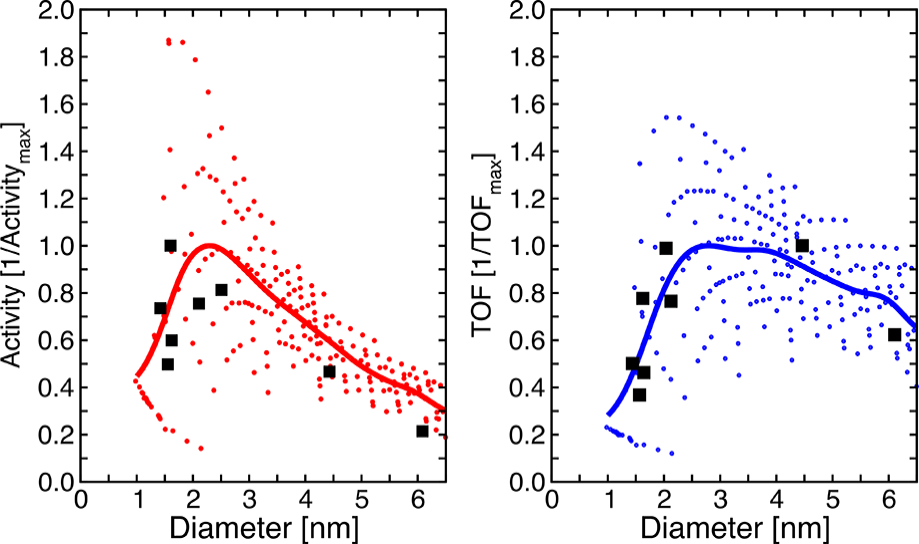
Activity (red) and turnover frequency (TOF) (blue) of the high-probability metal nanoparticles (dots) filtered with a Gaussian smearing (solid line). The experimental data of Vogt et al.^8^ are reported as black squares.

We calculate the TOFs distinctly for each active site, categorizing them according to their coordination number distribution and specific location on the NP surface (e.g., terrace, edge, or corner sites). Experimental TOF values, however, are reported with respect to the total number of surface atoms.^8^ Thus, to enable a direct and meaningful comparison with experimental data, we account for the site-specific TOFs over all surface atoms, effectively averaging the reactivity across different site types according to eqs 4 and 5. The rate over a specific site  contributes to the overall rate through its relative abundance *f*_*i*_. The dots in Figure 5 represent the activity and the TOF of the high-probability metal NPs as a function of their diameter, in comparison with the experimental values of Vogt et al.,^8^ which are reported as black squares. The experimental values represent the average activity/TOF of a set of metal NPs distributed around a given size. In order to be consistent with the experimental procedure, for each value of the size between 1 and 6 nm the data are processed with a Gaussian filter with a standard deviation of 0.25 nm. In this way, each point along the curve represents the average activity/TOF of a set of high- frequency metal NPs, whose size is normally distributed around a specific diameter. The maximum of the filtered curves and the highest value of the experimental activity/TOF are set to 1 and the other points are thus reported in terms of this value. The model reproduces qualitatively the experimental trend of the activity and the TOF as a function of NP size with excellent agreement. The TOF values exhibit a pronounced maximum for a metal NP of ∼2.3 nm in size. The contribution of each active site to the total reaction rate as a function of the diameter is shown in Figure S15. The overall trend is modulated by the edge fraction of 4-fold sites, since the active sites at the Ni(100)–Ni(111) interface are the ones with the lowest coordination numbers and the highest activity. The largest contribution to the TOF of small metal NPs (<1.25 nm) comes from interface reactions at corner sites. When the metal NPs become larger, and the number of 4-fold sites increases, the contribution of CH_3_* formation at the Ni(100)–Ni(111) interface rapidly grows, being ∼80% for the metal NPs larger than 3 nm. Our results show that only a relatively small fraction of the active sites controls the overall activity.

As expected, the overall TOF trend reported with a solid line in Figure 5 reflects the maximum in the overall edge 4-fold site distribution, as reported in Figure 2b. Indeed, these active sites are the ones with the highest catalytic activity. However, as shown in Figure 3, the distribution of the active sites differs among the families of NPs, since differently shaped NPs coexist with the same size. Therefore, this variation in the shape distribution leads us to separately analyze the TOFs of the different NPs families.

**Figure 6:**
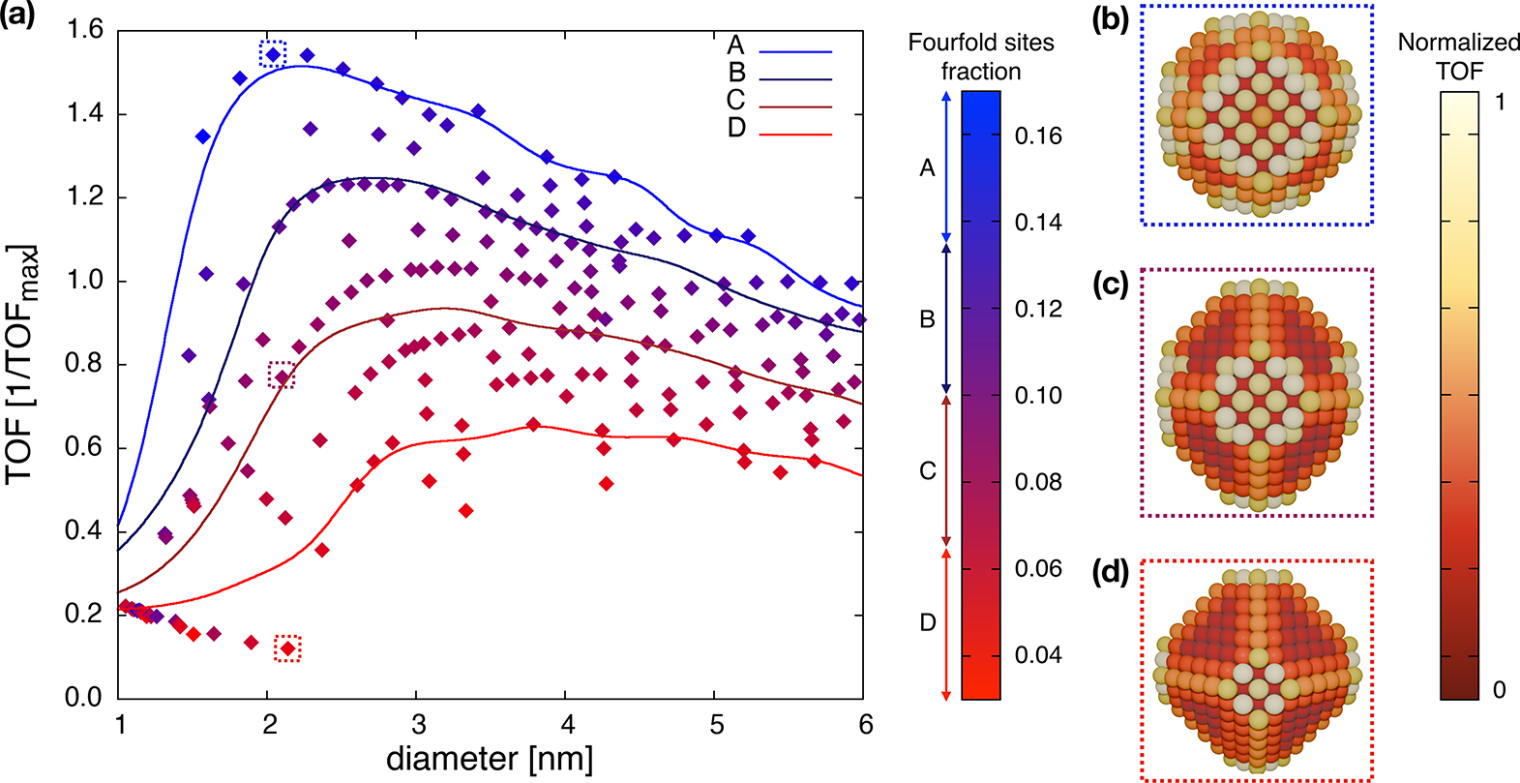
(a) Turnover frequency (TOF) of the high-probability metal nanoparticles (NPs) as a function of their diameter (diamonds), represented in terms of the 4-fold site fraction of the NP surface, according to the color bar on the right. The lines represent a Gaussian smoothing of four different groups of metal NPs (A–D), grouped according to their 4-fold sites fraction. (b–d) Crystal structure of three selected metal NPs with similar size (∼2 nm) but having high (b), intermediate (c) and low (d) TOF, with surface atoms colored according to their activity, normalized with respect to the maximum TOF.

Three Ni metal NPs with the same size (∼2 nm), but different fractions of 4-fold sites, are reported in Figure 6, as representative of the metal NPs with high (b), intermediate (c) and low (d) TOF. The surface NP sites are colored according to their intrinsic activity normalized with respect to the maximum TOF among all the exposed atoms, thus adopting a Taylor ratio approach.^4^ The different color distributions of NPs in Figure 6b–d further express that few atoms account for the total activity of NPs, and that slight changes in shape result in large variations in catalytic activity. The TOF of the subgroup of the most active metal NPs (family A) shows a well-defined maximum at ∼2 nm; see Figure 6a. Indeed, these metal NPs are those with the highest fraction of 4-fold sites, which at the Ni(100)–Ni(111) interfaces are the most active ones. The least active metal NPs (family D) have a sort of hockey stick behavior, with metal NPs bigger than 3 nm having a similar TOF. Metal NPs with an intermediate activity show a trend in-between the two extremes.

As a result of these findings, we can provide an explanation that may reconcile the different experimental trends in TOF reported as a function of the metal NP size. The origin of the structure sensitivity comes from the interplay between the size and shape effects. The experimentally observed maximum activity at ∼2.5 nm^8^ for CO_2_ methanation over a Ni/SiO_2_ catalyst can be attributed to the dominance of Ni particles with a higher fraction of 4-fold site. This condition may be particularly relevant in cases such as Ni/SiO_2_ where the metal–support interaction is not expected to be dominant.^49^ This aligns with the results of this work on isolated NPs, in which support effects are neglected. Conversely, the increase of activity with the particle size ending with an “hockey stick” behavior, such as the one observed by Visser et al.,^10^ may be attributed to a prevalence of metal NPs with a low fraction of 4-fold sites.

Therefore, the global TOF trend with NP size depends on the specific prevalent shape of the catalyst particles. In this scenario, different operating conditions, specific support materials, or different catalyst preparation methods may be crucial in dictating which is the dominant family of NPs, and consequently the global catalyst performance. Notably, our model predicts that a slight change in shape can result in large variations in catalytic activity, even when the interfacial effect of the support are neglected. This highlights the importance of interrelated size-shape effects but underscores the need to exploit the direct influence of support to account for complex interactions that may affect the variety of active sites under real working conditions in metal-oxide-supported systems. In addition, relying on generalized activity measures, such as the TOF, might cloud many of the details necessary to gain a full understanding of active sites and the related mechanistic pathways of reactivity and deactivation.^4^ This showcases the challenge of deriving structure–activity relationships in heterogeneous catalysts and calls for detailed operando characterization studies, using microscopy techniques having an atomic spatial resolution, in three dimensions.

## 4 Conclusions

This work provides new insights into the structure sensitivity of CO_2_ methanation over nickel-based catalysts by emphasizing the combined effects of the metal nanoparticle (NP) size and shape. While previous studies primarily focused on NP size, we demonstrate that the NP shape plays a crucial role in determining the overall catalyst performance. By overcoming the limitations of the Wulff construction, which only provides a fixed, equilibrium shape for metal NPs regardless of size, our approach incorporates a broader range of NP shapes that can exist under reaction conditions. This enables a more accurate representation of the active site distributions and leads to a better understanding of the origin of the observed structure sensitivity in the turnover frequency (TOF). Our results show excellent agreement with experimental observations, thereby confirming the maximum TOF experimentally observed for a metal NP of ∼2.5 nm in size. In particular, this maximum activity at a specific metal NP size is related to the TOF of the prevalent family of metal NPs, i.e., the ones with a high fraction of 4-fold sites. In contrast, different support materials or catalyst preparation methods may affect the shape of the metal NPs present within a heterogeneous catalyst sample. Consequently, this turns into a prevalence of a different shape of metal NPs, e.g., the family with a low fraction of 4-fold sites, which exhibit a “hockey stick” behavior for the TOF with the NP size. These findings pave the way for reaching a general consensus concerning the ongoing debates about the structure sensitivity in CO_2_ methanation over nickel-based catalyst materials. On a larger scale, these methods and concepts can be directly applied to other classes of structure-sensitive reactions, such as ammonia synthesis and decomposition, as well as the related Fischer–Tropsch synthesis reaction (CO hydrogenation), where structure sensitivity has also been heavily debated in the past decade.

## Supporting Information Available

Details on the reaction mechanisms, geometries of the kinetically relevant transition states, calculated turnover frequencies, and methodology adopted for the creation of metal nanoparticle ensemble including coverage effect for the most abundant reaction intermediate (PDF)

## Abbreviations

CI-NEB: climbing image nudged elastic band
DFT: density functional theory
fcc: face-centered cubic
MARI: most abundant reaction intermediate
NP: nanoparticle
PBE: Perdew–Burke–Ernzerhof
TOF: turnover frequency.
