# Supplementary material for: Deciphering Size and Shape Effects on the Structure Sensitivity of the CO2 Methanation Reaction on Nickel
Source: ACS Catal. 2025 May 2;15(10):8194–203. doi: 10.1021/acscatal.4c08084 (PMC12090210; doi:10.1021/acscatal.4c08084)
Supplement: Supplementary file 1 — cs4c08084_si_001.pdf [file cs4c08084_si_001.pdf]

Supporting information: Deciphering Size and Shape Effects on the Structure Sensitivity of the CO_2 Methanation Reaction on Nickel

Gabriele Spanò,[a,‡] Matteo Ferri,[a,‡] Raffaele Cheula,[a] Matteo Monai,[b] Bert M. Weckhuysen,[b] and Matteo Maestri*,[a]*

[a]Laboratory of Catalysis and Catalytic Processes, Dipartimento di Energia, Politecnico di Milano, Via La Masa, 34, 20156, Milano, Italy

[b]Inorganic Chemistry and Catalysis Group, Institute for Circular and Sustainable Chemistry, Utrecht University, Universiteitsweg 99, 3584 CG Utrecht, The Netherlands

‡*These authors equally contributed to this work*

* b.m.weckhuysen@uu.nl
*matteo.maestri@polimi.it

1 KINETIC SCHEME OF THE CO_2 METHANATION REACTION 1

1.1 SCHEME A (THREEFOLD SITES) 1
1.2 SCHEME B (FOURFOLD SITES) 2
1.3 RATE EXPRESSION FOR INTRA-FACET REACTIONS OVER NI(111) 3
1.4 RATE EXPRESSION FOR INTRA-FACET REACTIONS OVER NI(100) 6
1.5 RATE EXPRESSION FOR THE INTERFACE REACTIONS 7
1.5.1 RATE EXPRESSION FOR THE (100)-(111) INTERFACE REACTION 8
1.5.2 RATE EXPRESSION FOR THE (111)-(100) INTERFACE REACTION 8
1.5.3 RATE EXPRESSION FOR THE (111)-(111) INTERFACE REACTION 9

2 DFT CALCULATIONS OF THE REACTION RATES 10

2.1 CALCULATION OF THE GIBBS FREE ENERGY 10
2.1.1 MOST ABUNDANT REACTION INTERMEDIATES (MARI) 11
2.1.2 TRANSITION STATES 15
2.2 REACTION RATE EVALUATION 17
2.3 GEOMETRY OF THE RATE DETERMINING STEP OVER THE ACTIVE SITES 20

3 CALCULATION OF ACTIVITY AND TURNOVER FREQUENCIES 31

3.1 ACTIVE SITE CONTRIBUTIONS TO THE TOTAL REACTION RATE 33
3.2 ABSOLUTE VALUE OF TURNOVER FREQUENCY 34
3.2.1 TOF AT DIFFERENT CONVERSION VALUES 34
3.2.2 ENERGETIC PENALTY TO H^* DUE TO LATERAL INTERACTIONS 35
3.2.3 TOF AT DIFFERENT CH_3*-C* LATERAL INTERACTIONS 36

4 SIMPLIFIED KINETIC SCHEME OF THE CO_2 METHANATION REACTION 37

5 IDENTIFICATION OF NANOPARTICLES WITH THE HIGHEST PROBABILITY OF OCCURRENCE 39

5.1 ENSEMBLE OF NI NANOPARTICLES 39
5.1.1 NANOPARTICLES DIAMETER 40
5.2 FORMATION ENERGY MODEL 41
5.2.1 BENCHMARK OF THE ENERGY MODEL 43
5.3 BOLTZMANN STATISTICS: AN EXAMPLE OF APPLICATION 44
5.4 COMPOSITION OF THE HIGH-PROBABILITY NANOPARTICLES 46

6 ACTIVE SITES DISTRIBUTION IN PRESENCE OF CO^* 47

7 TOF OF THE WULFF-CONSTRUCTED NI NANOPARTICLES 60

REFERENCES 61

1 Kinetic scheme of the CO_2 methanation reaction

We model the CO_2 methanation reaction over Ni nanoparticles using a site-dependent kinetic scheme. Two different schemes are employed for threefold and fourfold sites, each one of them having a different rate determining step (RDS), under the assumption that the other steps are pseudo-equilibrated. The RDS has been selected from the work of Sterk et al.[S1] as the elementary step with the highest degree of rate control over the (111) and (100) surfaces, representative of threefold and fourfold sites, respectively. The dissociation of the HCO^* intermediate species into CH^* and O^* has been reported to be the RDS over the (111) surface, in agreement with previous works,[S2,S3] while the CH^*_3 formation from CH_2^* and H^* has the highest degree of rate control over the (100) surface.[S1] In the following we label as "RA" and "RB" the elementary steps of the kinetic scheme over the threefold and fourfold sites, respectively. The two schemes are interconnected when the cross-talking of the facets is taken into account.

1.1 Scheme A (threefold sites)

RA1) $CO_2^{(g)} + * \Leftrightarrow CO_2^*$ $\quad K_{A1}^{eq} = \frac{\vartheta_{CO_2*}}{P_{CO_2}\vartheta_*} = \exp\left(-\frac{G^0_{CO_2*} - G^0_{CO_2^{(g)}}}{k_B T}\right)$

RA2) $H_2^{(g)} + 2* \Leftrightarrow 2H^*$ $\quad K_{A2}^{eq} = \frac{\vartheta^2_{H*}}{P_{H_2}\vartheta^2_*} = \exp\left(-\frac{2G^0_{H*} - G^0_{H_2^{(g)}}}{k_B T}\right)$

RA3) $CO_2^* + * \Leftrightarrow CO^* + O^*$ $\quad K_{A3}^{eq} = \frac{\vartheta_{CO*}\vartheta_{O*}}{\vartheta_{CO_2*}\vartheta^*} = \exp\left(-\frac{G^0_{CO*} + G^0_{O*} - G^0_{CO_2^*}}{k_B T}\right)$

RA4) $CO^* + H^* \Leftrightarrow HCO^* + *$ $\quad K_{A4}^{eq} = \frac{\vartheta_{HCO*}\vartheta_*}{\vartheta_{CO*}\vartheta_{H*}} = \exp\left(-\frac{G^0_{HCO*} - G^0_{CO*} - G^0_{H*}}{k_B T}\right)$

RA5) $HCO^* + * \rightarrow CH^* + O^*$ $\quad \vec{r} = \vec{k}_{A5}\vartheta_{HCO*}\vartheta_*$

RA6) $CH^* + 3H^* \Leftrightarrow CH_4^{(g)} + 4*$ $\quad K_{A6}^{eq} = \frac{P_{CH_4}\vartheta^4_*}{\vartheta_{CH*}\vartheta^3_{H*}} = \exp\left(-\frac{G^0_{CH_4^{(g)}} - G^0_{CH*} - 3G^0_{H*}}{k_B T}\right)$

RA7) $O^* + 2H^* \Leftrightarrow H_2O^* + 2*$ $\quad K_{A7}^{eq} = \frac{\vartheta_{H_2O*}\vartheta^2_*}{\vartheta_{O*}\vartheta^2_{H*}} = \exp\left(-\frac{G^0_{H_2O*} - G^0_{O*} - 2G^0_{H*}}{k_B T}\right)$

RA8) $H_2O^* \Leftrightarrow H_2O^{(g)} + *$

$$K^{eq}_{A8} = \frac{P_{H_2O}\vartheta_*}{\vartheta_{H_2O*}} = \exp\left(-\frac{G^0_{H_2O(g)} - G^0_{H_2O*}}{k_B T}\right)$$

RA9) $CO^* \Leftrightarrow CO^{(g)} + *$

$$K^{eq}_{A9} = \frac{P_{CO}\vartheta_*}{\vartheta_{CO*}} = \exp\left(-\frac{G^0_{CO(g)} - G^0_{CO*}}{k_B T}\right)$$

ϑ_{A*} represents the coverage of the adsorbed species A $*$, ϑ_* is the fraction of free sites and P_A is the partial pressure of the gas-phase species. $G^0_{A(g)}$ and $G^0_{A^*}$ represent the Gibbs free energy of the gas phase and the adsorbed molecule, respectively.

1.2 Scheme B (fourfold sites)

RB1) $CO_2^{(g)} + * \Leftrightarrow CO_2^*$

$$K^{eq}_{B1} = \frac{\vartheta_{CO_2*}}{P_{CO_2}\vartheta_*} = \exp\left(-\frac{G^0_{CO_2*} - G^0_{CO_2^{(g)}}}{k_B T}\right)$$

RB2) $H_2^{(g)} + 2* \Leftrightarrow 2H^*$

$$K^{eq}_{B2} = \frac{\vartheta^2_{H*}}{P_{H_2}\vartheta^2_*} = \exp\left(-\frac{2G^0_{H*} - G^0_{H_2^{(g)}}}{k_B T}\right)$$

RB3) $CO_2^* + * \Leftrightarrow CO^* + O^*$

$$K^{eq}_{B3} = \frac{\vartheta_{CO*}\vartheta_{O*}}{\vartheta_{CO_2*}\vartheta^*} = \exp\left(-\frac{G^0_{CO*} + G^0_{O*} - G^0_{CO_2^*}}{k_B T}\right)$$

RB4) $CO^* + H^* \Leftrightarrow HCO^* + *$

$$K^{eq}_{B4} = \frac{\vartheta_{HCO*}\vartheta_*}{\vartheta_{CO*}\vartheta_{H*}} = \exp\left(-\frac{G^0_{HCO*} - G^0_{CO*} - G^0_{H*}}{k_B T}\right)$$

RB5) $COH^* + * \Leftrightarrow C^* + OH^*$

$$K^{eq}_{B5} = \frac{\vartheta_{C*}\vartheta_{OH*}}{\vartheta_{COH*}\vartheta^*} = \exp\left(-\frac{G^0_{C*} + G^0_{OH*} - G^0_{COH*}}{k_B T}\right)$$

RB6) $C^* + 2H^* \Leftrightarrow CH_2^* + 2*$

$$K^{eq}_{B6} = \frac{\vartheta_{CH_2*}\vartheta^2_*}{\vartheta_{C*}\vartheta^2_{H*}} = \exp\left(-\frac{G^0_{CH2^*} - G^0_{C*} - 2G^0_{H*}}{k_B T}\right)$$

RB7) $CH_2^* + H^* \rightarrow CH_3^* + *$

$$\vec{r} = \vec{k}_{B7}\vartheta_{CH_2*}\vartheta_{H^*}$$

RB8) $CH_3^* + H^* \Leftrightarrow CH_4^{(g)} + 2*$

$$K^{eq}_{B8} = \frac{P_{CH_4}\vartheta^2_*}{\vartheta_{CH_3*}\vartheta_{H*}} = \exp\left(-\frac{G^0_{CH_4^{(g)}} - G^0_{CH_3*} - G^0_{H*}}{k_B T}\right)$$

RB9) $OH^* + H^* \Leftrightarrow H_2O^* + *$ $$K_{B9}^{eq} = \frac{\vartheta_{H_2O*}\vartheta_*}{\vartheta_{OH*}\vartheta_{H*}} = \exp\left(-\frac{G_{H_2O*}^0 - G_{OH*}^0 - G_{H*}^0}{k_BT}\right)$$

RB10) $H_2O^* \Leftrightarrow H_2O^{(g)} + *$ $$K_{B10}^{eq} = \frac{P_{H_2O}\vartheta_*}{\vartheta_{H_2O*}} = \exp\left(-\frac{G_{H_2O(g)}^0 - G_{H_2O*}^0}{k_BT}\right)$$

RB11) $O^* + H^* \Leftrightarrow OH^* + *$ $$K_{B11}^{eq} = \frac{\vartheta_{OH*}\vartheta_*}{\vartheta_{O*}\vartheta_{H*}} = \exp\left(-\frac{G_{OH*}^0 - G_{O*}^0 - G_{H*}^0}{k_BT}\right)$$

1.3 Rate expression for intra-facet reactions over Ni(111)

The (111) facets of the nanoparticle are characterized by the presence of terrace, edge and corner threefold sites. The reaction rate of intra-face reactions over these sites is uniquely determined by the rate of the elementary step *RA*5, which is the RDS of the *A* scheme:

$$\vec{r} = \vec{k}_{A5}\theta_{HCO*}\theta_* \quad . \tag{1}$$

The three terms in Eq.(1) are computed separately as follows:

- The rate constant $\vec{k}_{A5}$ is computed in the framework of the transition state theory:[S4]

$$\vec{k}_{A5} = \frac{k_BT}{h}\exp\left(-\frac{\Delta G_{A5}^{act,0}}{k_BT}\right) = \frac{k_BT}{h}\exp\left(-\frac{G_{TS,A5}^0 - G_{HCO^*}^0}{k_BT}\right) \quad . \tag{2}$$

In the above equation, k_B is the Boltzmann constant and h the Planck constant.

- The coverage term ϑ_{HCO*} is determined from RA4):

$$\vartheta_{HCO*} = K_{A4}^{eq}\frac{\vartheta_{CO*}\vartheta_{H*}}{\vartheta_*} \quad . \tag{3}$$

To find an expression for ϑ_{HCO*}, we first extract ϑ_{CO*} and ϑ_{H*} from RA3) and RA2):

$$\vartheta_{CO*} = K_{A3}^{eq}\frac{\vartheta_{CO_2*}\vartheta_*}{\vartheta_{O*}} \text{ , } \vartheta_{H*} = \left(K_{A2}^{eq}P_{H_2}\right)^{\frac{1}{2}}\vartheta_* \quad , \tag{4}$$

then we obtained ϑ_{CO*} from RA1), RA2), RA7) and RA8) using some algebra:

$$\vartheta_{CO*} = K_{A1}^{eq}K_{A2}^{eq}K_{A3}^{eq}K_{A7}^{eq}K_{A8}^{eq}\frac{P_{CO_2}P_{H_2}}{P_{H_2O}}\vartheta_* \quad . \tag{5}$$

Substituting Eq.(4) and (5) into Eq.(3) we obtain the following expression for ϑ_{HCO*}:

$$\vartheta_{HCO*} = K_{A1}^{eq}\left(K_{A2}^{eq}\right)^{\frac{3}{2}}K_{A3}^{eq}K_{A7}^{eq}K_{A8}^{eq}\frac{P_{CO_2}P_{H_2}^{\frac{3}{2}}}{P_{H_2O}}\vartheta_* \quad . \tag{6}$$

$$\vartheta_{HCO*} = \exp\left(-\frac{G_{HCO*}^0 + G_{H_2O(g)}^0 - G_{CO_2^{(g)}}^0 - \frac{3}{2}G_{H_2^{(g)}}^0}{k_B T}\right)\frac{P_{CO_2}P_{H_2}^{\frac{3}{2}}}{P_{H_2O}}\vartheta_* \quad . \tag{7}$$

Eq.(7) shows that the coverage term ϑ_{HCO*} depends only on the Gibbs free energy of HCO* over the site, computed with respect to the reactants.

- The fraction of free sites ϑ^* is determined imposing the site balance condition of all the reaction intermediates:

$$\vartheta_* + \vartheta_{CO_2*} + \vartheta_{H*} + \vartheta_{CO*} + \vartheta_{O*} + \vartheta_{HCO*} + \vartheta_{CH*} + \vartheta_{H_2O*} = 1 \quad . \tag{8}$$

However, only the coverage of CO* and H* was reported to be significant, thus we can neglect the contribution of all the other reaction intermediates:

$$\vartheta_* + \vartheta_{CO*} + \vartheta_{H*} \simeq 1 \quad . \tag{9}$$

The value of ϑ_{CO*} is determined from RA9), assuming equilibrium between the CO* on the surface and CO in gas phase at the pressure P_{CO}, while ϑ_{H*} is taken from RA2):

$$\vartheta_{CO*} = \frac{P_{CO}\vartheta_*}{K_{A9}^{eq}} \quad \vartheta_{H*} = \left(K_{A2}^{eq}P_{H_2}\right)^{\frac{1}{2}}\vartheta_* \quad . \tag{10}$$

The expression for the fraction of free sites is then:

$$\vartheta_* = \frac{1}{1 + \frac{P_{CO}}{K_{A9}^{eq}} + \left(K_{A2}^{eq}P_{H_2}\right)^{\frac{1}{2}}} = \frac{1}{1 + P_{CO}K_{CO}^{ads} + K_H^{ads}P_{H_2}^{\frac{1}{2}}} \quad , \tag{11}$$

where we have defined K_{CO}^{ads} and K_H^{ads} as the equilibrium constants of CO* and H* adsorption from the gas phase, respectively:

$$K_{CO}^{ads} = \left(K_{A9}^{eq}\right)^{-1} = \exp\left(-\frac{G_{CO*}^0 - G_{CO(g)}}{k_B T}\right) \tag{12}$$

$$K_H^{ads} = \left(K_{A2}^{eq}\right)^{\frac{1}{2}} = \exp\left(-\frac{G_{H*}^0 - \frac{1}{2}G_{H_2^{(g)}}}{k_B T}\right) \tag{13}$$

The expression for ϑ_{CO*} in Eq.(10) must be equivalent to the one Eq.(5):

$$\frac{P_{CO}}{K_{A9}^{eq}} = K_{A1}^{eq}K_{A2}^{eq}K_{A3}^{eq}K_{A7}^{eq}K_{A8}^{eq}\frac{P_{CO_2}P_{H_2}}{P_{H_2O}} \quad . \tag{14}$$

Defining K_{CO} as the product of the equilibrium constants in Eq.(14):

$$K_{CO} = K_1^{eq}K_2^{eq}K_3^{eq}K_7^{eq}K_8^{eq} = \exp\left(-\frac{G_{CO*}^0 + G_{H_2O(g)}^0 - G_{CO_2^{(g)}}^0 - G_{H_2^{(g)}}^0}{k_B T}\right) \quad , \tag{15}$$

the fraction of free sites can be equivalently expressed as:

$$\vartheta_* = \frac{1}{1 + K_{CO}\frac{P_{CO_2}P_{H_2}}{P_{H_2O}} + K_H^{ads}P_{H_2}^{\frac{1}{2}}} \quad . \tag{16}$$

K_{CO} represents the equilibrium constant of CO^* adsorption computed with respect to the reactants of the CO_2 methanation reaction and it is related to K_{CO}^{ads} through the relation:

$$K_{CO} = K_{CO}^{ads}K_{revWGS} \quad , \tag{17}$$

where K_{revWGS} is the equilibrium constant of the revWGS reaction:

$$K_{revWGS} = \exp\left(-\frac{G_{CO^{(g)}}^0 + G_{H_2O^{(g)}}^0 - G_{CO_2^{(g)}}^0 - G_{H_2^{(g)}}^0}{k_B T}\right) \quad , \tag{18}$$

The final expression for the reaction rate over threefold sites is obtained by substituting Eq.(2), Eq.(7) and Eq.(16) into Eq.(1):

$$\vec{r} = k_{app}\frac{P_{CO_2}P_{H_2}^{\frac{3}{2}}}{P_{H_2O}}\left(\frac{1}{1 + K_{CO}\frac{P_{CO_2}P_{H_2}}{P_{H_2O}} + K_H^{ads}P_{H_2}^{\frac{1}{2}}}\right)^2 \tag{19}$$

$$k_{app} = \frac{k_B T}{h}\exp\left(-\frac{G_{TS,A5}^0 + G_{H_2O(g)}^0 - G_{CO_2^{(g)}}^0 - \frac{3}{2}G_{H_2^{(g)}}^0}{k_B T}\right) \tag{20}$$

$$K_{CO} = \exp\left(-\frac{G_{CO*}^0 + G_{H_2O^{(g)}}^0 - G_{CO_2^{(g)}}^0 - G_{H_2^{(s)}}^0}{k_B T}\right) \tag{21}$$

$$K_H^{ads} = \left(K_{A2}^{eq}\right)^{\frac{1}{2}} = \exp\left(-\frac{G_{H*}^0 - \frac{1}{2}G_{H_2^{(g)}}}{k_B T}\right) \tag{22}$$

The Gibbs free energy of the CO^* and H^* adsorption and the Gibbs free energy of transition state (TS) of RA5 elementary step are thus the only descriptors of the activity of each active site. These factors may differ among terrace, edge and corner sites, giving rise to different reaction rates for the different sites.

1.4 Rate expression for intra-facet reactions over Ni(100)

The (100) facets of the nanoparticle are characterized by the presence of terrace, edge and corner fourfold sites. The reaction rate of intra-face reactions over these sites is uniquely determined by the rate of the elementary step RB7, which is the RDS of the B scheme:

$$\vec{r} = \vec{k}_{B7} \vartheta_{CH_2*} \vartheta_{H^*} \quad . \tag{23}$$

The three terms in Eq.(23) are computed separately as follows:

- The rate constant $\vec{k}_{B7}$ is computed within the transition state theory:

$$\vec{k}_{B7} = \frac{k_B T}{h} \exp\left(-\frac{\Delta G_{B7}^{act,0}}{k_B T}\right) = \frac{k_B T}{h} \exp\left(-\frac{G_{TS,B7}^0 - G_{CH_2^*}^0 - G_{H^*}^0}{k_B T}\right) \quad . \tag{24}$$

- The coverage term ϑ_{CH_2*} is determined from RB6:

$$\vartheta_{CH_2*} = K_{B6}^{eq} \frac{\vartheta_{C*} \vartheta_{H*}^2}{\vartheta_*^2} \quad . \tag{25}$$

  and the final expression is obtained with some algebra:

$$\vartheta_{CH_2*} = K_{B1}^{eq} \left(K_{B2}^{eq}\right)^3 K_{B3}^{eq} K_{B4}^{eq} K_{B5}^{eq} K_{B6}^{eq} \left(K_{B9}^{eq}\right)^2 \left(K_{B10}^{eq}\right)^2 K_{B11}^{eq} \frac{P_{CO_2} {P_{H_2}}^3}{{P_{H_2O}}^2} \vartheta_* \tag{26}$$

$$\vartheta_{CH_2*} = \exp\left(-\frac{G_{CH_2*}^0 + 2G_{H_2O(g)}^0 - G_{CO_2^{(g)}}^0 - 3G_{H_2^{(g)}}^0}{k_B T}\right) \frac{P_{CO_2} {P_{H_2}}^3}{{P_{H_2O}}^2} \vartheta_* \quad . \tag{27}$$

- The coverage term ϑ_{H*} is computed from RB2:

$$\vartheta_{H*} = \left(K_{B2}^{eq} P_{H_2}\right)^{\frac{1}{2}} \vartheta_* \quad . \tag{28}$$

- The fraction of free sites ϑ^* which appears in the above equations is determined imposing the site balance condition of all the reaction intermediates. However, according to the work of Sterk et al.,[S1] C^* is the most abundant reaction intermediate over the (100) surface, hence the fraction of the other intermediates is assumed to be negligible:

$$\vartheta * + \vartheta_{C*} \simeq 1 \quad . \tag{29}$$

ϑ_{C*} is obtained from the equilibrium constants of the scheme B as:

$$\vartheta_{C*} = K_{B1}^{eq}\left(K_{B2}^{eq}\right)^2 K_{B3}^{eq}K_{B4}^{eq}K_{B5}^{eq}\left(K_{B9}^{eq}\right)^2\left(K_{B10}^{eq}\right)^2 K_{A11}^{eq}\frac{P_{CO_2}{P_{H_2}}^2}{{P_{H_2O}}^2}\vartheta_* \tag{30}$$

$$\vartheta_{C*} = \exp\left(-\frac{G_{C*}^0 + 2G_{H_2O(g)}^0 - G_{CO_2^{(g)}}^0 - 2G_{H_2^{(g)}}^0}{k_BT}\right)\frac{P_{CO_2}{P_{H_2}}^2}{{P_{H_2O}}^2}\vartheta_* \quad . \tag{31}$$

The fraction of free sites becomes:

$$\vartheta_* = \frac{1}{1 + K_C\frac{P_{CO_2}{P_{H_2}}^2}{{P_{H_2O}}^2}} \quad . \tag{32}$$

The expression for the reaction rate over the fourfold sites is eventually obtained by inserting Eq.(24), Eq.(27), Eq.(28), and Eq.(32) into Eq.(23):

$$\vec{r} = k_{app}\frac{P_{CO_2}{P_{H_2}}^{\frac{7}{2}}}{{P_{H_2O}}^2}\left(\frac{1}{1 + K_{CO}\frac{P_{CO_2}{P_{H_2}}^2}{{P_{H_2O}}^2}}\right)^2 \tag{34}$$

$$k_{app} = \frac{k_BT}{h}\exp\left(-\frac{G_{TS,B7}^0 + 2G_{H_2O(g)}^0 - G_{CO_2^{(g)}}^0 - \frac{7}{2}G_{H_2^{(g)}}^0}{k_BT}\right) \tag{35}$$

$$K_C = \exp\left(-\frac{G_{C*}^0 + 2G_{H_2O^{(g)}}^0 - G_{CO_2^{(g)}}^0 - 2G_{H_2^{(s)}}^0}{k_BT}\right) \tag{36}$$

The Gibbs free energy of the C^* adsorbate and the Gibbs free energy of transition state (TS) of RB7 elementary step are thus the only descriptors of the activity of each active site. These factors may differ among terrace, edge and corner sites, giving rise to different reaction rates for the different sites.

1.5 Rate expression for the interface reactions

We take explicitly into account the occurrence of interface reactions across consecutive facet at the edges of catalyst nanoparticles. Therefore, the two kinetic schemes presented above become interconnected. At the interface between the (100) and the (111) facets, two interface processes of the kinetically relevant steps may occur. First, the CH_3^* formation from a CH_2^* lying at the edge/corner of the (100) facet and a H^* lying on the edge/corner of the (111) facet.

Second, the dissociation of the HCO^* intermediate may occur across the interface, hence depending on the fraction of free fourfold sites. The dissociation of the HCO^* intermediate may also occur across the interface between two (111) facets. In the following we analyze the rate expression in the three different cases.

1.5.1 Rate expression for the (100)-(111) interface reaction

The rate expression of the CH_3^* formation from a CH^* lying at the edge/corner of the (100) facet and a H^* lying on the edge/corner of the (111) facet can be expressed as:

$$\vec{r} = \vec{k}_{B7}\vartheta_{CH_2*}^{(100)}\vartheta_{H^*}^{(111)} \tag{37}$$

- The rate constant $\vec{k}_{B7}$ is computed within the transition state theory, as in Eq.(24).
- The coverage term $\vartheta_{CH_2*}^{(100)}$ is taken from Eq.(27) and depends on the fraction of free fourfold sites over the (100) surfaces, cfr Eq.(32).
- The coverage term $\vartheta_{H^*}^{(111)}$ is taken from Eq.(10) and Eq.(22) and depends on the fraction of free threefold sites over the (111) surfaces, cfr Eq.(16).

The expression of the reaction rate is then:

$$\vec{r}= k_{app}\frac{P_{CO_2}P_{H_2}^{\frac{7}{2}}}{P_{H_2O}^{2}}\left(\left(\frac{1}{1+K_C^{(100)}\frac{P_{CO_2}P_{H_2}^{2}}{P_{H_2O}^{2}}}\right)\left(\frac{1}{1+K_{CO}^{(111)}\frac{P_{CO_2}P_{H_2}}{P_{H_2O}}+K_H^{ads(111)}P_{H_2}^{\frac{1}{2}}}\right)\right) \tag{38}$$

$$k_{app} = \frac{k_BT}{h}\exp\left(-\frac{G_{TS,B7}^0+2G_{H_2O(g)}^0-G_{CO_2^{(g)}}^0-\frac{7}{2}G_{H_2^{(g)}}^0}{k_BT}\right) \tag{39}$$

1.5.2 Rate expression for the (111)-(100) interface reaction

The dissociation of the HCO^* intermediate may occur across the (100)-(111) interface, the reaction rate being:

$$\vec{r} = \vec{k}_{A5}\vartheta_{HCO*}^{(111)}\vartheta_{*}^{(100)} \quad . \tag{40}$$

- The rate constant $\vec{k}_{A5}$ is computed in the framework of the transition state theory as in Eq.(24).
- The coverage term $\vartheta_{HCO*}^{(111)}$ is taken from Eq.(7), and depends on the fraction of free threefold sites over the (111) surfaces, cfr Eq.(16).
- The fraction of free fourfold sites $\vartheta_{*}^{(100)}$ is taken from Eq.(32).

The reaction rate assumes the following form:

$$\vec{r} = k_{app}\frac{P_{CO_2}P_{H_2}^{\frac{3}{2}}}{P_{H_2O}}\left(\frac{1}{1+K_{CO}^{(100)}\frac{P_{CO_2}P_{H_2}}{P_{H_2O}}+K_{H}^{ads(111)}P_{H_2}^{\frac{1}{2}}}\right)\left(\frac{1}{1+K_{C}^{(100)}\frac{P_{CO_2}P_{H_2}^{2}}{P_{H_2O}^{2}}}\right)) \quad (41)$$

$$k_{app} = \frac{k_B T}{h}\exp\left(-\frac{G_{TS,A5}^{0} + G_{H_2O(g)}^{0} - G_{CO_2^{(g)}}^{0} - \frac{3}{2}G_{H_2^{(g)}}^{0}}{k_B T}\right) \quad (42)$$

1.5.3 Rate expression for the (111)-(111) interface reaction

The HCO^* dissociation reaction may also occur at the interface between two (111) surfaces:

$$\vec{r} = \vec{k}_{A5}\vartheta_{HCO*}^{(111-a)}\vartheta_{*}^{(111-b)} \quad . \quad (43)$$

The expression for both $\vartheta_{HCO*}^{(111-a)}$ and $\vartheta_{*}^{(111-b)}$ are taken from scheme A, hence the expression for the reaction rate is the same as in Eq.(19).

2 DFT calculations of the reaction rates

2.1 Calculation of the Gibbs free energy

The Gibbs free energy energy of the adsorbed molecules and the TS is calculated as:

$$G^0_{A*} = G^0_{slab+A*} - G^0_{slab} \quad . \tag{44}$$

The first term in the right-hand side of each equation represents the Gibbs free energy of the system composed by the slab and the adsorbates:

$$G^0_{slab+A*} = E_{slab+A*} + ZPE_{A*} + U^{vib}_{A*}(T) - TS_{A*}(T) \quad , \tag{45}$$

where $E_{slab+A*}$ is the DFT energy of the unit cell containing the slab and the adsorbates, ZPE is the zero-point energy of the adsorbates, $U^{vib}_{A*}(T)$ and $S_{A*}(T)$ is the vibrational contribution to the internal energy and entropy at the temperature *T*, respectively, which are computed from the vibrational energies ε_i:

$$U^{vib}_{A*}(T) = \sum_i \frac{\varepsilon_i}{\exp\left(\frac{\varepsilon_i}{k_B T}\right)} \quad , \tag{46}$$

$$S_{A*}(T) = k_B \sum_i \left[\frac{\varepsilon_i}{k_B T\left(\exp\left(\frac{\varepsilon_i}{k_B T}\right) - 1\right)} - \ln\left(1 - \exp\left(-\frac{\varepsilon_i}{k_B T}\right)\right)\right] \quad , \tag{47}$$

The vibrational contribution due to the slab is neglected both in $G^0_{slab+A*}(T)$ and in $G^0_{slab}(T)$, with the latter approximated with the DFT energy E_{slab}.

The Gibbs free energy of the gas phase molecules are computed as:

$$G^0_{CO_2^{(g)}}(T) = E_{CO_2^{(mol)}} + ZPE_{CO_2} + \Delta G_{CO_2}(T) \quad , \tag{48}$$

$$G^0_{H_2^{(g)}}(T) = E_{H_2^{(mol)}} + ZPE_{H_2} + \Delta G_{H_2}(T) \quad , \tag{49}$$

$$G^0_{H_2O^{(g)}}(T) = E_{H_2O^{(mol)}} + ZPE_{H_2O} + \Delta G_{H_2O}(T) \quad , \tag{50}$$

where $E_{CO_2}^{(mol)}$, $E_{H_2}^{(mol)}$ and $E_{H_2}^{(mol)}$ are the DFT energies of the isolated molecules, while the zero-point energies and the free energies variations are taken from the NIST-Janaf database at the temperature of 673 K and standard pressure of 1 bar.[S5]

2.1.1 Most Abundant Reaction Intermediates (MARI)

The Gibbs free energy of adsorption of the most abundant reaction intermediates and the value of the corresponding equilibrium constants computed according Eq.(21),(22) and (36) are reported in Table S1 and Table S3. We adopt the following shorthand notation:

- $\widetilde{G^0_{CO*}} = G^0_{CO*} + G^0_{H_2O^{(g)}} - G^0_{CO_2^{(g)}} - G^0_{H_2^{(g)}}$
- $\widetilde{G^0_{H*}} = G^0_{H*} - \frac{1}{2} G^0_{H_2^{(g)}}$
- $\widetilde{G^0_{C*}} = G^0_{C*} + 2G^0_{H_2O^{(g)}} - G^0_{CO_2^{(g)}} - 2G^0_{H_2^{(g)}}$

In the specific case of CO, the Gibbs free energy of the adsorbed molecule G^0_{CO*} was corrected *a-posteriori* using a site-dependent energy correction proposed by Mason et al.:[S6 e]

$$\Delta E(\nu_{CO}) = 0.45 - \frac{0.4}{2100 - 1600}(\nu_{CO} - 1600) \quad , \tag{51}$$

where ν_{CO} is the vibrational frequency of the C-O stretching, expressed in cm^{-1}. Threefold fcc sites have a C-O stretching frequency between 1725 and 1760 cm^{-1}, while fourfold hollow sites between 1623 and 1630 cm^{-1}. The corresponding corrections are 0.32−0.35 eV and 0.42−0.43 eV, respectively.

Table S1: Gibbs free binding energy, equilibrium constant of the CO^* adsorption over three-fold sites and corresponding coverage for two different values of the conversion.

**Site**	$\widetilde{G^0_{CO*}}$ **[eV]**	K_{CO*} **[1/s]**	$\vartheta_{CO*}[\chi = 3\%]$	$\vartheta_{CO*}[\chi = 10\%]$
Three-fold Terrace	0.12	0.1165	0.42	0.15
Three-fold Edge fcc	0.12	0.1165	0.31	0.09
Three-fold Edge hcp	0.15	0.0691	0.41	0.15
Three-fold Corner	0.14	0.0964	0.38	0.13

Table S2: Gibbs free binding energy, equilibrium constant of the CO* adsorption over three-fold sites and corresponding coverage for two different values of the conversion.

Site	$\widetilde{G^0_{CO*}}$[eV]	K_{CO*}[1/s]	$\vartheta_{H*}[\chi = 3\%]$	$\vartheta_{H*}[\chi = 10\%]$
Three-fold Terrace	-0.09	4.627	0.04	0.07
Three-fold Edge fcc	-0.11	6.380	0.03	0.04
Three-fold Edge hcp	-0.05	2.465	0.06	0.09
Three-fold Corner	-0.06	2.795	0.03	0.04

Table S3: Gibbs free energy and corresponding equilibrium constant of the most abundant reaction intermediates over the fourfold sites.

Site	$\widetilde{G^0_{C*}}$[eV]	K_C[1/s]	ϑ_{C*}
Four-fold Terrace	-0.50	5727.27	0.999
Four-fold Edge	-0.33	300.54	0.999
Four-fold Corner	-0.21	36.77	0.999

The high value of the equilibrium constant leads to an unphysical coverage of C* larger than 99%. However, this value does not take into account the lateral interaction between the adsorbates, which needs to be computed. We evaluated the lateral interaction over Ni(100) surfaces and the value has been later adopted to compute the equilibrium C* coverage over terrace, edge and corner sites.

The lateral interaction energy is obtained from DFT calculations of supercells containing CO* at different coverage (0.25 ML, 0.5 ML and ! ML) as:

$$E^{lat}(\vartheta_{C*}) = E_{C*}(\vartheta_{C*}) - E_{C*}(\vartheta^0) \quad , \tag{52}$$

where $E_{C*}(\vartheta^0)$ is the CO* coverage at which the effect of lateral interaction is null and $E_{C*}(\vartheta_{C*})$ the average energy of CO* molecules at the coverage (ϑ_{C*}):

$$E_{C*}(\vartheta_{C*}) = \frac{E_{slab+N_{C*}} - E_{slab} - N_{C*}E_C^{ref}}{N_{C*}} \quad . \tag{53}$$

In the previuos equation E_C^{ref} is the reference energy of C:

$$E_C^{ref} = E_{CO_2^{(g)}} - 2E_{H_2O^{(g)}} + 2E_{H_2^{(g)}} \quad . \tag{54}$$

The lateral interaction energy as a function of the coverage is reported in Figure S1. The C* coverage is computed as the coverage that minimizes the average Gibbs free energy of C* adsorption:

$$G^{b}_{C*}(\vartheta_{C*}) = \vartheta_{C*}\left(G^{b}_{C*}(\vartheta^{0}) + E^{lat}(\vartheta_{C*})\right) \quad . \tag{55}$$

The average Gibbs free energy of adsorption of terrace sites is reported in Figure S2, and a summary in Table S4.

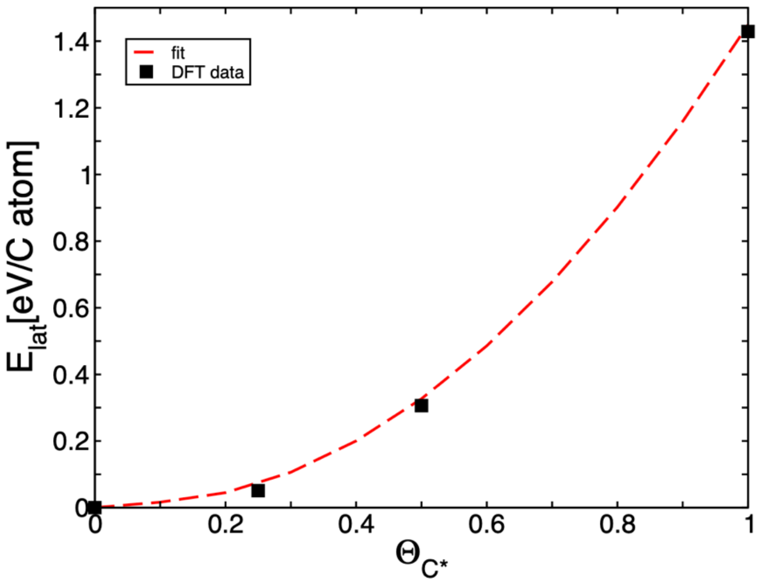

Figure S1: Lateral interaction energy between the carbide adsorbates C* as a function of the coverage.

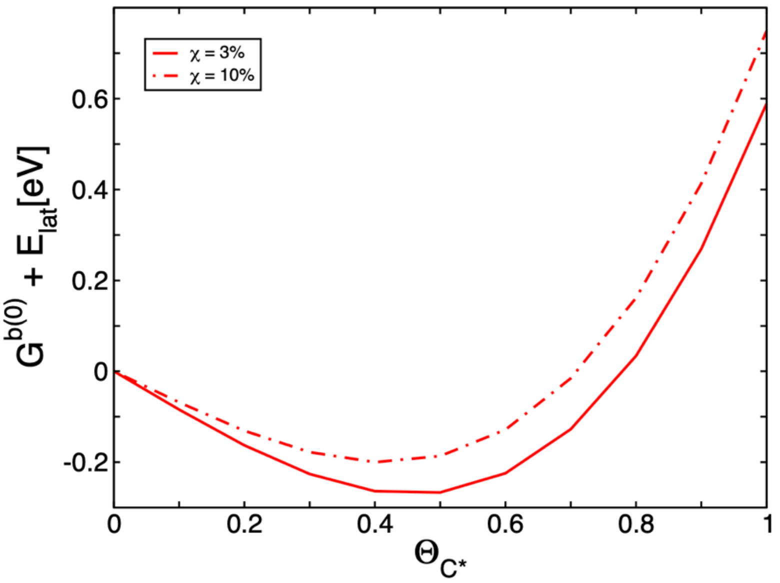

Figure S2: Calculation of the C* coverage over fourfold terrace sites for two different values of the conversion.

Table S4: Gibbs free energy and corresponding equilibrium constant of the most abundant reaction intermediates over the fourfold sites.

Site	$\vartheta_{H*}[\chi = 3\%]$	$\vartheta_{H*}[\chi = 10\%]$
Four-fold Terrace	0.46	0.41
Four-fold Edge	0.41	0.37
Four-fold Corner	0.38	0.33

2.1.2 Transition States

The Gibbs free energy of the transition state of the HCO* dissociation and the CH_3* formation reactions are reported in Table S5 and Table S6 respectively, with the corresponding k_{app}. The following shorthand notation are used:

- $\widetilde{G^0_{TS,A5}} = G^0_{TS,5} + G^0_{H_2O^{(g)}} - G^0_{CO_2^{(g)}} - \frac{3}{2} G^0_{H_2^{(g)}}$
- $\widetilde{G^0_{TS,B7}} = G^0_{TS,B7} + 2G^0_{H_2O^{(g)}} - G^0_{CO_2^{(g)}} - \frac{7}{2} G^0_{H_2^{(g)}}$

Given the high carbibe coverage over fourfold sites, we accounted for the lateral interaction between C* and the transition state. Since the geometry of the transition state is more similar to the products than the reactants (*late transition state*), we evaluated the lateral interaction between C* and CH_3* as representative of the transition state. We constructed a 2×2 Ni(100) cell with 0.5 ML of C* adsorbed on top, and we computed the lateral interaction as:

$$E^{lat}_{TS} = E_{slab+0.5ML\ C*+CH_3*} - E_{slab+0.5ML\ C*} - E_{CH_3*} \quad . \tag{56}$$

We found a value of 0.99 eV, which has been added to the Gibbs free energy of the TS over each fourfold site.

Table S5: Gibbs free energy of the transition state of the HCO* dissociation reaction evaluated over the different threefold active sites.

**Site**	**Reaction**	$\widetilde{G^0_{TS,A5}}$ **[eV]**	k_{app} **[1/s]**
Three-fold Terrace	Intra-face	2.22	3.54e-4
Three-fold Edge fcc	Intra-face	2.15	1.04e-3
Three-fold Edge fcc	Interface (111)-(111)	1.83	2.77e-1
Three-fold Edge fcc	Interface (100)-(111)	1.74	1.53
Three-fold Edge hcp	Intra-face	1.93	2.85e-2
Three-fold Edge hcp	Interface (111)-(111)	1.81	3.72e-1
Three-fold Edge hcp	Interface (100)-(111)	1.77	7.17e-1
Three-fold Corner	Intra-face	2.00	1.58e-2
Three-fold Corner	Interface (111)-(111)	1.83	2.57e-1
Three-fold Corner	Interface (100)-(111)	1.82	3.05e-1

Table S6: Gibbs free energy of the transition state of the CH_3* formation and corresponding apparent rate constants on the different fourfold active sites.

Site	Reaction	$\widetilde{G^0_{TS,A5}}$[eV]	k_{app}[1/s]
Four-fold Terrace	Intra-face	2.07	4.58e-3
Four-fold Edge	Intra-face	2.18	6.41e-4
Four-fold Edge	Interface (100)-(111)	1.69	3.38
Four-fold Corner	Intra-face	2.28	1.20e-4
Four-fold Corner	Interface (100)-(111)	1.96	2.70e-2

2.2 Reaction rate evaluation

The overall reaction rate $\vec{R}$ over a Ni metal nanoparticle is computed as:

$$\vec{R}\left[\frac{1}{s}\right] = \sum_{i} N_i \vec{r}_i \quad . \tag{57}$$

In Eq.(57), $\vec{r}_i$ represents the reaction rate of the faster dissociation process occurring the i-th active site, computed according to Eq.(19).

The partial pressures entering Eq.(19) are computed as follows. We define λ as the extent of the reaction, representing the progress made towards the equilibrium. The variation of the number of moles of the i-th species during the reaction is given by the product between the corresponding stoichiometric coefficient ν_i and the variation of the extent of the reaction:

$$dn_i = \nu_i d\lambda \quad . \tag{58}$$

Integrating Eq.(58) between two different states we obtain:

$$n_i^{(2)} = n_i^{(1)} + \nu_i \lambda \quad . \tag{59}$$

In the scheme defined by the steps from R1) to R9) the CO_2 methanation reaction, $CO_2 + 4\ H_2 \rightarrow CH_4 + 2\ H_2O$, competes with the reverse water gas shift (revWGS) reaction, $CO_2 + H_2 \Leftrightarrow CO + H_2O$, which is assumed to be equilibrated.
The variation of the number of moles between a generic final state and the initial state due to the methanation and the water gas shift reaction are:

$$n_{CO_2} = n_{CO_2}^{in} - \lambda_1 - \lambda_2 \tag{60}$$

$$n_{H_2} = n_{H_2}^{in} - 4\lambda_1 - \lambda_2 \tag{61}$$

$$n_{CH_4} = \lambda_1 \tag{62}$$

$$n_{H_2O} = 2\lambda_1 + \lambda_2 \tag{63}$$

$$n_{CO} = \lambda_2 \quad . \tag{64}$$

where λ_1 and λ_2 represent the reaction extent of the methanation and the revWGS reaction, respectively. The total number of moles in the final state is then given by:

$$n_{tot} = n_{CO_2}^{in} + n_{H_2}^{in} - 2\lambda_1 + n_{inert}^{in} = n_{tot}^{in} - 2\lambda_1 \quad , \tag{65}$$

where n_{inert}^{in} and $n_{tot} = n_{CO_2}^{in} + n_{H_2}^{in} + n_{inert}^{in}$ represent the total number of moles of the inert gas and the total number of moles in the inlet mixture, respectively. Considering the CO_2 conversion and the CH_4 selectivity, it is possible to find an expression for λ_1 and λ_2:

$$\lambda_1 = n_{CO_2}^{in}\chi\xi \quad , \tag{66}$$

$$\lambda_2 = n_{CO_2}^{in}\chi(1-\xi) \quad , \tag{67}$$

and substituting into Eq.(60)-(65):

$$n_{CO_2} = n_{CO_2}^{in}(1-\chi) \tag{68}$$

$$n_{H_2} = n_{H_2}^{in} - n_{CO_2}^{in}\chi(3\xi+1) \tag{69}$$

$$n_{CH_4} = n_{CO_2}^{in}\chi\xi \tag{70}$$

$$n_{H_2O} = n_{CO_2}^{in}\chi(1+\xi) \tag{71}$$

$$n_{CO} = n_{CO_2}^{in}\chi(1-\xi) \tag{72}$$

$$n_{tot} = n_{tot}^{in} - 2n_{CO_2}^{in}\chi\xi \tag{73}$$

The partial pressure of the species is defined as $P_i = P\, n_i/n_{tot}$, where P is the total pressure:

$$P_{CO_2} = P\frac{n_{CO_2}^{in}(1-\chi)}{n_{tot}^{in} - 2n_{CO_2}^{in}\chi\xi} \tag{74}$$

$$P_{H_2} = P\frac{n_{H_2}^{in} - n_{CO_2}^{in}\chi(3\xi+1)}{n_{tot}^{in} - 2n_{CO_2}^{in}\chi\xi} \tag{75}$$

$$P_{CH_4} = P\frac{n_{CO_2}^{in}\chi\xi}{n_{tot}^{in} - 2n_{CO_2}^{in}\chi\xi} \tag{76}$$

$$P_{H_2O} = P\frac{n_{CO_2}^{in}\chi(1-\xi)}{n_{tot}^{in} - 2n_{CO_2}^{in}\chi\xi} \tag{77}$$

$$P_{CO} = P\frac{n_{CO_2}^{in}\chi(1-\xi)}{n_{tot}^{in} - 2n_{CO_2}^{in}\chi\xi} \tag{78}$$

which can be expressed in terms of molar fractions as:

$$P_{CO_2} = P\frac{x_{CO_2}^{in}(1-\chi)}{1 - 2x_{CO_2}^{in}\chi\xi} \tag{79}$$

$$P_{H_2} = P\frac{x_{H_2}^{in} - x_{CO_2}^{in}\chi(3\xi + 1)}{1 - 2x_{CO_2}^{in}\chi\xi} \quad (80)$$

$$P_{CH_4} = P\frac{x_{CO_2}^{in}\chi\xi}{1 - 2x_{CO_2}^{in}\chi\xi} \quad (81)$$

$$P_{H_2O} = P\frac{x_{CO_2}^{in}\chi(1 - \xi)}{1 - 2x_{CO_2}^{in}\chi\xi} \quad (82)$$

$$P_{CO} = P\frac{x_{CO_2}^{in}\chi(1 - \xi)}{1 - 2x_{CO_2}^{in}\chi\xi} \quad (83)$$

In the experiments of Vogt et al.,[S7] for a total pressure of 1 bar, the inlet CO_2:H_2:inert ratio is 1:4:5, corresponding to molar fractions $x_{CO_2}^{in} = 0.1$ and $x_{H_2}^{in} = 0.4$. We report in Table S7 the partial pressure of the gaseous species for different values of the conversion and selectivity.

Table S7: Partial pressures of the gaseous species for the different values of conversion.

χ	ξ	P_{CO_2}[bar]	P_{H_2}[bar]	P_{CH_4}[bar]	P_{H_2O}[bar]	P_{CO}[bar]
3%	20%	0.0971	0.3957	0.0006	0.0036	0.0024
3%	50%	0.0973	0.3937	0.0015	0.0045	0.0015
3%	90%	0.0975	0.3910	0.0027	0.0057	0.0003
20%	20%	0.0806	0.3710	0.0040	0.0242	0.0161
20%	50%	0.0816	0.3571	0.0102	0.0306	0.0102
20%	90%	0.0830	0.3382	0.0187	0.0394	0.0021
50%	20%	0.0510	0.3265	0.0102	0.0612	0.0408
50%	50%	0.0526	0.2895	0.0263	0.0789	0.0263
50%	90%	0.0549	0.2363	0.0495	0.1044	0.0055
80%	20%	0.0207	0.2810	0.0165	0.0992	0.0661
80%	50%	0.0217	0.2174	0.0435	0.1304	0.0435
80%	90%	0.0234	0.1215	0.0841	0.1776	0.0093

2.3 Geometry of the rate determining step over the active sites

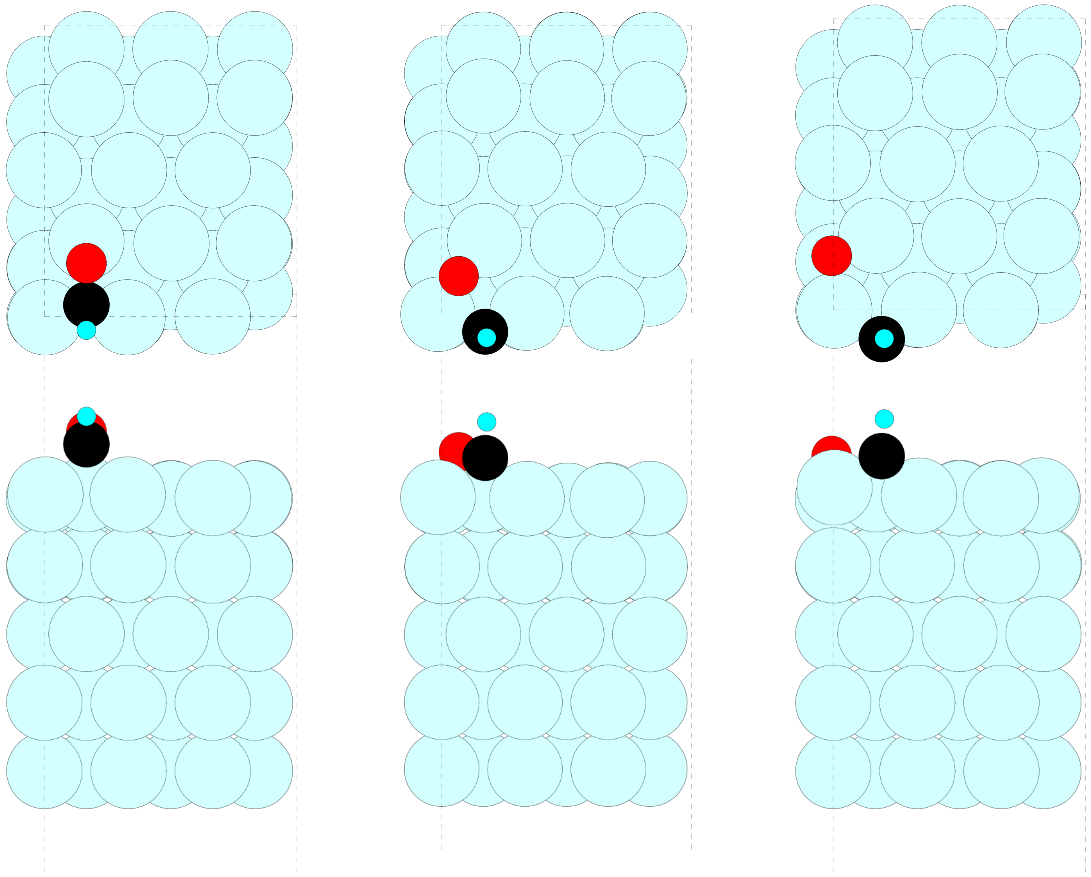

Figure S3: From left to right: top and side view of the crystal structure of the initial, transition and final state of the intra-face HCO* dissociation reaction over threefold terrace sites.

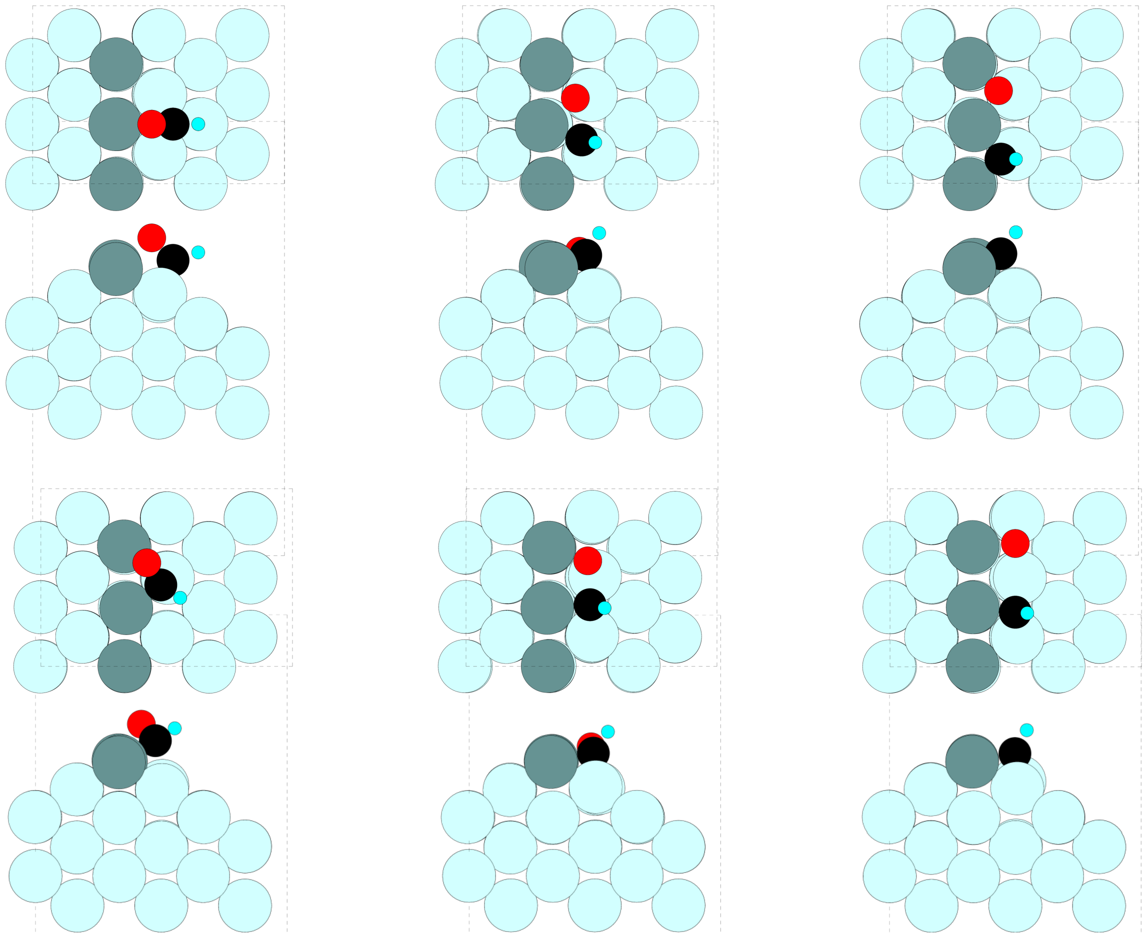

Figure S4: From left to right: top and side view of the crystal structure of the initial, transition and final state of the intra-face HCO* dissociation reaction over threefold Edge hcp (top panel) and Edge fcc (bottom panel) sites. Edge atoms are depicted with darker spheres.

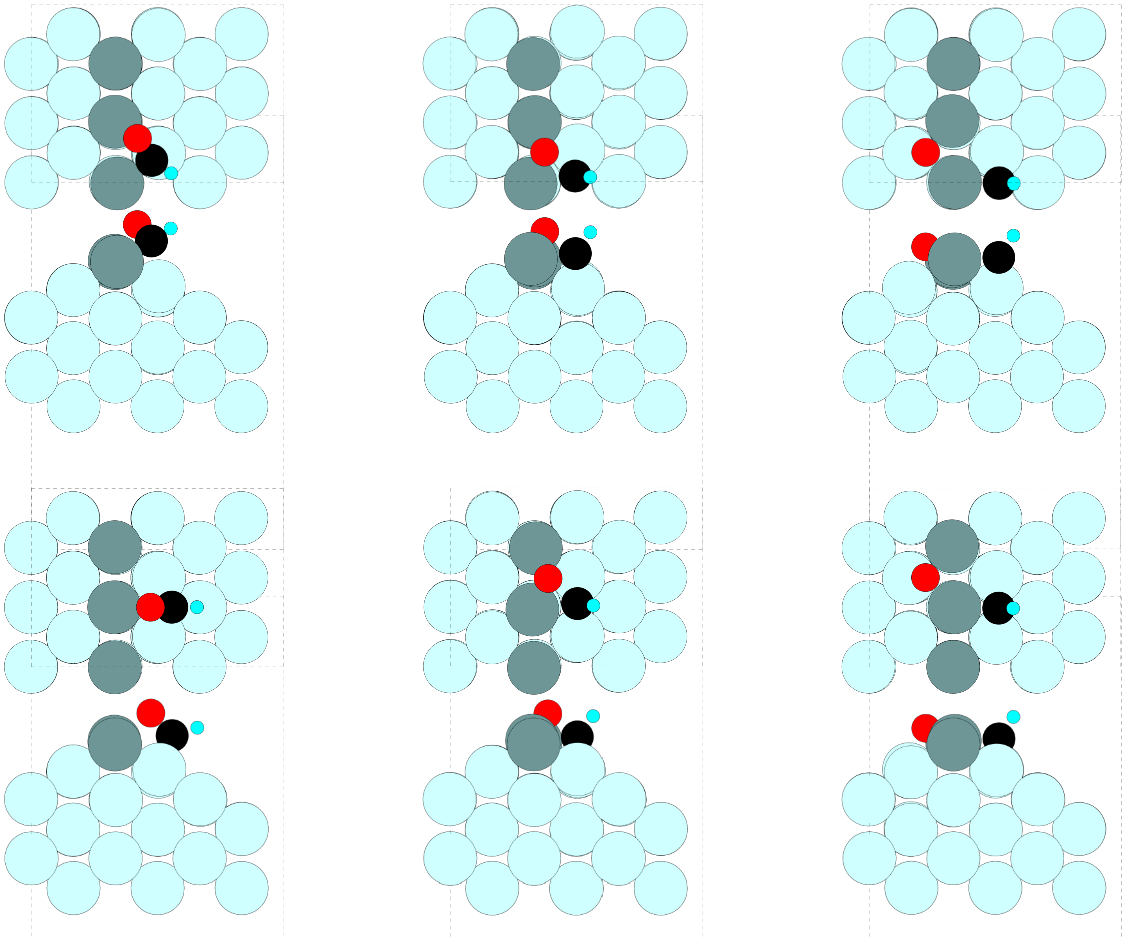

Figure S5: From left to right: top and side view of the crystal structure of the initial, transition and final state of the interface HCO* dissociation reaction over threefold Edge fcc (top panel) and Edge hcp (bottom panel) sites at the Ni(111)-Ni(111) interface. Edge atoms are depicted with darker spheres.

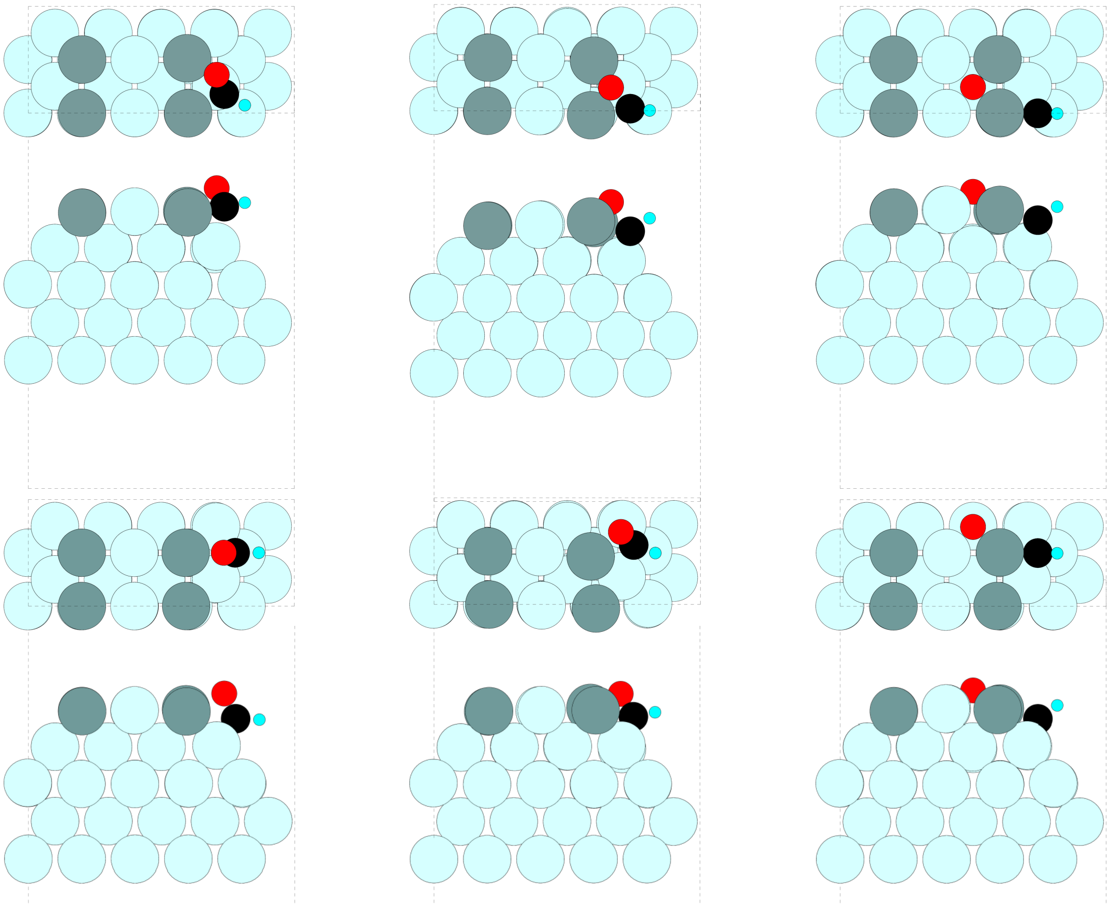

Figure S6: From left to right: top and side view of the crystal structure of the initial, transition and final state of the interface HCO* dissociation reaction over threefold Edge fcc (top panel) and Edge hcp (bottom panel) sites at the Ni(111)-Ni(100) interface. Edge atoms are depicted with darker spheres.

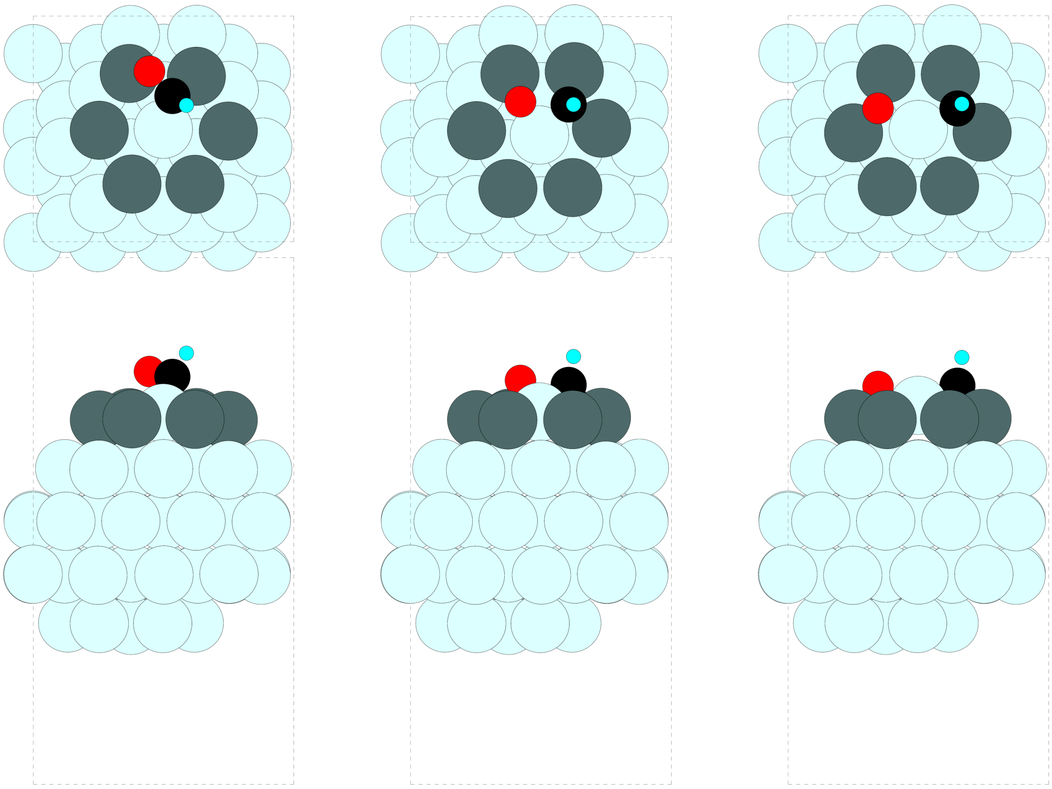

Figure S7: From left to right: top and side view of the crystal structure of the initial, transition and final state of the intra-face HCO* dissociation over threefold corner sites. Corner atoms are depicted with darker spheres.

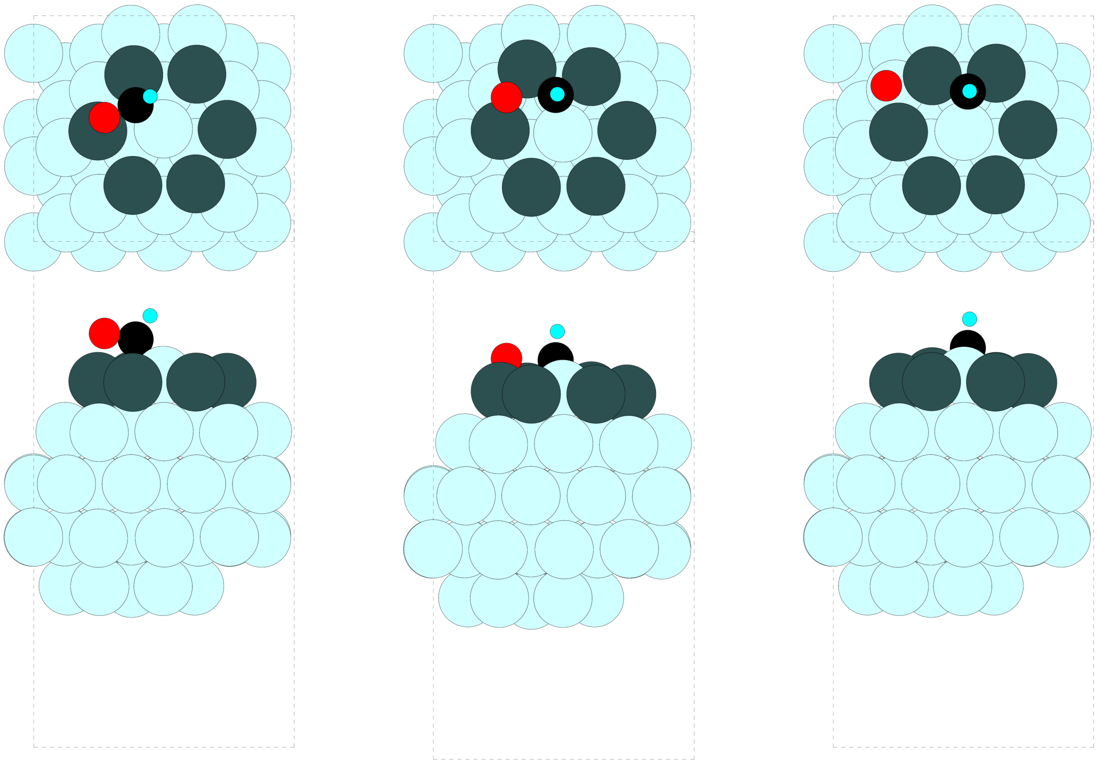

Figure S8: From left to right: top and side view of the crystal structure of the initial, transition and final state of the interface HCO* dissociation reaction over threefold corner sites at the Ni(111)-Ni(111) interface.

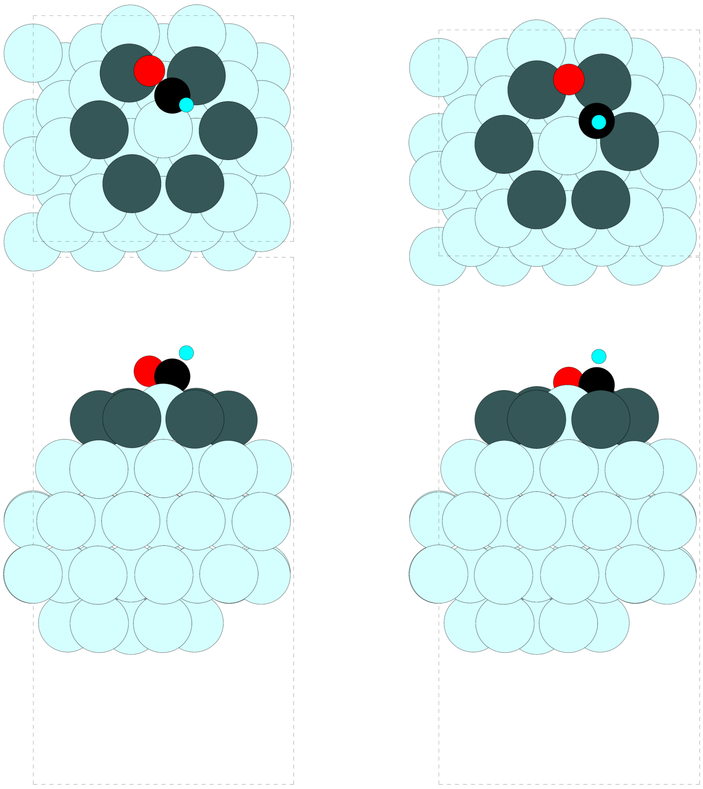

Figure S9: From left to right: top and side view of the crystal structure of the initial, transition and final state of the interface HCO* dissociation reaction over threefold corner sites at the Ni(111)-Ni(100) interface.

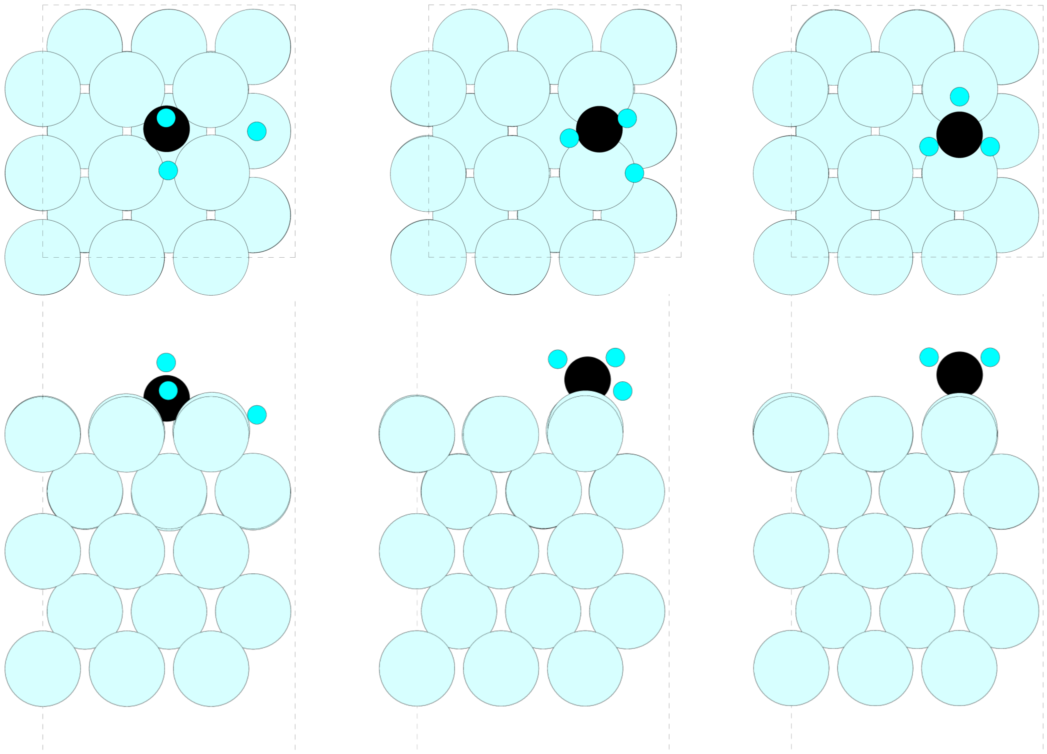

Figure S10: From left to right: top and side view of the crystal structure of the initial, transition and final state of the intra-face CH_3* dissociation reaction over fourfold terrace sites.

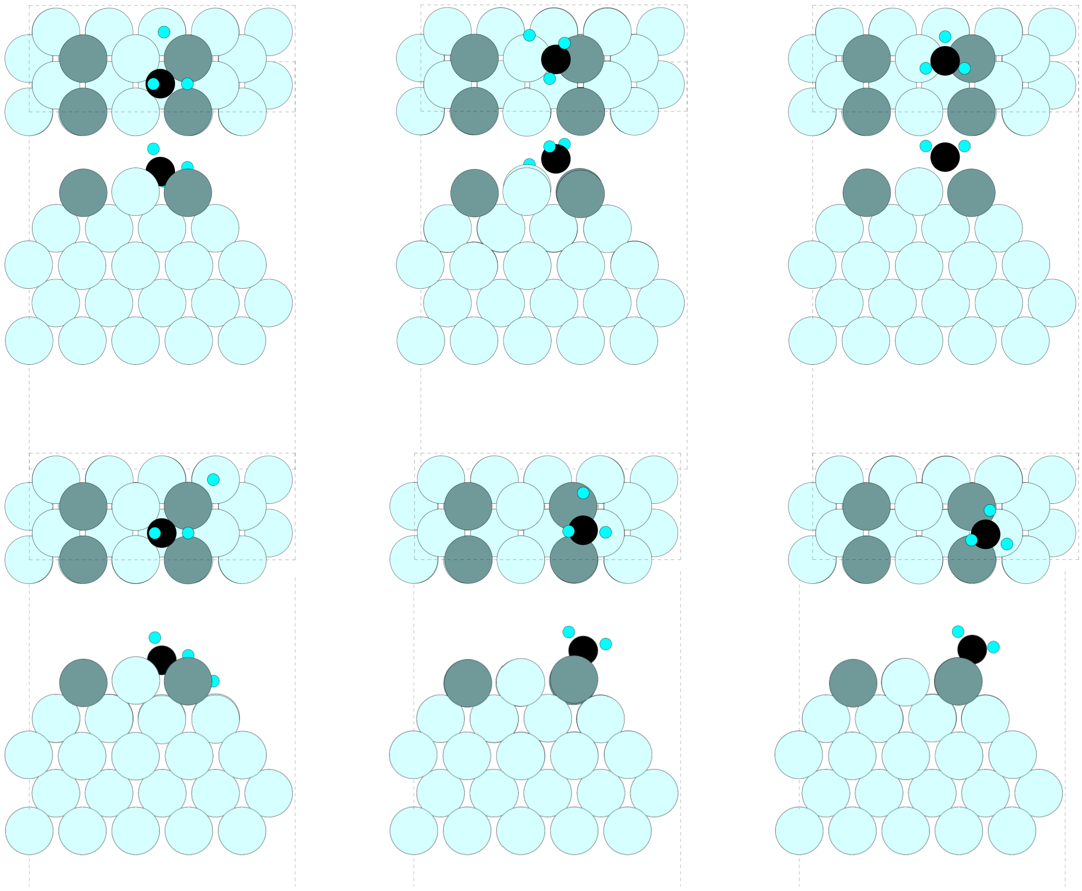

Figure S11: From left to right: top and side view of the crystal structure of the initial, transition and final state of the intraface (top panel) and interface (bottom panel) CH_3* dissociation reaction over fourfold edge sites. Edge sites are depicted using larger spheres.

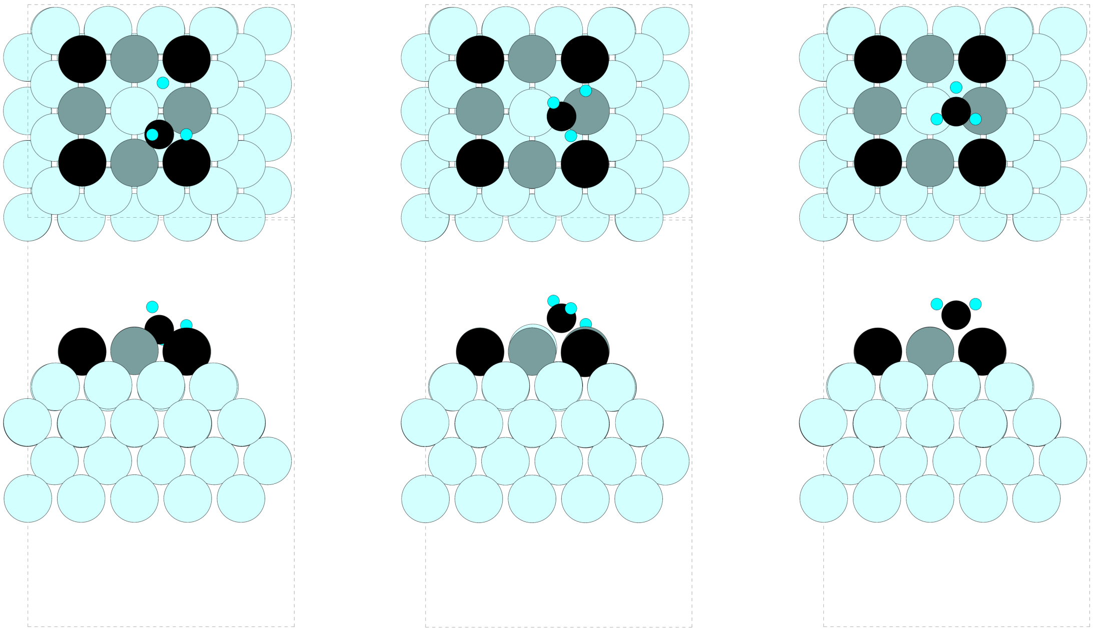

Figure S12: From left to right: top and side view of the crystal structure of the initial, transition and final state of the intraface CH_3* dissociation reaction over fourfold corner sites. Edge and corner sites are depicted using grey and black spheres, respectively.

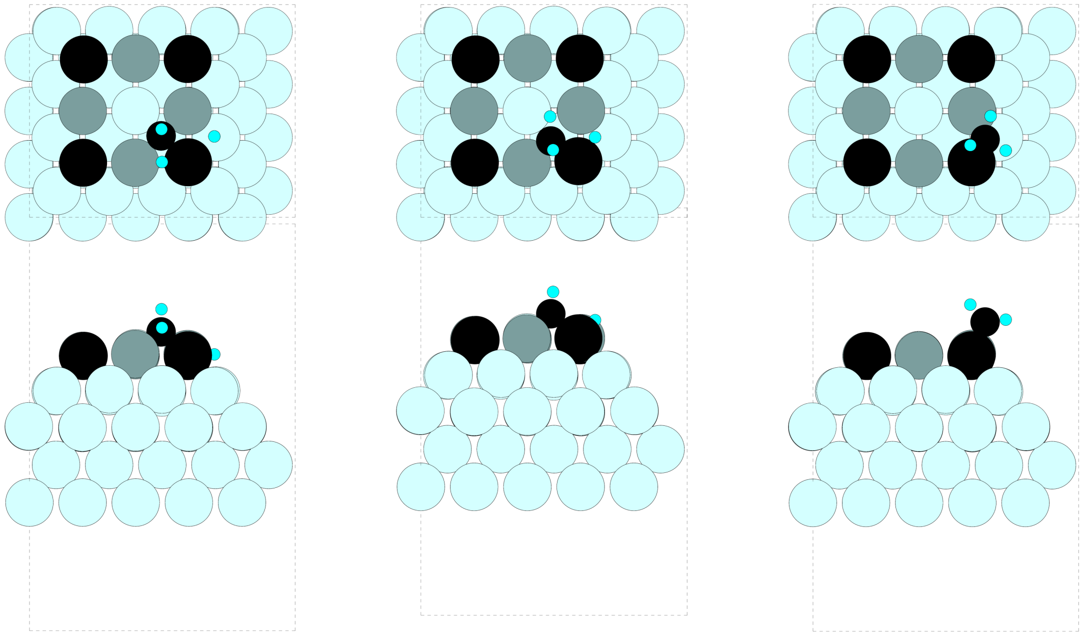

Figure S13: From left to right: top and side view of the crystal structure of the initial, transition and final state of the interface CH_3* dissociation reaction over fourfold corner sites at the Ni(100)-Ni(111) interface. Edge and corner sites are depicted using grey and black spheres, respectively.

3 Calculation of activity and turnover frequencies

The activity and the TOF over a single nanoparticle are computed from the overall reaction rate $\vec{R}$ as:

$$A\left[\frac{\mathrm{mol}}{\mathrm{s\,g}}\right] = \frac{\vec{R}}{m_{cat}}\frac{1}{N_{avo}} = \frac{\vec{R}}{N_{atoms}MW_{Ni}} \quad , \tag{84}$$

$$\mathrm{TOF}\left[\frac{1}{\mathrm{s}\,N_{atoms,surf}}\right] = \frac{\vec{R}}{N_{atoms,surf}} \quad . \tag{85}$$

where MW_{Ni} = 58.69 g/mol is the molecular weight of Ni, N_{Avo} the Avogadro constant and $N_{atoms,surf}$ the number of surface atoms. It's worth underlying that in the experimental procedure it is not possible to directly apply Eq.(85) since the number of surface atoms is not directly accessible. The density of surface atoms ρ_{surf} is commonly estimated as the average value of the atomic density on the (111), (100) and (110) surface the nanoparticle is approximated to a sphere to calculate its surface:

$$\mathrm{TOF}'\left[\frac{1}{\mathrm{s}\,N_{atoms,surf}}\right] = \frac{\vec{R}}{4\pi\left(\frac{d}{2}\right)^2 \rho_{surf}} \tag{86}$$

However, as shown in Figure S14, this model underestimates the number of surface sites and the absolute value of the TOF will be affected by this error. The reason for the discrepancy lies in the fact that ρ_{surf} is computed as an arithmetic average of the surface densities of the three surfaces, while, as shown in Figure S24, the surface (111) covers about 90% of the nanoparticle.

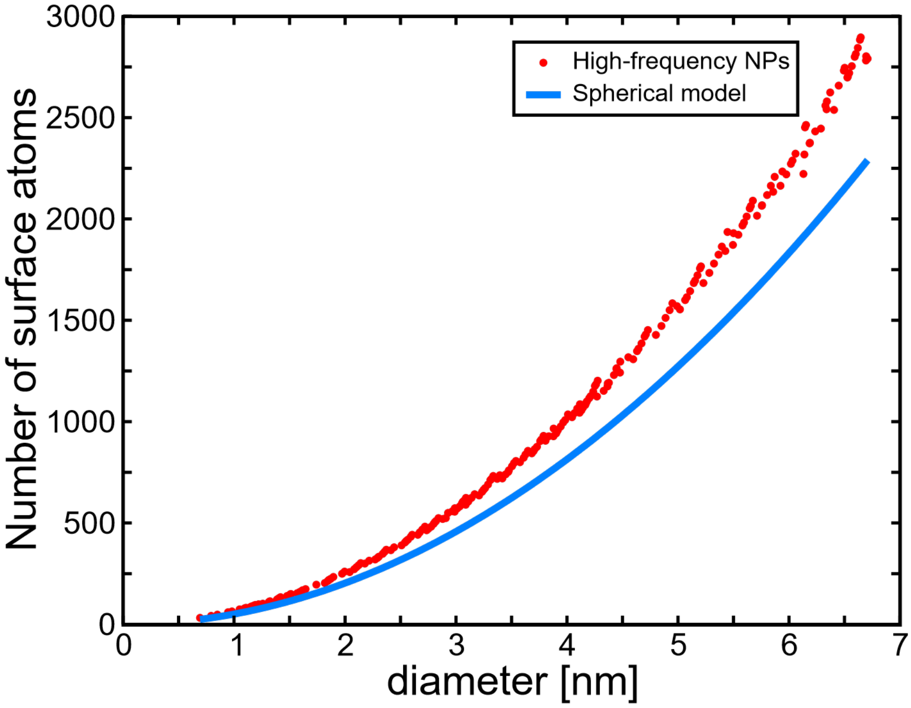

Figure S14: Number of surface atoms of the high-probability nanoparticles compared to the number predicted by a spherical model of the nanoparticles with uniform atomic density.

3.1 Active site contributions to the total reaction rate

The contribution of each site to the total reaction rateis computed as:

$$C_i = \frac{N_i \vec{r}_i}{\sum_k N_k \vec{r}_k} \quad (87)$$

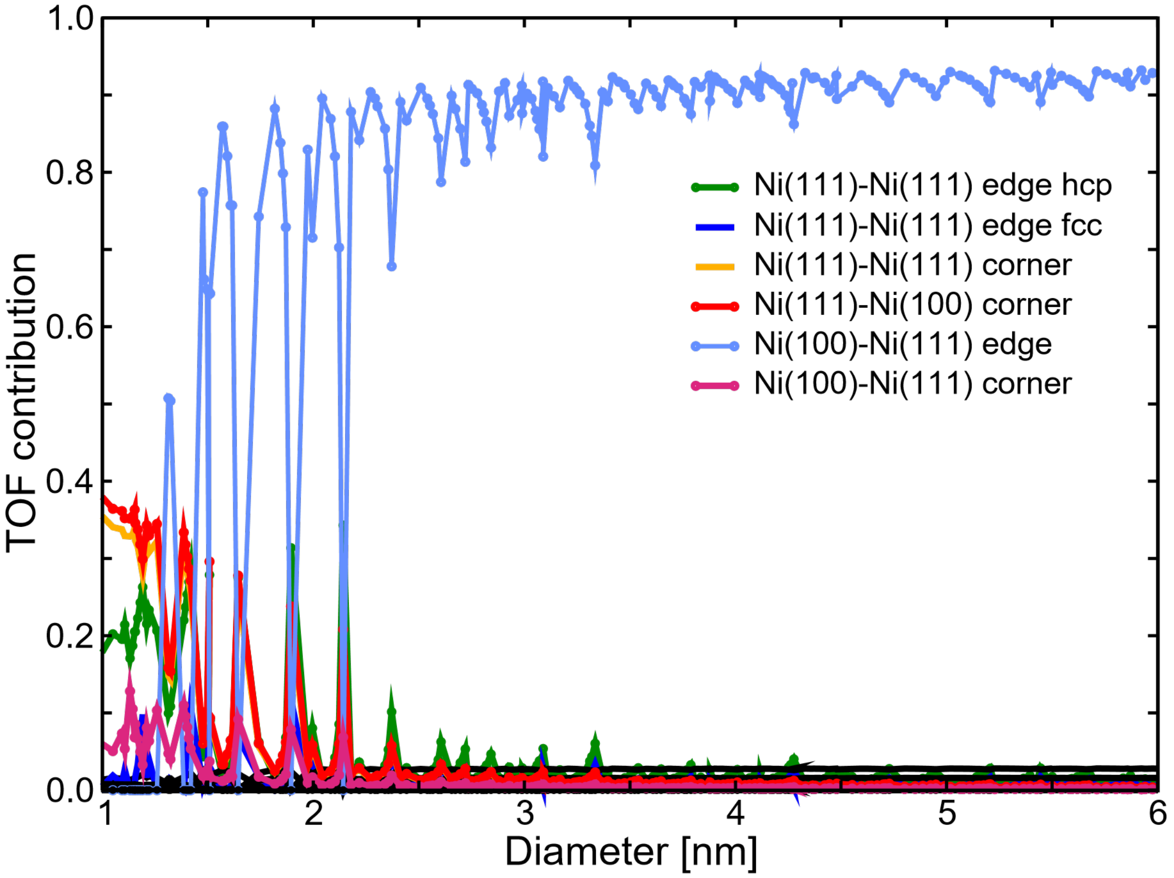

Figure S15: Contribution of each site to the total reaction rate.

3.2 Absolute value of turnover frequency

3.2.1 TOF at different conversion values

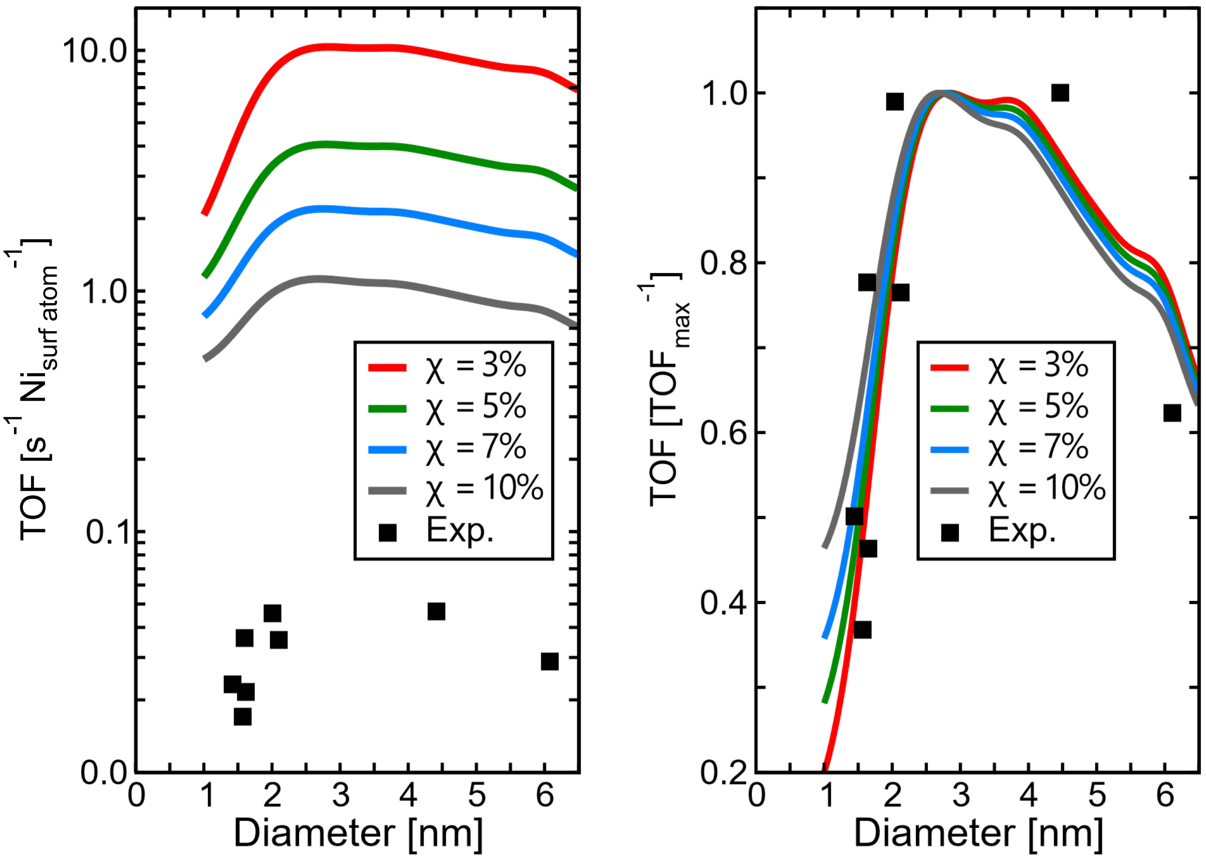

Figure S16: Left panel: absolute value of the TOF of the CO methanation reaction over the high-probability Ni metal nanoparticles as a function of the diameter, for different values of the conversion χ. Right panel: TOF reported in terms of the maximum value of each curve. The lines represent the gaussian smoothing of the data. While the absolute value of the TOF depends on the conversion, the relative trend does not change.

3.2.2 Energetic penalty to H^* due to lateral interactions

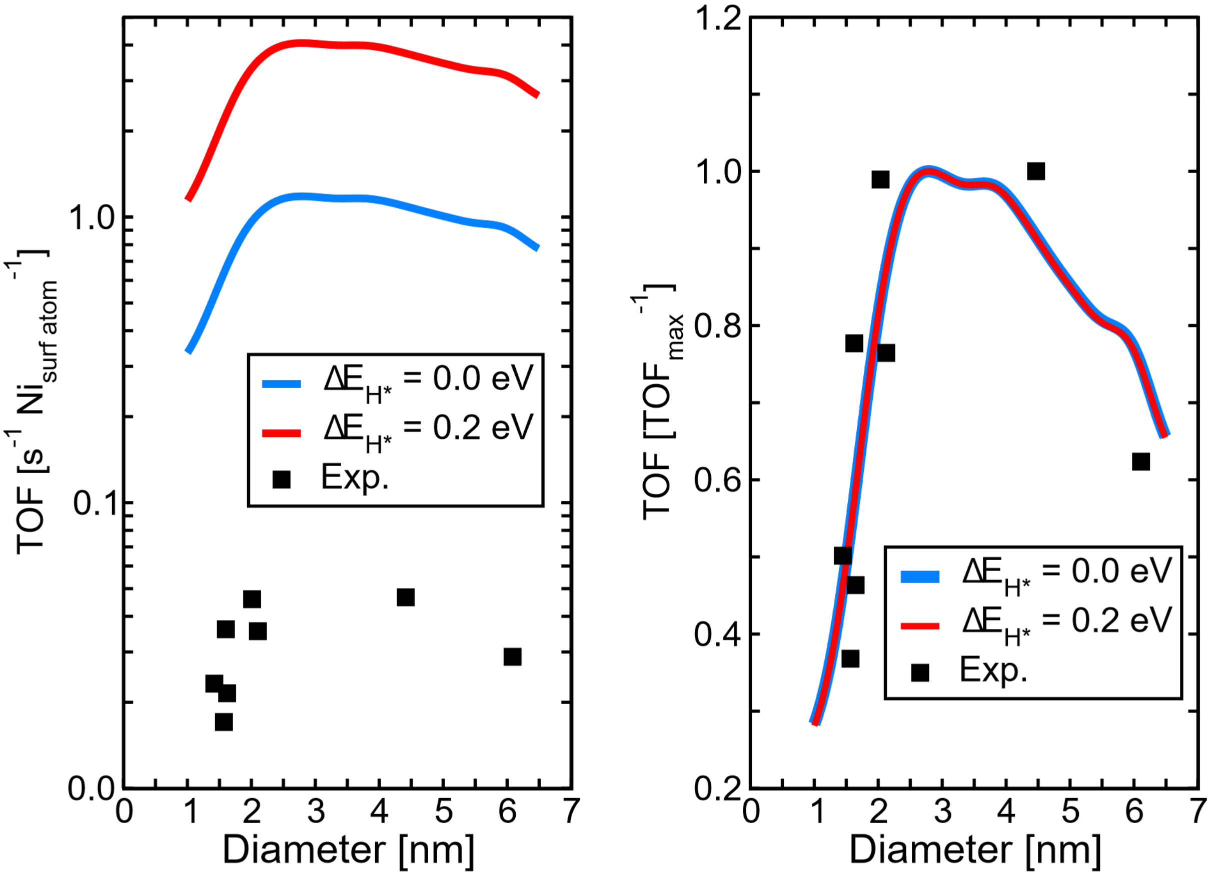

Figure S17: Left panel: absolute value of the TOF of the CO methanation reaction over the high-probability Ni metal nanoparticles as a function of the diameter, for two different values of the energetic penalty assignet to H*. Right panel: TOF reported in terms of the maximum value of each curve. The lines represent the gaussian smoothing of the data. While the absolute value of the TOF depends on the penalty, the relative trend does not change.

3.2.3 TOF at different CH_3*-C* lateral interactions

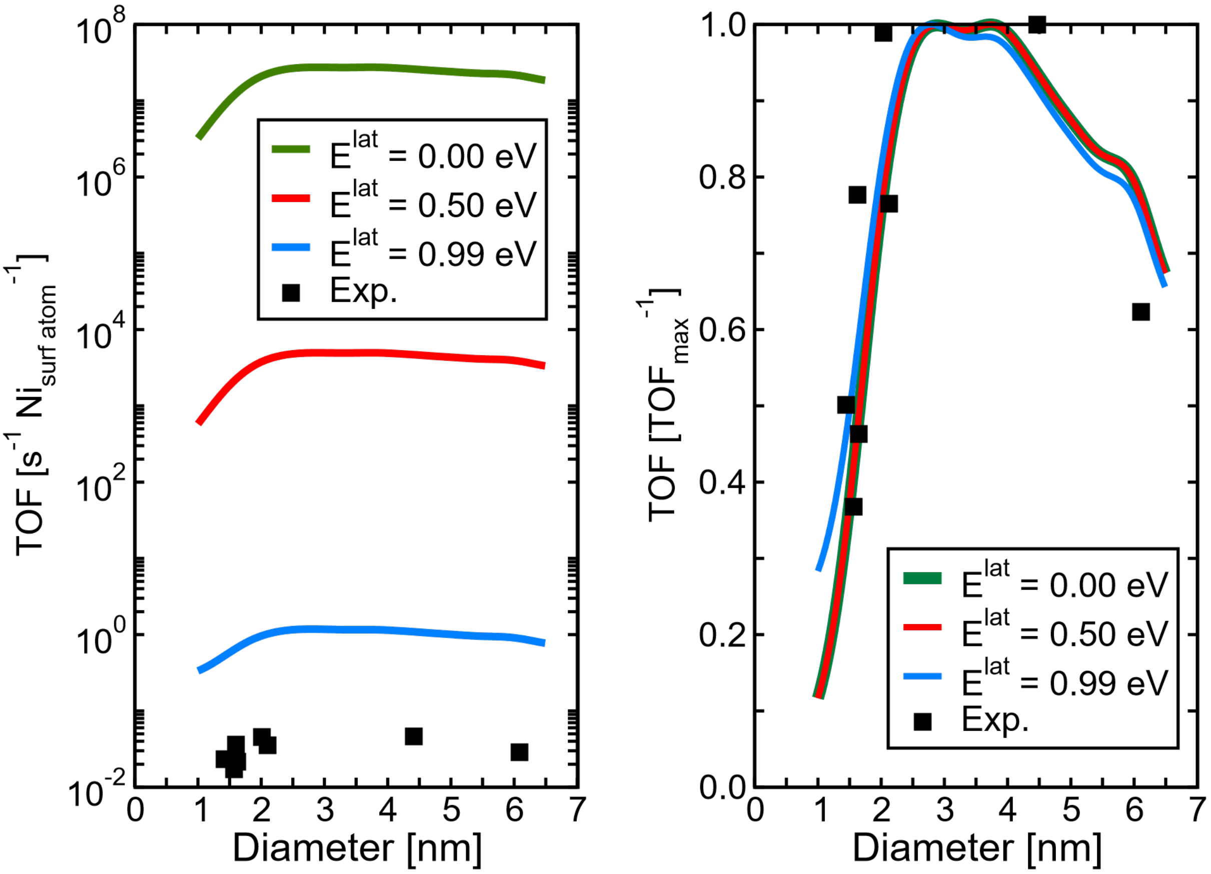

Figure S18: Left panel: absolute value of the TOF of the CO_2 methanation reaction over the high-probability Ni metal nanoparticles as a function of the diameter, for three different values of the CH*-C* lateral interaction. Right panel: TOF reported in terms of the maximum value of each curve. The lines represent the gaussian smoothing of the data. While the absolute value of the TOF strongly depends on the penalty, the relative trend does not change.

4 Simplified kinetic scheme of the CO_2 methanation reaction

We adopted a simplified kinetic scheme, in which the mechanism A presented above is taken as representative of the methanation reaction over the whole metal nanoparticle. In this sense, the HCO* dissociation reaction represents the RDS over all the sites, and CO* is the only relevant reaction intermediate. The value of k_{app} and K_{CO} are reported in Table S8 and Table S9 , respectively.

Table S8: Gibbs free energy of the transition state of the HCO* dissociation reaction and corresponding apparent rate constants k_{app}, evaluated on the different active sites.

**Facet**	**Site**	**Reaction**	$\widetilde{\mathbf{G}^{0}_{TS,5}}$ [eV]	$\mathbf{k_{app}}$ [1/s]
(111)	Terrace	Intra-face	2.22	$3.52 \cdot 10^{-4}$
(111)	Edge fcc	Intra-face	2.15	$1.04 \cdot 10^{-3}$
(111)	Edge fcc	Interface (111)-(111)	1.83	$2.77 \cdot 10^{-1}$
(111)	Edge fcc	Interface (100)-(111)	1.74	$1.53 \cdot 10^{0}$
(111)	Edge hcp	Intra-face	1.93	$4.91 \cdot 10^{-2}$
(111)	Edge hcp	Interface (111)-(111)	1.81	$3.72 \cdot 10^{-1}$
(111)	Edge hcp	Interface (100)-(111)	1.77	$7.17 \cdot 10^{-1}$
(111)	Corner	Intra-face	2.00	$1.58 \cdot 10^{-2}$
(111)	Corner	Interface (111)-(111)	1.83	$2.57 \cdot 10^{-1}$
(111)	Corner	Interface (100)-(111)	1.82	$3.05 \cdot 10^{-1}$
(100)	Terrace	Intra-face	1.77	$8.23 \cdot 10^{-1}$
(100)	Edge	Intra-face	1.83	$2.67 \cdot 10^{-1}$
(100)	Edge	Interface (100)-(111)	1.55	$3.20 \cdot 10^{1}$
(100)	Corner	Intra-face	1.95	$3.80 \cdot 10^{-2}$
(100)	Corner	Interface (100)-(111)	1.69	$3.19 \cdot 10^{0}$

The activity and the TOF computed within this scheme are reported in Figure S19. The trend is reproduced even in this simplified scheme, further highlighting the importance of the edge sites at the Ni(100)-Ni(111) interface, which modulate the trend.

Table S9: Gibbs free energy and corresponding equilibrium constant of CO adsorption evaluated on the different active sites.

Facet	Site	$\widetilde{G^0_{CO*}}$[eV]	$k_{CO*}[1/s]$
(111)	Terrace	0.12	0.1165
(111)	Edge fcc	0.12	0.1165
(111)	Edge hcp	0.15	0.0691
(111)	Corner	0.14	0.0964
(100)	Terrace	0.18	0.0426
(100)	Edge	0.17	0.0544
(100)	Corner	0.18	0.0459

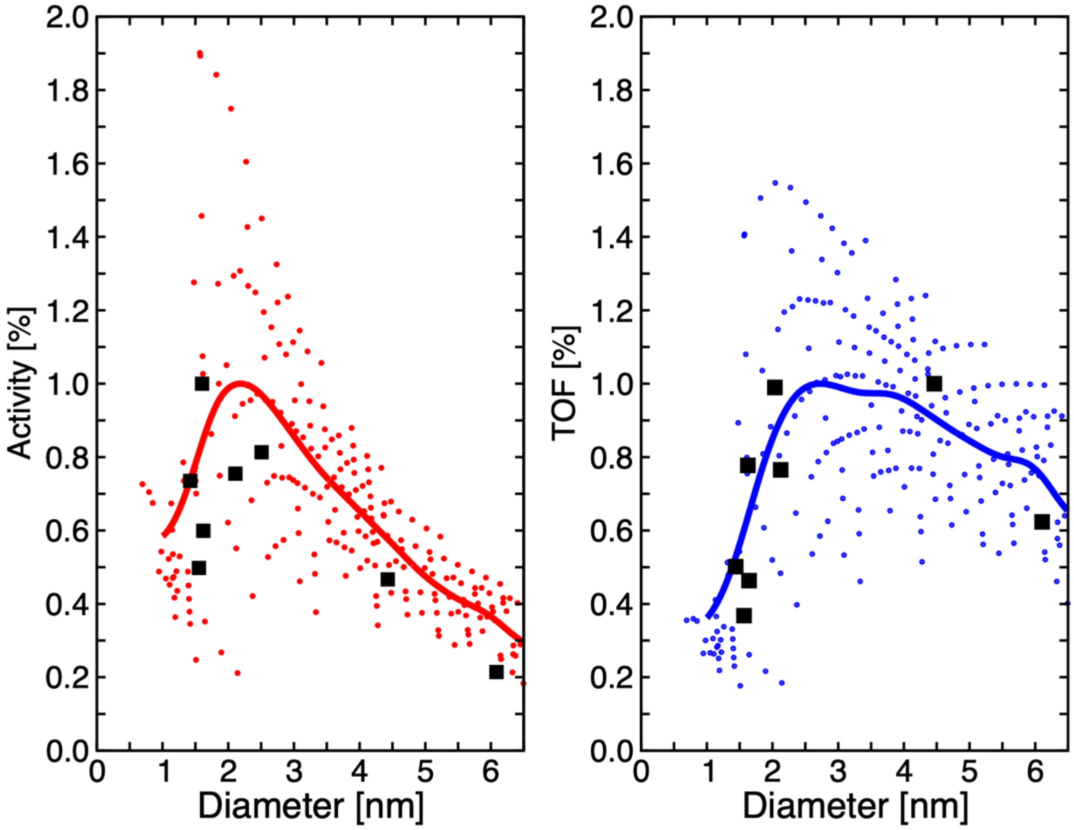

Figure S19: Activity (left) and TOF (right) computed with the simplified kinetic scheme and filtered with a gaussian smearing and normalized with respect to the maximum value. The experimental data of Vogt et al.[S7] are reported as black dots.

5 Identification of nanoparticles with the highest probability of occurrence

5.1 Ensemble of Ni nanoparticles

We generated an ensemble of Ni nanoparticles, representative of the plethora of sizes and shapes that they can take under reaction conditions. The procedure for creating the nanoparticles is the same as the one employed by Cheula et al.,[S8] namely cleaving a huge bulk of Ni with combinations of lattice planes at different distances from a central point. The distance from the centre of the construction is not proportional to the surface free energy, as in the Wulff construction, but is set to integer multiples of the inter-planar distance of the corresponding facet. Iterating these numbers to cover all the possible combinations below a certain threshold it is possible to generate metal nanoparticles exposing all the combinations of the surfaces under consideration. We took into account only the fcc bulk phase of Ni, thus avoiding the creation of multiply-twinned nanoparticles having icosahedral or decahedral bulk phase. The lattice planes considered for creating the nanoparticles in the ensemble are both low and high-Miller indices planes: (100), (110), (111), (210), (211), (311). Additionally, to take into account possible kinks and defects occurring on small nanoparticles, we have build a denser set of nanoparticles with a number of atoms lower than 300, corresponding to a particle size smaller than 1.6 nm. This denser set was constructed both by changing the position of the central point relative to which the lattice planes are placed and removing selectively atoms from the existing nanoparticles, in order to have continuity in the number of atoms. The resulting ensemble consists of around 60000 nanoparticles containing from 30 to 16000 Ni atoms and spanning a range of sizes between 0.8 nm to 6.5 nm.

5.1.1 Nanoparticles diameter

The diameter of each nanoparticle is computed as:

$$d = \sqrt[3]{\frac{6V}{\pi}} \quad , \tag{88}$$

where V is the volume of the convex hull connecting all the surface atoms, as described by Cheula et al..[S8] The diameter of the nanoparticles of the ensemble is reported in Figure S20 as a function of the number of atoms of each nanoparticle.

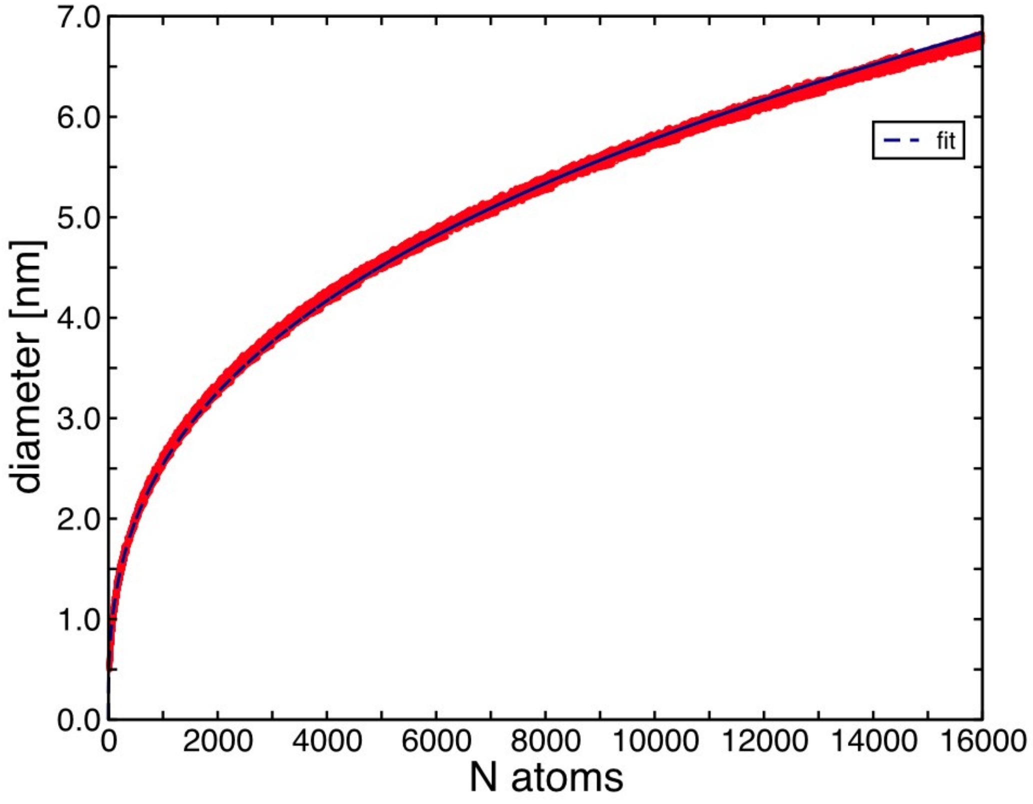

Figure S20: Diameter of the nanoparticles in the ensemble as a function of the number of atoms and fit function.

We fit the data using a power-law formula, in order to find a dependence of the diameter on the number of atoms in the nanoparticle:

$$d = A(N_a)^B \quad . \tag{89}$$

The values that best fit our data are A = 0.2163 nm and B = 0.3567, which is close to value of 1/3, which could be obtained by assuming a spherical shape for the nanoparticles.

5.2 Formation energy model

The relative stability of each nanoparticle within the ensemble is determined uniquely by its formation energy. However, the direct calculation of the DFT energy of the nanoparticles can be extremely computationally demanding both for the huge number of nanoparticles in the ensemble and for the number of Ni atoms inside each nanoparticle. Therefore, we adopted the surrogate model of Cheula et al.[S8] to compute the formation energy of a nanoparticles with N_{atoms} atoms depending only on its geometric configuration:

$$E_f^{NP}(N_{atoms}) = E_{coh}^{bulk}\left[\sum_i^{N_{atoms}}\left(\frac{\sqrt{CN_i}}{\sqrt{12}}\right) - N_{atoms}\right] + \sum_i^{N_{atoms}} E_i^{relax}(CN_i) \quad . \tag{90}$$

The first term on the right-hand side of Eq.(90) represents the formation energy computed using the square-root bond cutting model,[S9,S10] which depends only on the square root of the coordination number (CN) of each atom inside the nanoparticle. E_{coh}^{bulk} is the cohesive coh energy of fcc bulk Ni, defined as the energy difference between one Ni atom in the bulk and an isolated Ni atom in gas phase and at the DFT level it is equal to −4.82 eV. The relaxation term E_i^{relax} in Eq.(90) takes into account the fact the under-coordinated atoms of the nanoparticles will relax their position gaining energy.

The relaxation energy (E_i^{relax}) as a function of the coordination number is calculated by fitting DFT relaxation energies using a power law formula:

$$E_i^{relax} = A(12 - CN_i)^B \quad . \tag{91}$$

The DFT relaxation energies are obtained by periodic slab calculations, computing the energy difference per relaxed atom between the structure in which all the atoms are kept fixed at the distances of the bulk and the structure in which all the atoms with a specific coordination number are allowed to relax. We obtain $A = -1.1 \cdot 10^{-4}$ eV and $B = 3.5$ as the parameters that best fit our data, as reported in Figure S21.

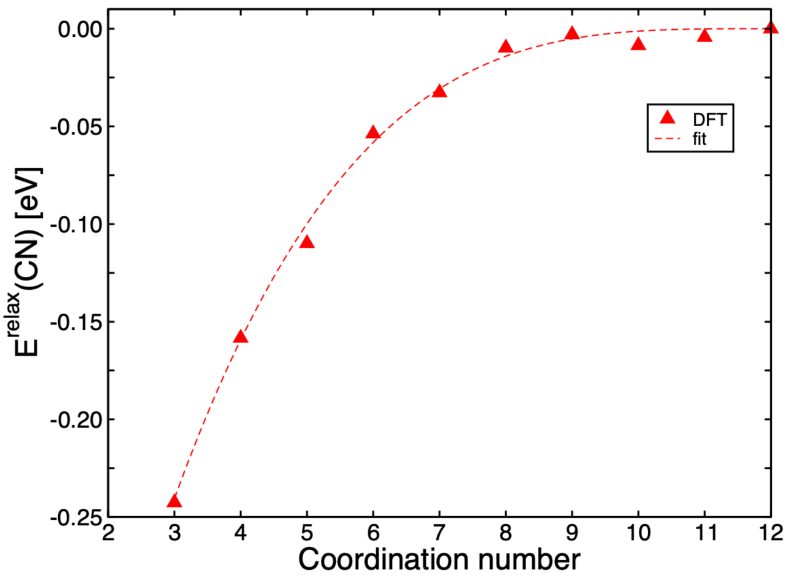

Figure S21: DFT relaxation energy and fit function.

In constructing the Boltzmann distribution we approximated the formation free energies with the formation energies, $G^{NP}(N_{atoms}) \simeq E^{NP}(N_{atoms})$, since the entropic and vibrational contribution at the temperatures of interest is almost independent from the coordination number of the atoms, thus resulting in a rigid shift.

5.2.1 Benchmark of the energy model

The formation energies computed using Eq.(90) were benchmarked against the formation energies computed at the DFT level in absence and presence of CO for a set of periodic slabs, representing some low Miller indices surfaces, and some small nanoparticles, from 38 to 147 atoms.

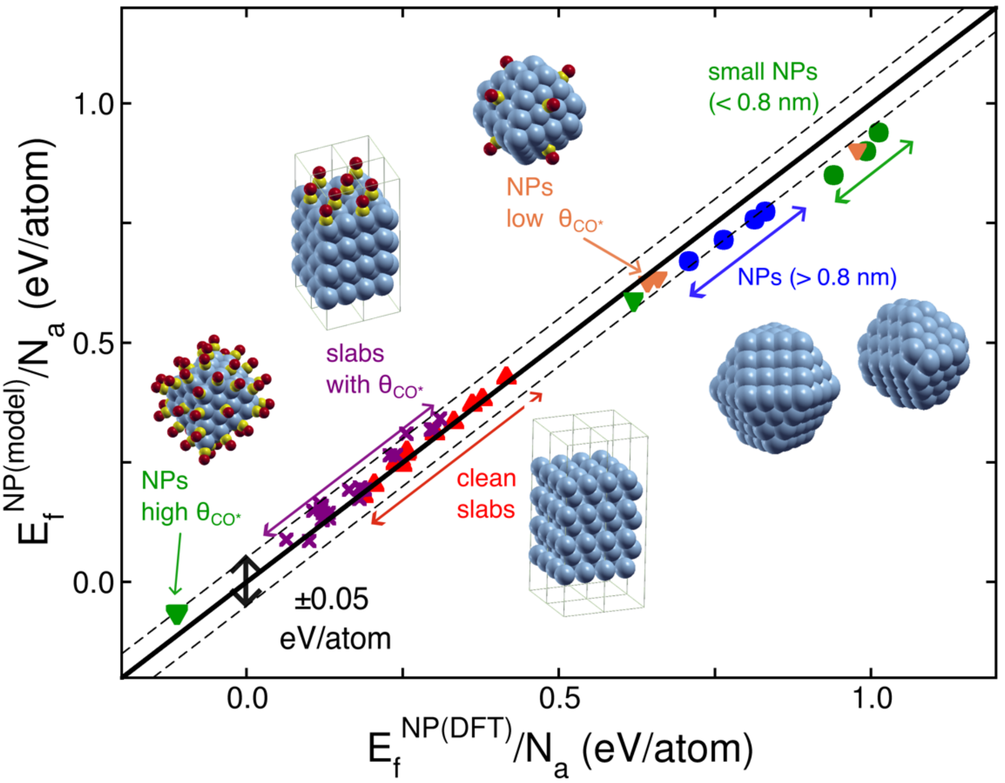

Figure S22: Parity plots of formation energies (per Ni atom) of selected structures calculated with the model described in this work and with DFT relaxation calculations.

A comparison between the formation energies normalized by the number of atoms, is reported in the parity plot in Figure S22. A part from nanoparticles smaller than 0.8 nm, in which confinement effects are enhanced, the model correctly predicts the formation energies of the considered structures both in presence and absence of CO, with a tolerance of 0.05 eV/atom.

5.3 Boltzmann statistics: an example of application

The application of the Boltzmann statistics to the ensemble as a whole would provide information on the most probable metal nanoparticles regardless their size. However, our goal is to determine how the shape of the metal nanoparticle evolves with the particle size to in- terpret the experimental data in which there are different metal nanoparticles distributions, each centered on a specific average size. Therefore, we computed the probability of occur- rence of each metal nanoparticle in the ensemble using a weighted Boltzmann distribution, described in the main text.

We report here a practical example on how the method works, focusing on the range of N_{atoms} between 1650 and 1850.

The formation energy of the nanoparticles in this range are reported in Figure S23(a), where we highlighted with blue squares the nanoparticles with the highest frequency at the end of the procedure, whose crystal structure is reported in the upper part of the panel. In Figure S23(b) we report a histogram showing the probability of occurrence of the nanoparticle for different values of N_{target} ranging from 1680 to 1800. The value of σ in the weight function $\omega(N_i)$ has been set to 5. Only 8 nanoparticles among the 713 contained in this range are relevant, showing a high probability of occurrence. In particular, as shown in Figure S23(b), a specific nanoparticle can have a high probability of occurrence for different values of N_{target}, with a maximum when the number of atoms is close to N_{target}. The size of the 8 relevant nanoparticles lie between 3.05 nm and 3.2 nm. Repeating the procedure for different values of N_{target}, it is possible to find the high-probability nanoparticles at any particle size.

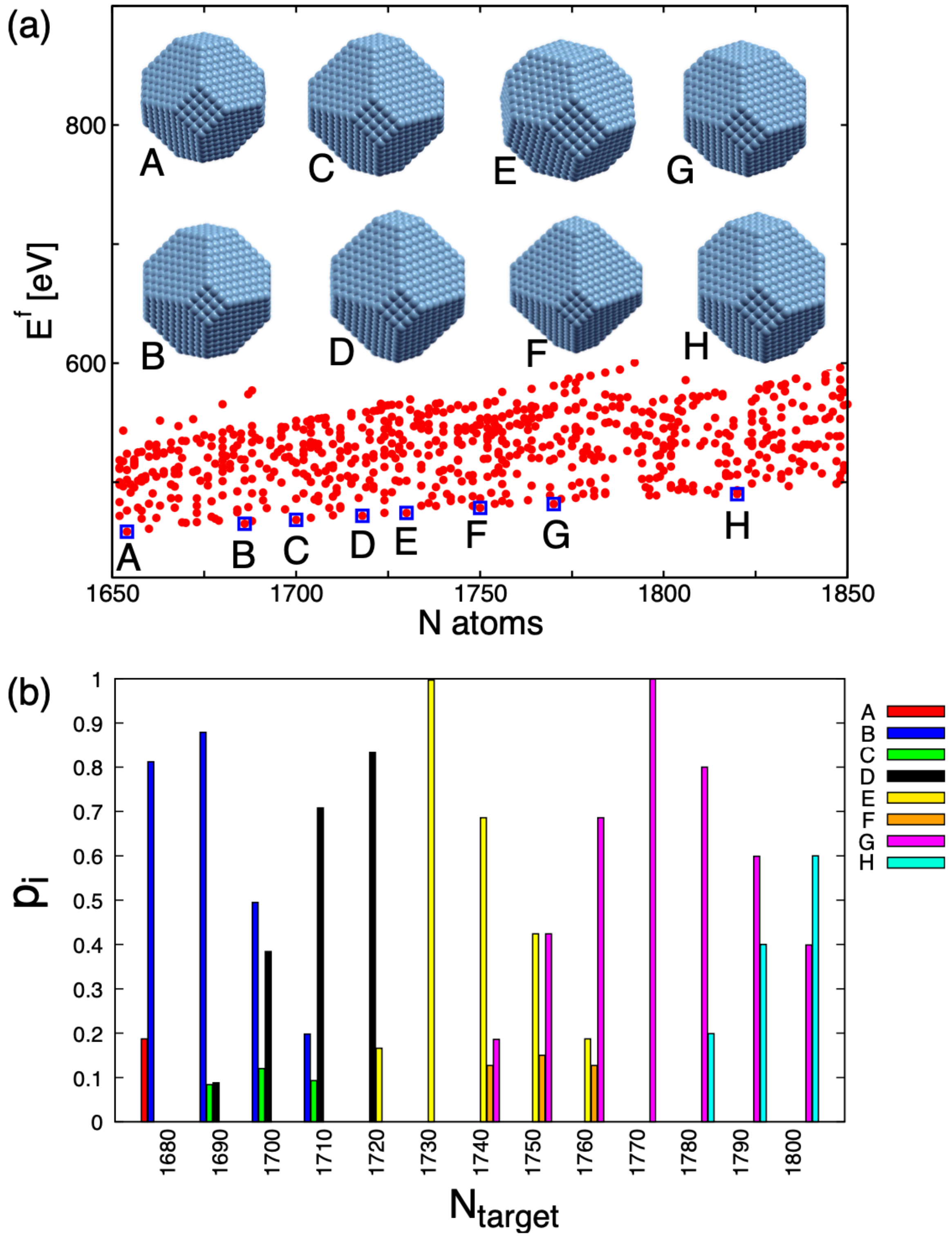

Figure S23: (a) Formation energies of the nanoparticles in the ensemble in the range of N_{atoms} between 1650 and 1850 (red points). Blue squares highlight the nanoparticles with high probabily of occurrence and the corresponding crystal structure is reported in the top panel. (b) Probability of finding the eight relevant nanoparticles for different values of N_{target}.

5.4 Composition of the high-probability nanoparticles

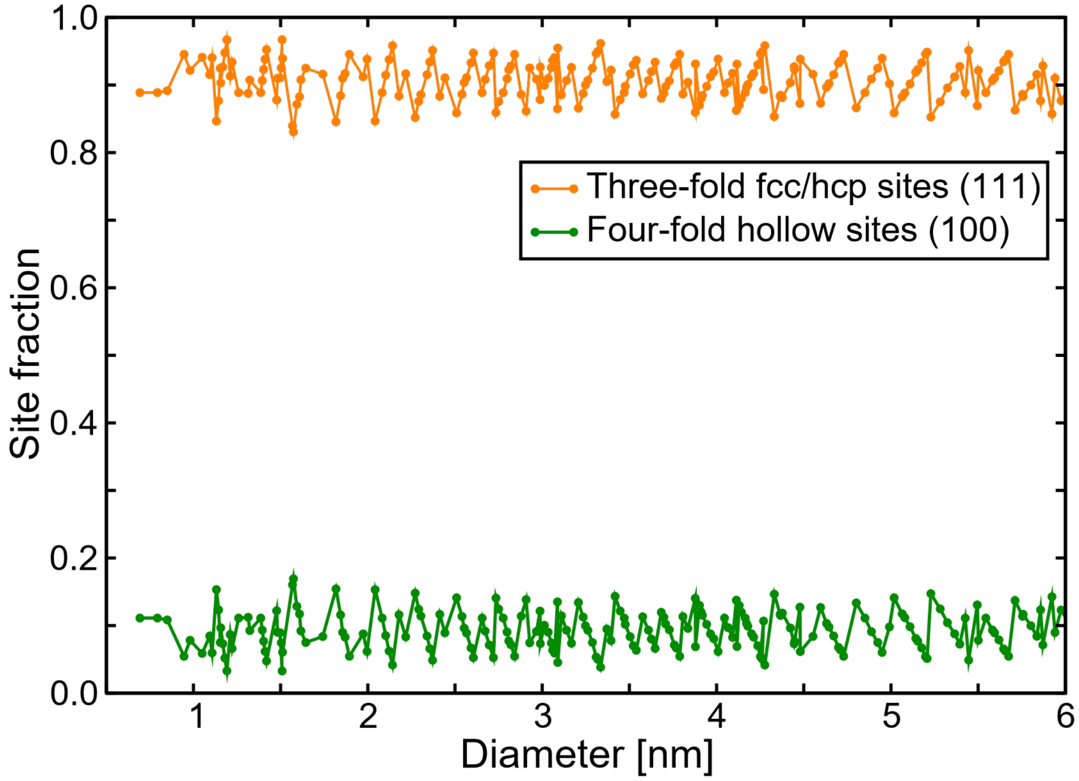

Figure S24: Comparison of the fraction of threefold fcc/hcp sites and fourfold hollow sites in the high-probability nanoparticles as a function of size.

6 Active sites distribution in presence of CO*

The energy model of Cheula et al.[S8] can be extended to account for the presence of adsorbates. CO* has been reported to be the most abundant reaction intermediate (MARI) of the CO methanation reaction[S2,S7] and it is known to be the main responsible for the modification of the catalyst morphology under reaction conditions.[S11] The formation energy of a nanoparticle with N_{atoms} Ni atoms in the presence of N_{CO*} adsorbed CO molecules is computed as:

$$E_f^{NP}(N_{atoms}, N_{CO*}) = E_f^{NP}(N_{atoms}) + \sum_{i}^{N_{CO*}} E_{bind}^{CO*\,(i)} \quad , \tag{92}$$

where $E_f^{NP}(N_{atoms})$ is the formation energy of the clean nanoparticle and $E_{bind}^{CO*\,(i)}$ takes into account the effect of the metal-adsorbate interaction $E_{bind,(0)}^{CO*(i)}$ and adsorbate-adsorbate interaction $\Delta E_{bind}^{CO*(i)}$:

$$E_{bind}^{CO*(i)} = E_{bind,(0)}^{CO*(i)}(CN_i) + \Delta E_{bind}^{CO*(i)}(\theta_{CO*}) \quad . \tag{93}$$

The former term in the right-hand side of the above equation represents the binding energy at zero coverage of CO* over the i-th active sites, formed by Ni atoms having an average coordination number CN_i. Unlike rhodium, where CO adsorbs mainly on top of Rh atoms, there are several different adsorption sites over Ni surfaces, such as bridge, hollow, fcc/hcp, long bridge or step-edge. To provide a model for $E_{bind,(0)}^{CO*(i)}(CN_i)$, we analyze the adsorption of CO over the various active sites with different coordination numbers. The binding energy is computed taking as a reference the clean slab adopted to model the surface and the isolated CO molecule, and we added *a-posteriori* the correction of Mason et al., cfr Eq.(51). We evaluated the binding energy of CO over different Miller index surfaces such as (100), (110), (111), (210), (211), (311), (321), (331) and defective (100) and (111) to account for different coordination number of each active site. We fit the binding energy of CO* over each site with a straight line to catch a general trend as a function of the coordination number, as shown in Figure S25.

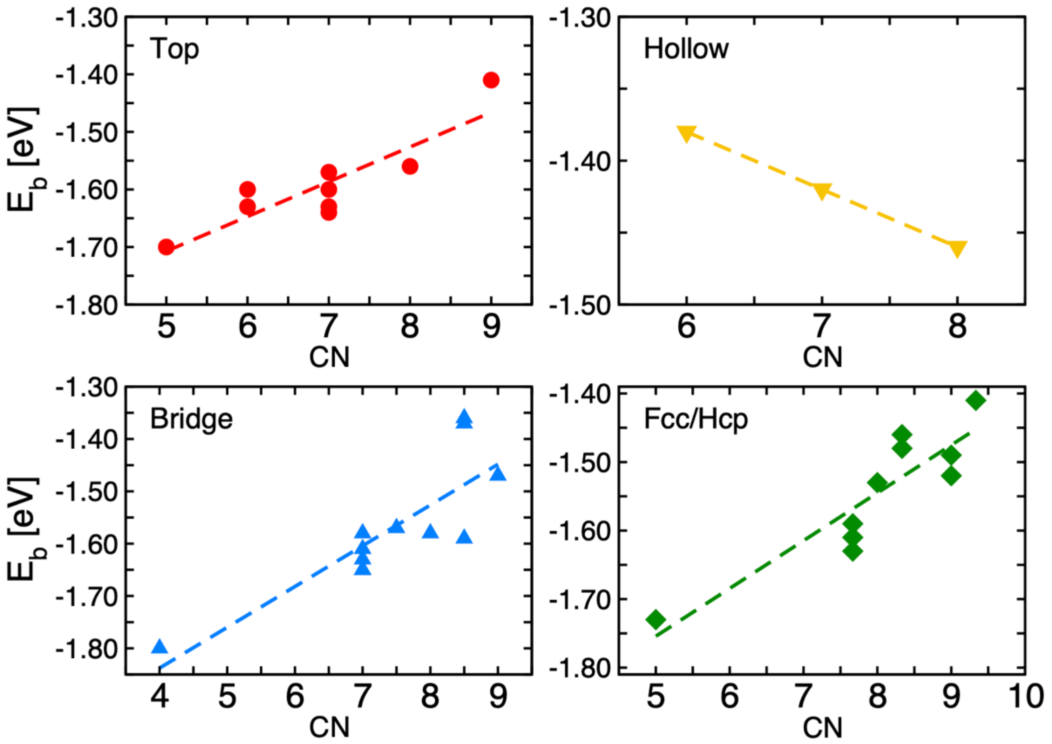

Figure S25: Binding energy of CO* as a function of the average coordination number of the site for different active sites.

The lateral interaction among the adsorbates $\Delta E_{bind}^{CO*(i)}(\theta_{CO*})$ was computed evaluating the change of the binding energy of CO* as a function of the ratio between the number of adsorbed CO* molecules (N_{CO*}) and the surface area of the unit cell available for the adsorption. We employed the low-Miller index surfaces (100), (110) and (111) and we evaluate the binding energy changing the number of adsorbed CO* molecules over different active sites, accounting also for ordered adsorption patterns reported in the literature.[S12, S13] For each value, $\Delta E_{bind}^{CO*(i)}(\theta_{CO*})$ is computed as the deviation with respect to the binding energy at zero coverage. The results reported in Figure S26 are fitted with a power law, whose optimal parameters were found to be $\alpha = 232.469$ and $\beta = 3.0644$.

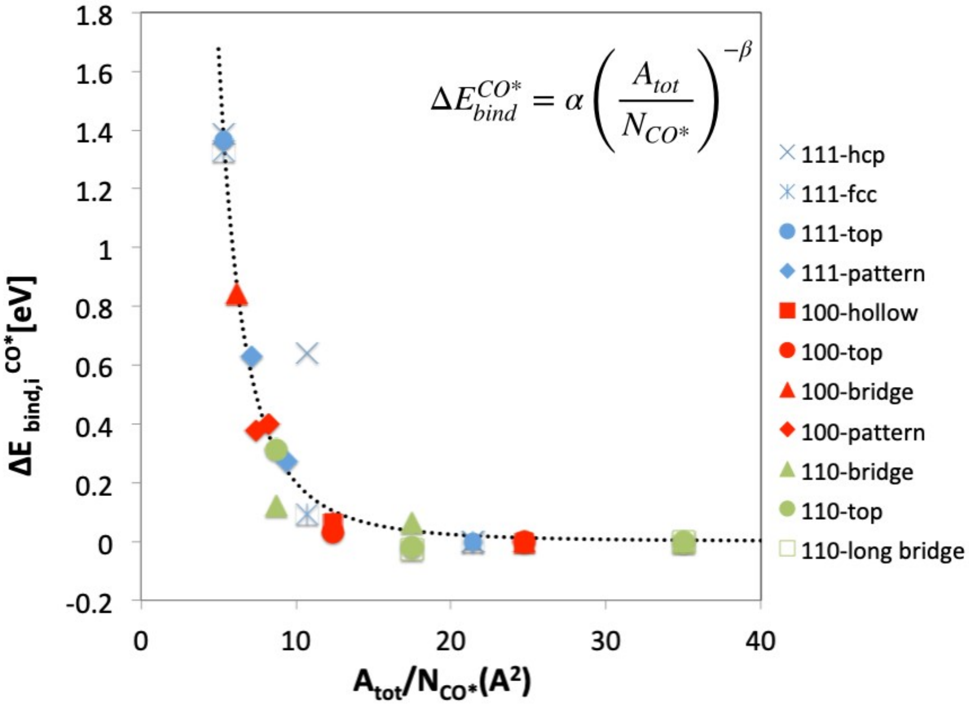

Figure S26: Binding energy of CO* as a function of the average coordination number of the site for different active sites.

The Gibbs free energy of formation of a nanoparticle at the temperature and pressure adopted in the experiments is computed as:

$$G_f^{NP}(N_{atoms}, N_{CO*}) = E_f^{NP}(N_{atoms}, N_{CO*}) + \sum_{i}^{N_{CO*}} \left(\Delta G_{CO*}^{(i)}(CN_i, T) - \Delta\mu_{CO}^{gas}(T, p)\right) \quad , \qquad (94)$$

where the term within the sum accounts for the difference in the Gibbs free energy between the adsorbed and the gas-phase CO molecule. We computed the enthalpic and entropic contributions of the CO* molecules evaluating the vibrational frequencies within the harmonic approximation. The ideal gas scheme has been adopted to model the gas-phase CO molecule:

$$\Delta\mu_{CO}^{gas}(T, p) = \Delta\mu_{0,CO}^{gas}(T, p) + k_B T \cdot \ln\left(\frac{P_{CO}}{P_0}\right) \quad , \qquad (95)$$

where P_0 is the reference pressure, equal to 1 atm. We set T = 673 K and P_{CO} equal to 0.0002 atm, consistent with a conversion of 7% according to Table S7.

At these thermodynamic conditions, the equilibrium coverage of CO* is determined minimizing the Gibbs free energy $G_f^{NP}(N_{atoms}, N_{CO*})$ with respect to the number of CO* adsorbates, in which the negative adsorption term competes with the positive lateral interaction. o account for the fact that CO can adsorb on different types of active sites over Ni, we compute the

equilibrium coverage distributing each adsorbate over all possible sites with probability dependent on the binding energy on the site itself:

$$p(i, CN_i) = \frac{\exp\left(G_{bind}^{CO*(i)}(CN_i)\right)}{\sum_k \exp\left(G_{bind}^{CO*(k)}(CN_k)\right)} \quad , \tag{96}$$

where $G_{bind}^{CO*(i)}(CN_i)$ is the binding free energy of CO* over the site i, formed by atoms with an average coordination number CN_i. This term is given by the sum of the electronic binding energy and the entropic and enthalpic contribution:

$$G_{bind}^{CO*(i)}(CN_i) = E_{bind}^{CO*(i)}(CN_i) + \Delta G_{CO*}^{(i)}(CN_i, T) - \Delta\mu_{CO}^{gas}(T, p) \quad . \tag{97}$$

The lateral interaction has been evaluated using the power law shown in Figure S26, in which the area per atom corresponds to the surface of the nanoparticle divided by the number of adsorbates. For each nanoparticle in the ensemble, we compute the equilibrium CO* coverage and the corresponding Gibbs free energy of formation, Eq.(94), is used to construct the Boltzmann distribution and determine the nanoparticles with the highest frequency of occurrence. In order to make a direct comparison of the nanoparticles with high frequency of occurrence in the presence and in the absence of CO* we report in Figure S27-S32 the formation energy of the clean nanoparticles in different ranges of N_{atoms}, highlighting with different colors the high-probability nanoparticles in the two cases.

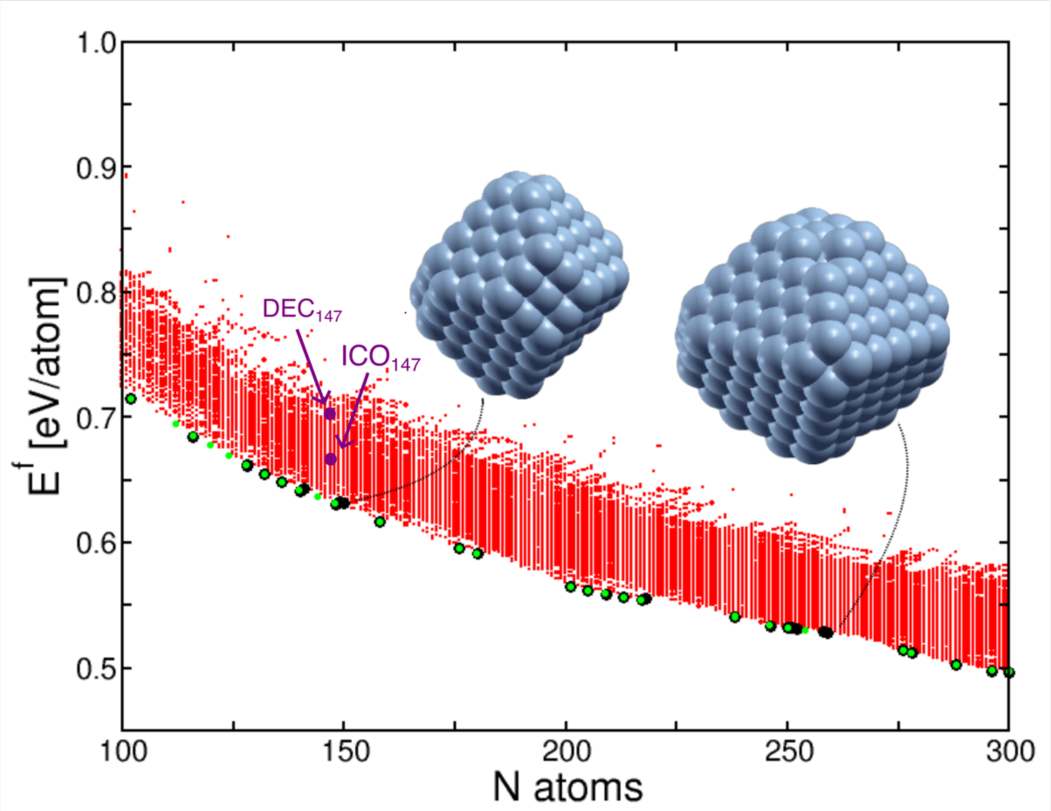

Figure S27: Formation energy of the clean nanoparticles in the range of N_{atoms} between 100 and 300 (red dots) evaluated with the surrogate model adopted in this work. High-probability nanoparticles in the absence and in the presence of CO* are highlighted with green and black circles, respectively. The crystal structure of selected nanoparticles is reported. DFT-relaxation formation energies of decosahedral and icosahedral NPs with 147 atoms are reported with purple dots, highligthing their higher energy with respect to fcc NPs of the same size.

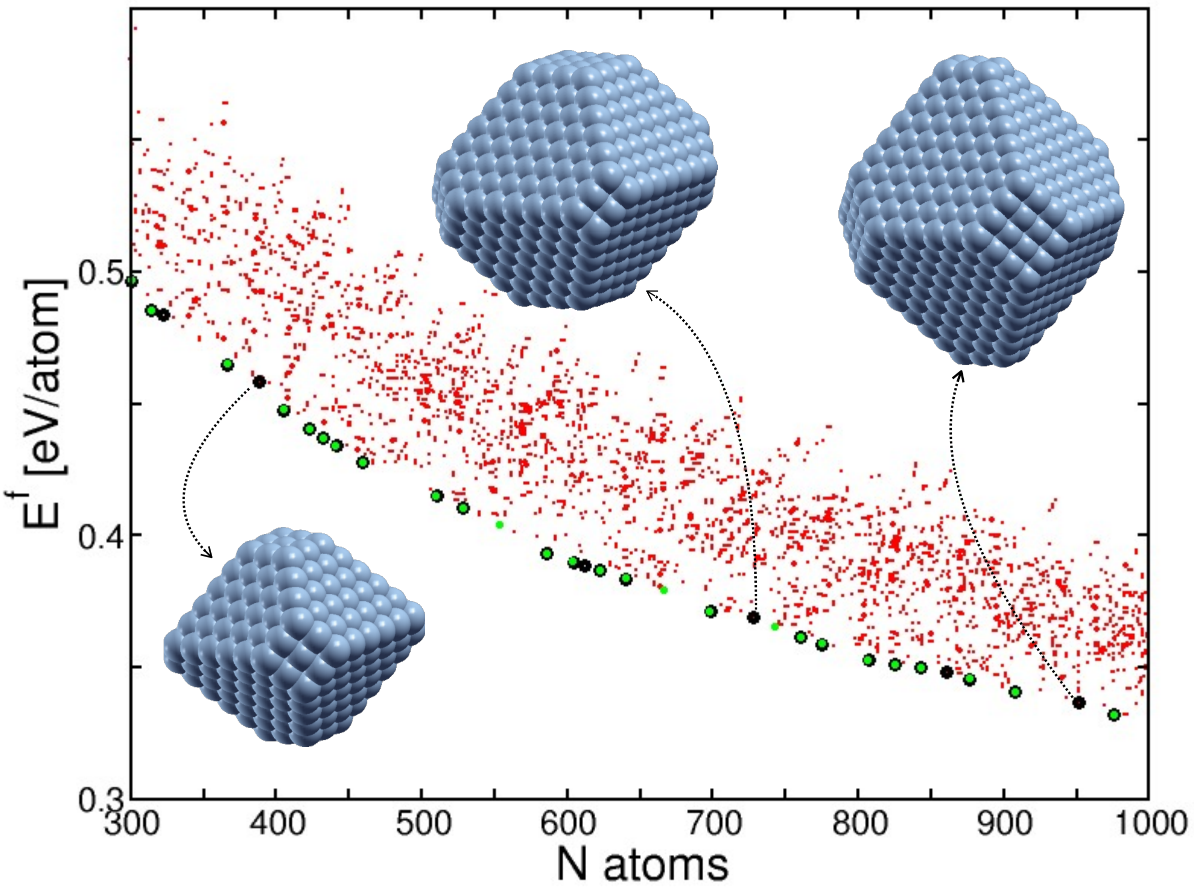

Figure S28: Formation energy of the clean nanoparticles in the range of N_{atoms} between 300 and 1000 (red dots). High-probability nanoparticles in the absence and in the presence of CO* are highlighted with green and black circles, respectively. The crystal structure of selected nanoparticles is reported.

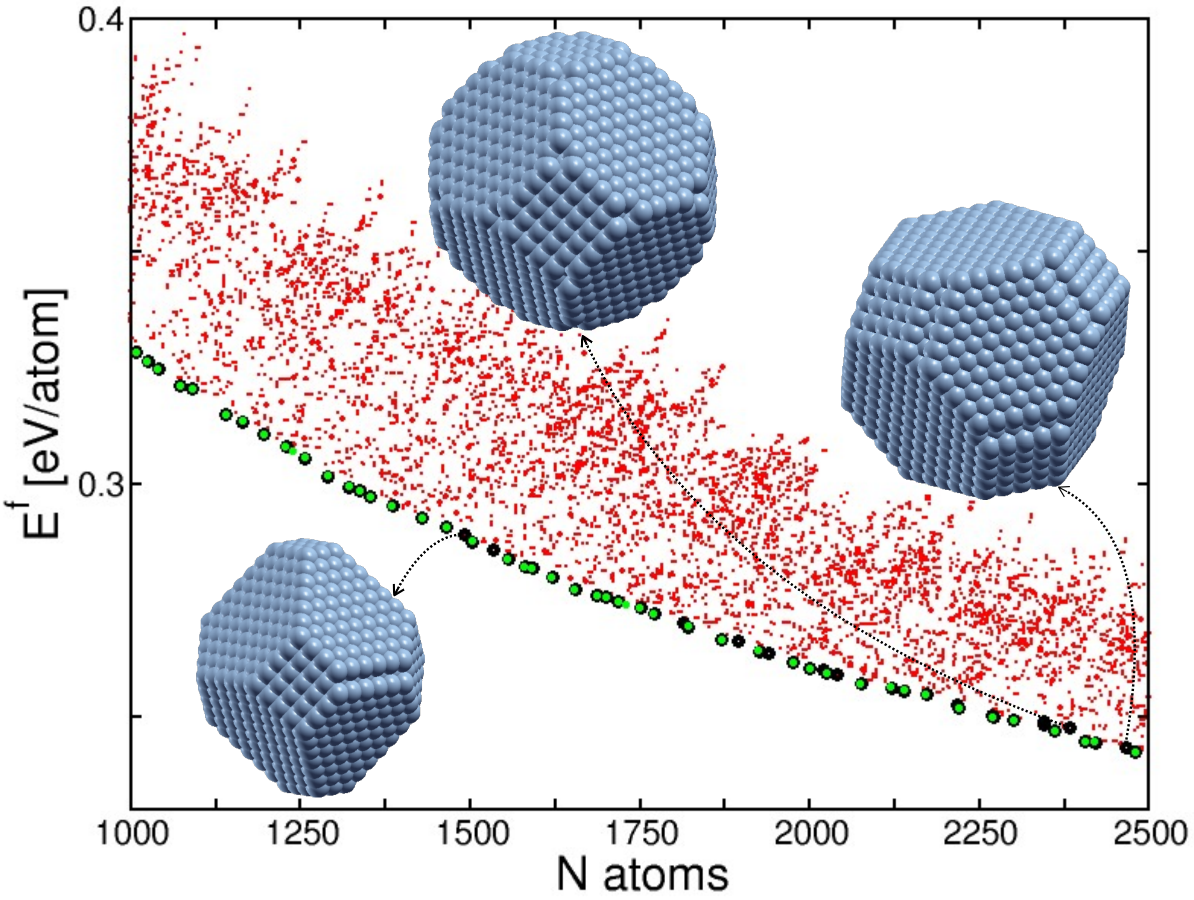

Figure S29: Formation energy of the clean nanoparticles in the range of N_{atoms} between 1000 and 2500 (red dots). High-probability nanoparticles in the absence and in the presence of CO* are highlighted with green and black circles, respectively. The crystal structure of selected nanoparticles is reported.

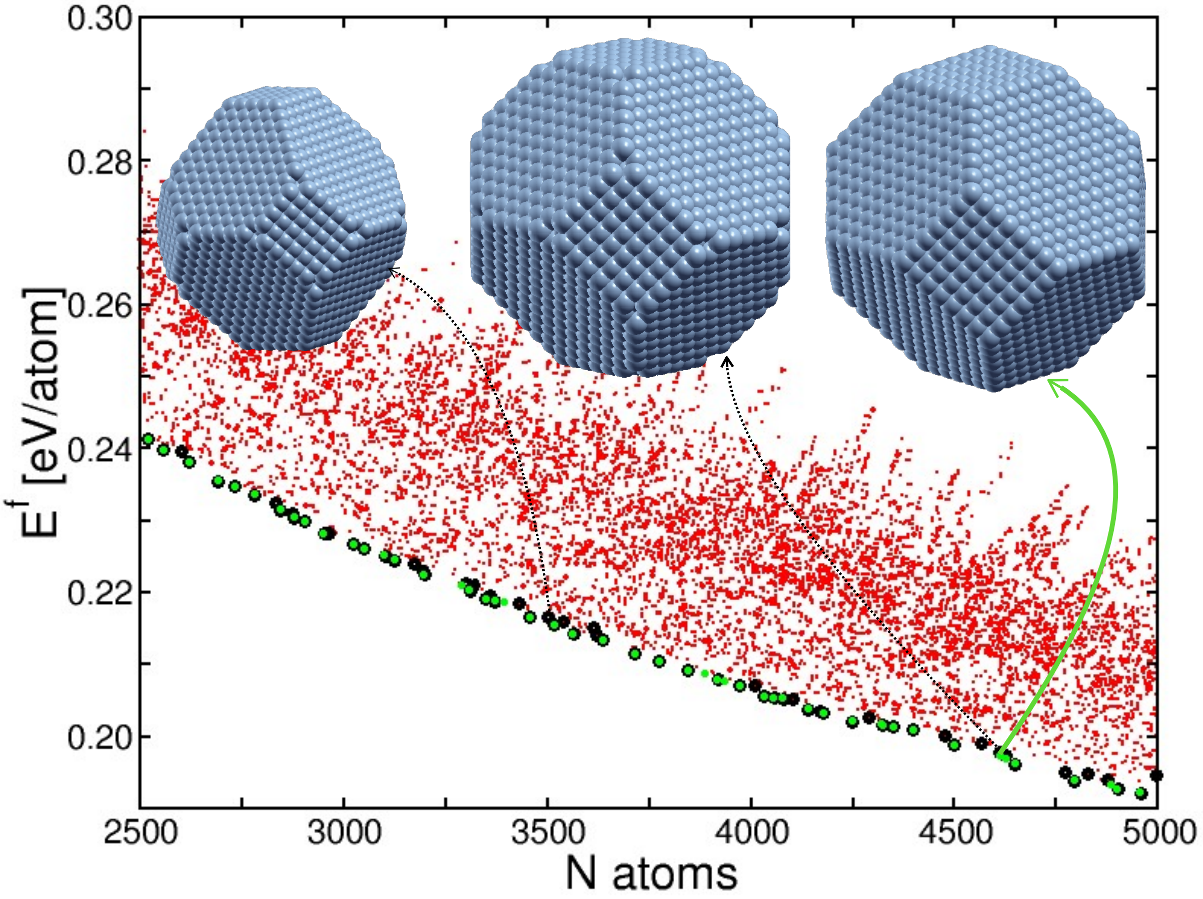

Figure S30: Formation energy of the clean nanoparticles in the range of N_{atoms} between 2500 and 5000 (red dots). High-probability nanoparticles in the absence and in the presence of CO* are highlighted with green and black circles, respectively. The crystal structure of selected nanoparticles is reported.

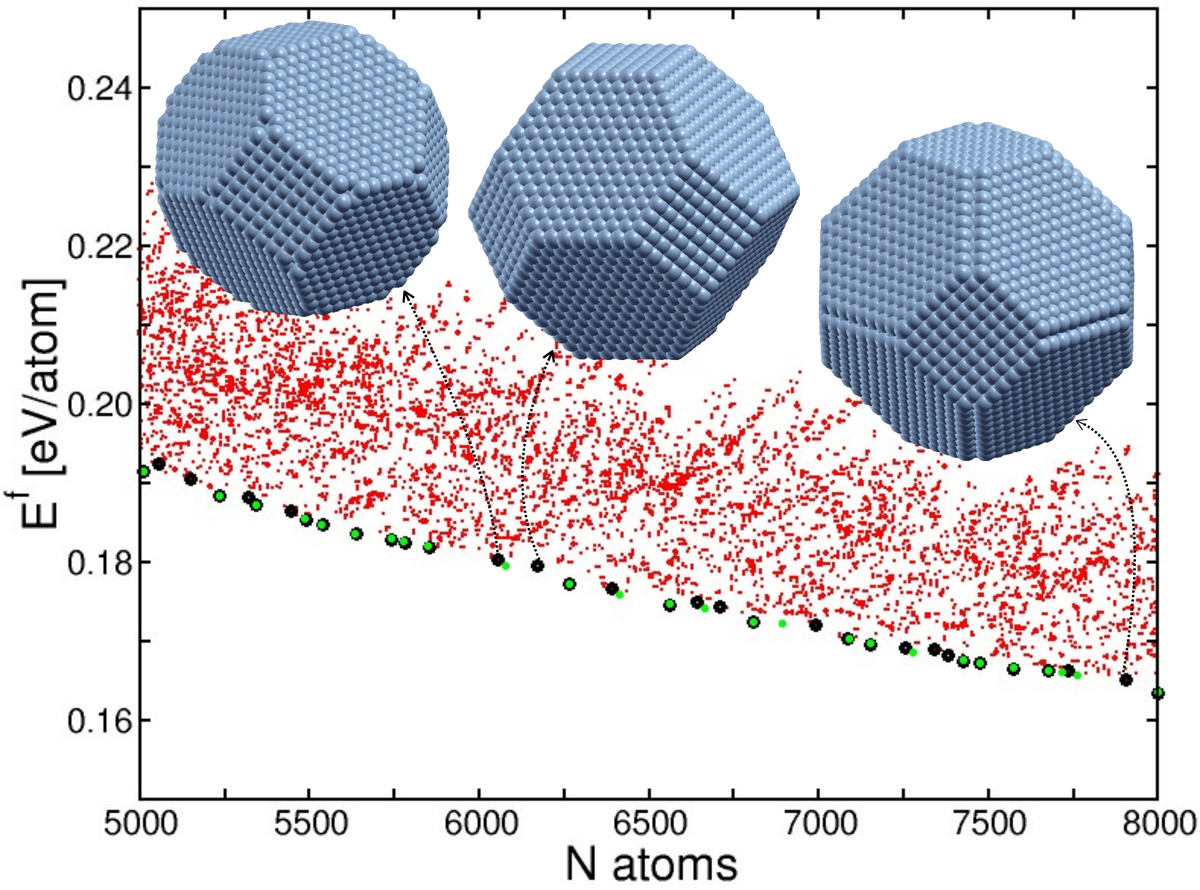

Figure S31: Formation energy of the clean nanoparticles in the range of N_{atoms} between 5000 and 8000 (red dots). High-probability nanoparticles in the absence and in the presence of CO* are highlighted with green and black circles, respectively. The crystal structure of selected nanoparticles is reported.

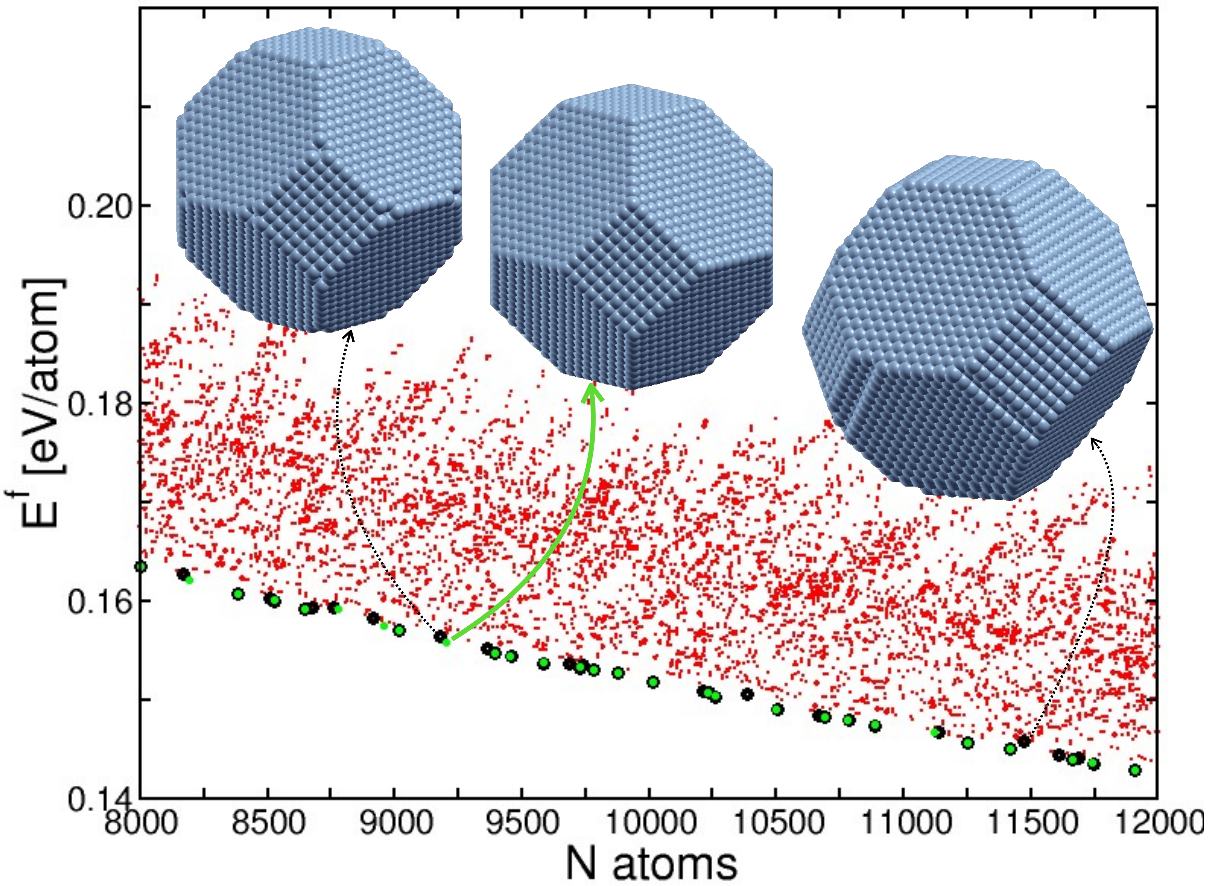

Figure S32: Formation energy of the clean nanoparticles in the range of N_{atoms} between 8000 and 12000 (red dots). High-probability nanoparticles in the absence and in the presence of CO* are highlighted with green and black circles, respectively. The crystal structure of selected nanoparticles is reported.

Several nanoparticles with high frequency of occurrence in absence of CO* show high frequency even in the presence of the adsorbate. Additional nanoparticles show high probability of occurrence when CO* is taken into account, most of them showing step-edge or step-corner sites. As shown for instance in Figure S30 or Figure S32, the difference between the shape of the nanoparticles in the two cases is very similar, differing only for the presence of stepped sites. This is further confirmed by looking at the total number of the active sites as a function of the diameter, reported in Figure S33-S35. The number of stepped sites is relevant especially above 3 nm and compensates for the slightly lower number of edge sites in the presence of CO* reported in FigureS33 and S34.

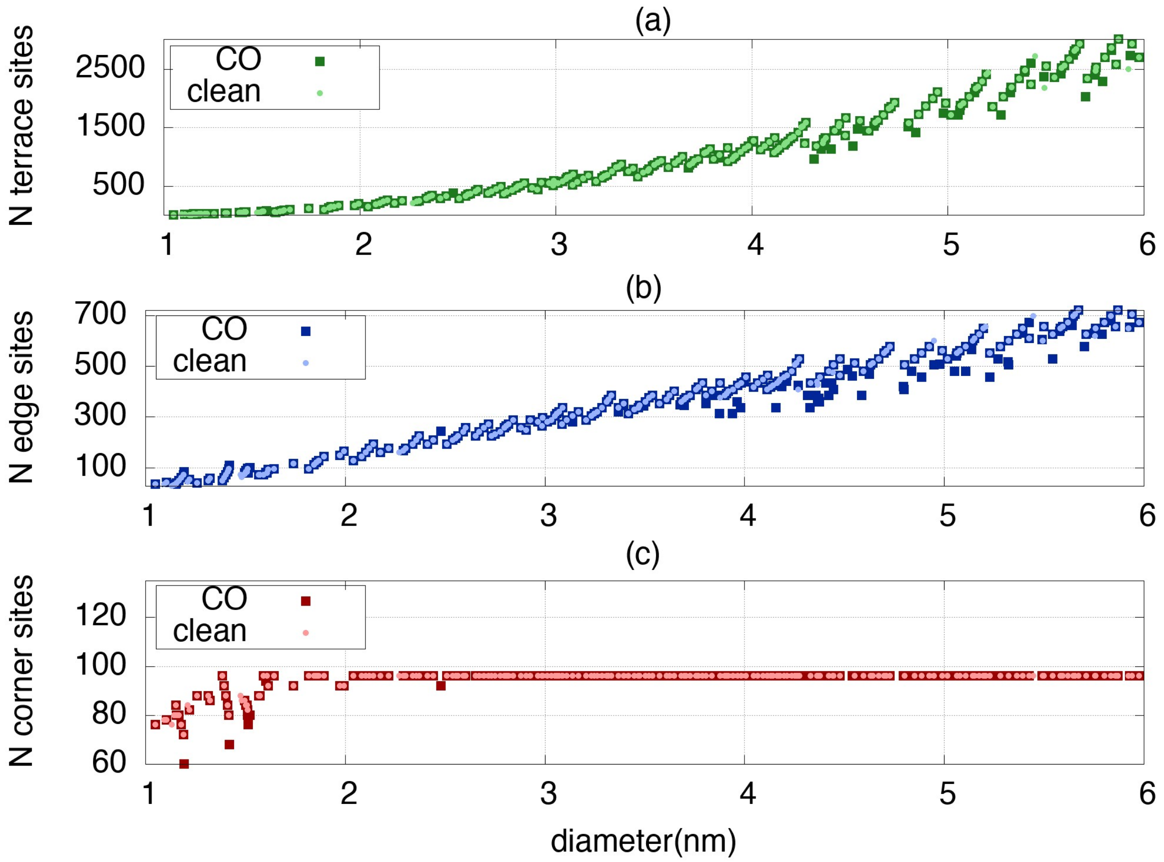

Figure S33: Total number of terrace (a), edge (b) and corner (c) three-fold coordinated sites as a function of the diameter for the high-probability nanoparticles in the presence and in the absence of CO*.

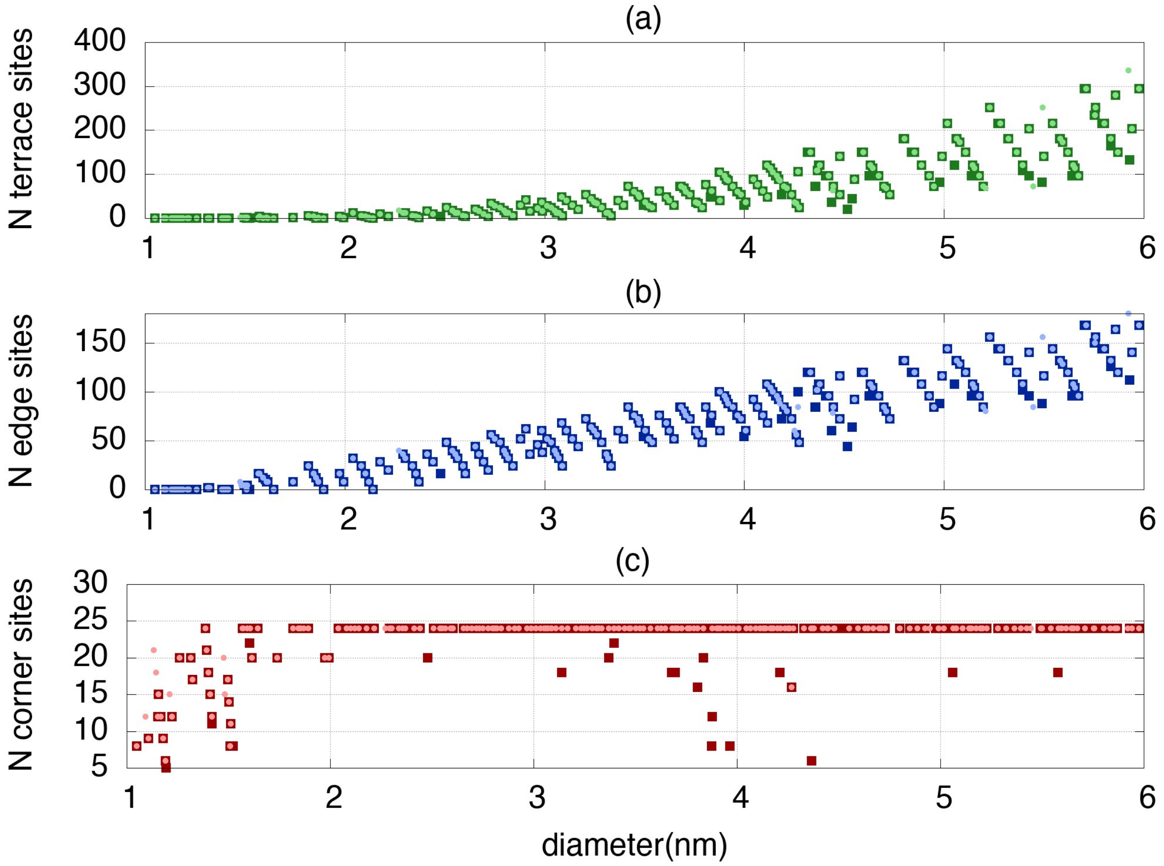

Figure S34: Total number of terrace (a), edge (b) and corner (c) four-fold coordinated sites as a function of the diameter for the high-probability nanoparticles in the presence and in the absence of CO*.

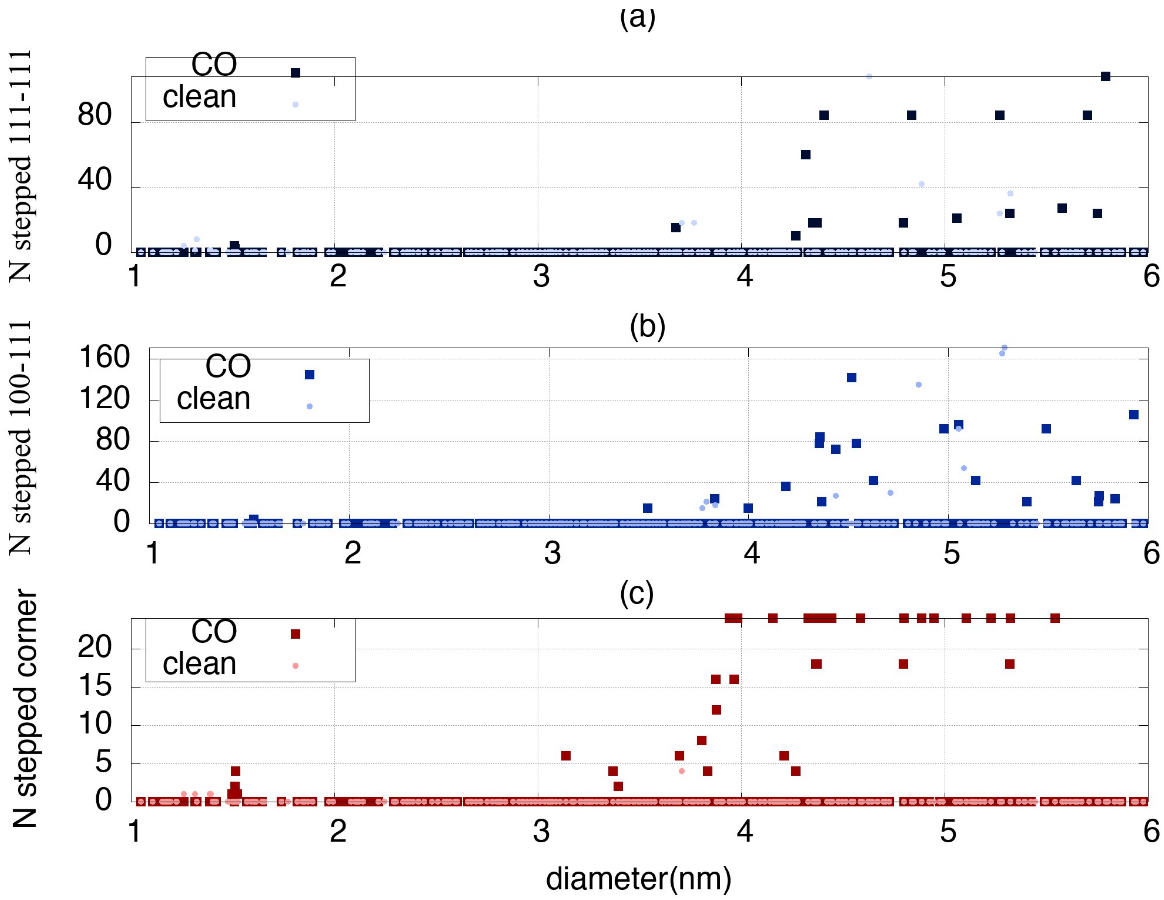

Figure S35: Total number of stepped sites at the (111)-(111) interface (a), (100)-(111) interface (b) and stepped corner sites (c) as a function of the diameter for the high-probability nanoparticles in the presence and in the absence of CO*.

7 TOF of the Wulff-constructed Ni nanoparticles

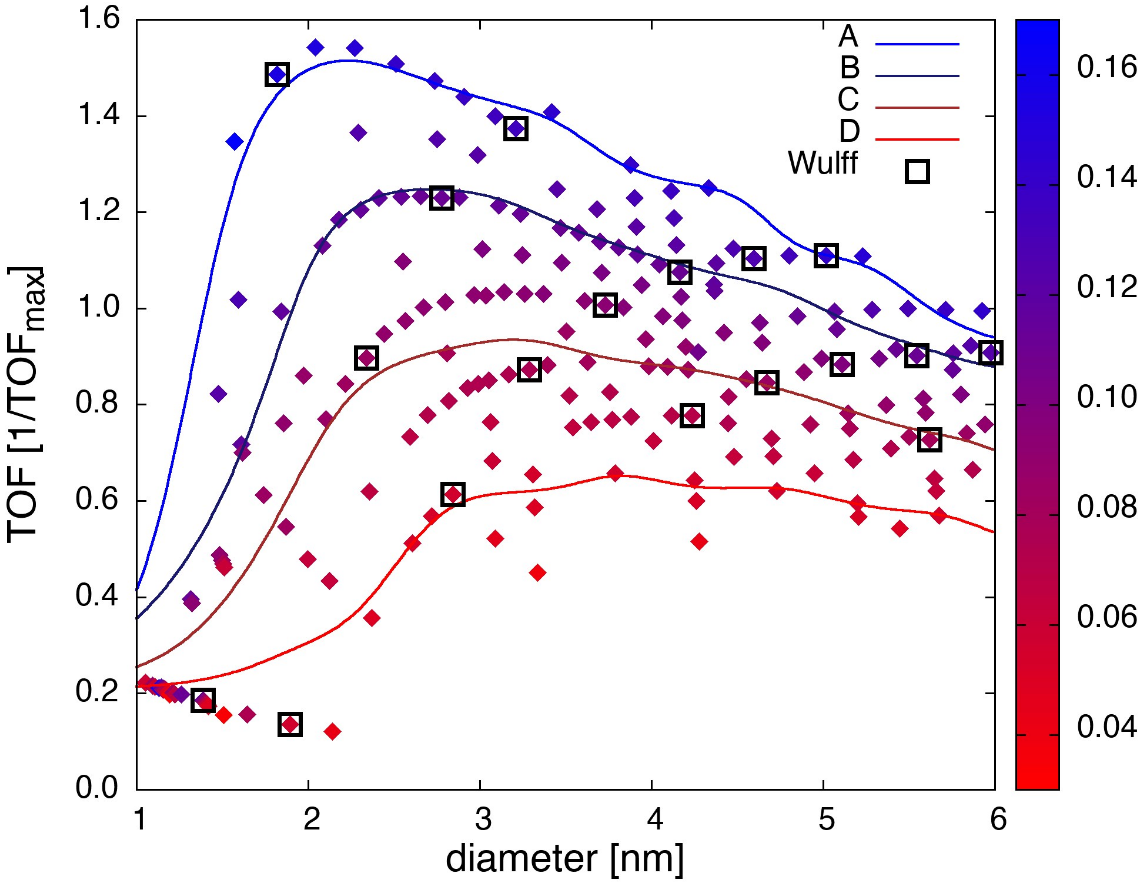

Figure S36: TOF of the high-probability metal nanoparticles as a function of the diameter (dots), represented in terms of the fourfold sites fraction, according to the color bar. The lines represent a gaussian smoothing of four different groups of metal nanoparticles, having similar fourfold sites fraction. Wulff-constructed metal nanoparticles are highlighted with black squares.

References

(S1) Sterk, E. B.; Nieuwelink, A.-E.; Monai, M.; Louwen, J. N.; Vogt, E. T. C.; Filot, I. A. W.; Weckhuysen, B. M. Structure Sensitivity of CO2 Conversion over Nickel Metal Nanoparticles Explained by Micro-Kinetics Simulations. *JACS Au* 2022, *2*, 2714– 2730. https://doi.org/10.1021/jacsau.2c00430

(S2) Vogt, C.; Monai, M.; Sterk, E. B.; Palle, J.; Melcherts, A. E. M.; Zijlstra, B.; Groeneveld, E.; Berben, P. H.; Boereboom, J. M.; Hensen, E. J. M.; Meirer, F.; Filot, I. A. W.; Weckhuysen, B. M. Understanding carbon dioxide activation and carbon– carbon coupling over nickel. *Nature Communications* 2019, *10*, 5330. https://doi.org/10.1038/s41467-019-12858-3

(S3) Kreitz, B.; Sargsyan, K.; Blöndal, K.; Mazeau, E. J.; West, R. H.; Wehinger, G. D.; Turek, T.; Goldsmith, C. F. Quantifying the Impact of Parametric Uncertainty on Automatic Mechanism Generation for CO2 Hydrogenation on Ni(111). *JACS Au* 2021, *1*, 1656–1673. https://doi.org/10.1021/jacsau.1c00276

(S4) Eyring, H. The Activated Complex and the Absolute Rate of Chemical Reactions. *Chemical Reviews* 1935, *17*, 65–77. https://doi.org/10.1021/cr60056a006

(S5) National Institute of Standard and Technology, U.S. Department of Commerce, NIST-JANAF Thermochemical Tables. https://janaf.nist.gov/, accessed November 12, 2019.

(S6) Mason, S. E.; Grinberg, I.; Rappe, A. M. First-principles extrapolation method for accurate CO adsorption energies on metal surfaces. *Phys. Rev. B* 2004, *69*, 161401. https://doi.org/10.1103/PhysRevB.69.161401

(S7) Vogt, C.; Groeneveld, E.; Kamsma, G.; Nachtegaal, M.; Lu, L.; Kiely, C. J.; Berben, P. H.; Meirer, F.; Weckhuysen, B. M. Unravelling structure sensitivity in CO2 hydrogenation over nickel. *Nature Catalysis* 2018, *1*, 127–134. https://doi.org/10.1038/s41929-017-0016-y

(S8) Cheula, R.; Maestri, M.; Mpourmpakis, G. Modeling Morphology and Catalytic Activity of Nanoparticle Ensembles Under Reaction Conditions. *ACS Catalysis* 2020, *10*, 6149–6158. https://doi.org/10.1021/acscatal.0c01005

(S9) Tománek, D.; Mukherjee, S.; Bennemann, K. H. Simple theory for the electronic and atomic structure of small clusters. *Phys. Rev. B* 1983, *28*, 665–673. https://doi.org/10.1103/PhysRevB.28.665

(S10) Yan, Z.; Taylor, M. G.; Mascareno, A.; Mpourmpakis, G. Size-, Shape-, and Composition-Dependent Model for Metal Nanoparticle Stability Prediction. *Nano Letters* 2018, *18*, 2696–2704. https://doi.org/10.1021/acs.nanolett.8b00670

(S11) Cheula, R.; Soon, A.; Maestri, M. Prediction of morphological changes of catalyst materials under reaction conditions by combined ab initio thermodynamics and microkinetic modelling. *Catal. Sci. Technol.* 2018, *8*, 3493–3503. https://doi.org/10.1039/C8CY00583D

(S12) H. Conrad, G. Ertl, J. Küppers, E.E. Latta, Adsorption of CO on clean and oxygen covered Ni(111) surfaces, *Surface Science* 1976, 57, 2, 475-484. https://doi.org/10.1016/0039-6028(76)90341-1

(S13) J. C. Tracy; Structural Influences on Adsorption Energy. II. CO on Ni(100), *J. Chem. Phys.* 1972; 56, 6, 2736–2747. https://doi.org/10.1063/1.1677602
